# A biomathematical model of human erythropoiesis and iron metabolism

**DOI:** 10.1038/s41598-020-65313-5

**Published:** 2020-05-25

**Authors:** Sibylle Schirm, Markus Scholz

**Affiliations:** Institute for Medical Informatics, Statistics and Epidemiology, University of Leipzig, Leipzig, Germany

**Keywords:** Computational models, Computer modelling

## Abstract

Anaemia therapy or perisurgical support of erythropoiesis often require both, EPO and iron medication. However, excessive iron medication can result in iron overload and it is challenging to control haemoglobin levels in a desired range. To support this task, we develop a biomathematical model to simulate EPO- and iron medication in humans. We combine our previously established model of human erythropoiesis including comprehensive pharmacokinetic models of EPO applications with a newly developed model of iron metabolism including iron supplementation. Equations were derived by translating known biological mechanisms into ordinary differential equations. Qualitative model behaviour is studied in detail considering a variety of interventions such as bleeding, iron malnutrition and medication. The model can explain time courses of erythrocytes, reticulocytes, haemoglobin, haematocrit, red blood cells, EPO, serum iron, ferritin, transferrin saturation, and transferrin under a variety of scenarios including EPO and iron application into healthy volunteers or chemotherapy patients. Unknown model parameters were determined by fitting the predictions of the model to time series data from literature. We demonstrate how the model can be used to make predictions of untested therapy options such as cytotoxic chemotherapy supported by iron and EPO. Following our ultimate goal of establishing a model of anaemia treatment in chronic kidney disease, we aim at translating our model to this pathological condition in the near future.

## Introduction

Anaemia is a frequent disease condition of different etiology such as cytotoxic chemotherapy, kidney disease and chronic inflammation. Treatment options in case of severe anaemia comprise erythrocyte transfusion, EPO application and iron supplementation. However, the latter is contra-indicated for some types of anaemia such as thalassemia or haemolytic anaemia and it is not recommended in case of severe infections. Anaemia treatment options result in a complex dynamic behaviour of iron metabolism, bone marrow erythropoiesis, cells in circulation, haemoglobinization of red blood cells and cytokine levels that is difficult to predict.

To optimize treatment schedules of anaemia, we developed a biomathematical model of human erythropoiesis^1,2^ in the past. The model describes the dynamics of bone marrow erythropoiesis and circulating cells under chemotherapy and EPO applications considering cytotoxic effects of different drugs and absorption, pharmacokinetics and -dynamics of a variety of EPO derivatives and application schedules. However, iron metabolism was not considered so far. To close this gap, we here aim at developing a biomathematical model of iron metabolism and combine it with our erythropoiesis model. For this purpose, we consider key biological features of iron homoeostasis and translate them into ordinary differential equations. The model is studied under different conditions such as single and combined EPO and iron applications, iron malnutrition, blood donation and cytotoxic chemotherapy. Model predictions are quantitatively compared to available time series data of laboratory parameters measured under these conditions including red blood cell parameters, plasma iron parameters and erythropoietin levels.

## Results

**Figure 1:**
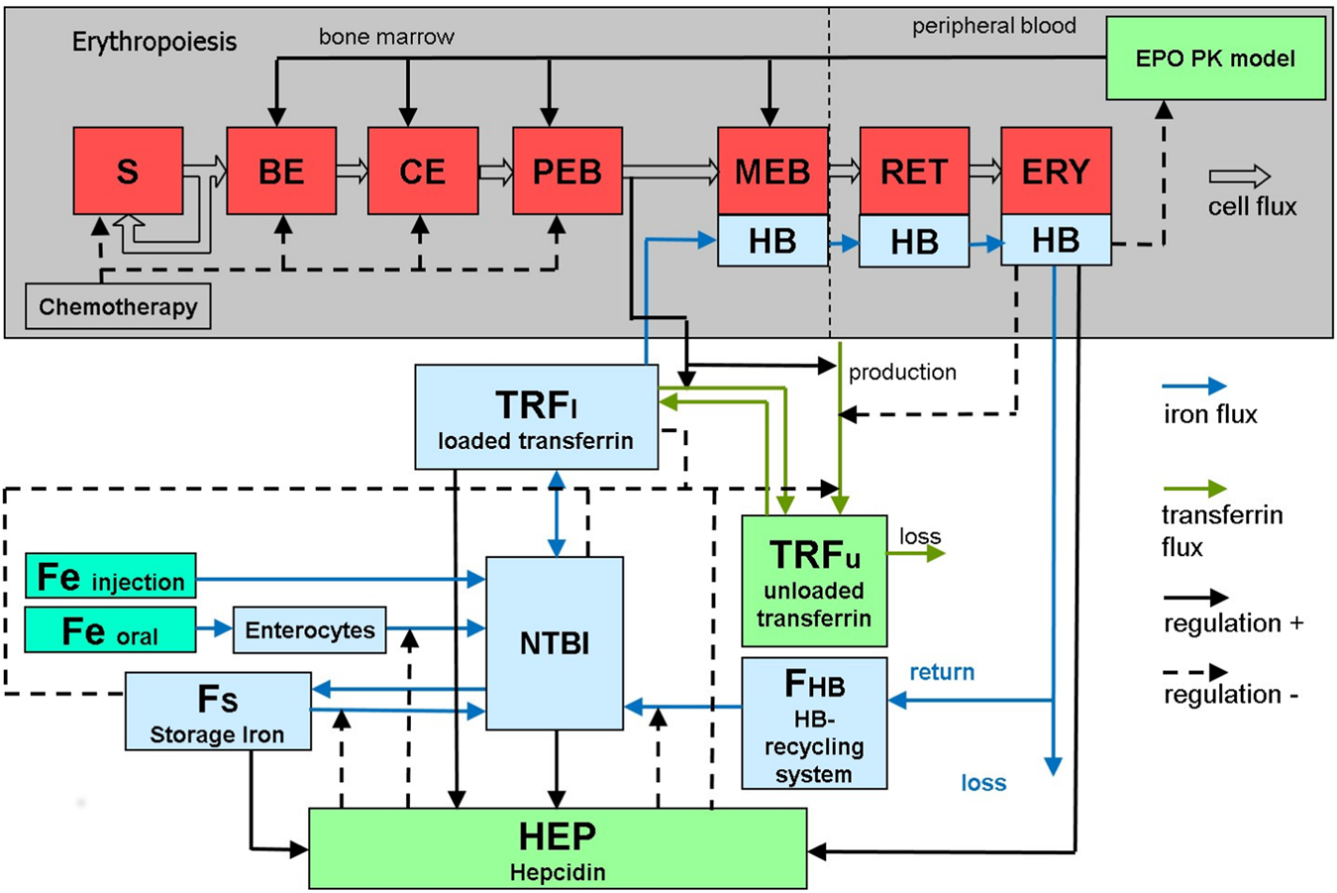
Structure of the combined iron and erythropoiesis model. The model consists of our formerly developed erythropoiesis model, an EPO PK-PD model, an EPO absorption model and a model of iron metabolism developed here. It describes the dynamics of erythropoietic cell lineage including stem cells (S), burst forming unit erythroid (BE), colony forming unit erythroid (CE), proliferating erythrocytic blasts (PEB), maturing erythrocytic blasts (MEB), reticulocytes (RET) and erythrocytes (ERY). EPO regulates proliferation and maturation of red blood cells in the bone marrow. Chemotherapy reversibly reduces the number of erythropoietic bone marrow cells^1^. The newly developed model of iron metabolism consists of the compartments hepcidin HEP, non-transferrin bound iron in plasma (NTBI), the haemoglobin-catabolic system F_HB_, storage iron F_S_, iron loaded transferrin TRFl and free transferrin TRFu. Iron absorption of bone marrow cells depends on the efflux of the compartment PEB and the iron supply via transferrin. The endogenous production of EPO is regulated by haemoglobin (HB), which is calculated from the iron content of the erythrocytes. Iron is recycled from haemoglobin of dying erythrocytes. Orally administered iron is absorbed via enterocytes (${{\rm{F}}}_{{\rm{e}}{\rm{n}}{\rm{t}}{\rm{e}}{\rm{r}}{\rm{o}}}$) and in dependence on ferroportin (not shown as compartment).

Our iron model consists on ordinary differential equations of the following biological quantities: storage iron, plasma transferrin, non-transferrin bound iron, iron stored in cells of the red blood cell lineage and iron in enterocytes. The system is regulated by hepcidin, which blocks ferroportin, and with it, iron fluxes between compartments. Model compartments are presented in Table 1. The model is linked to our former erythropoiesis model by affecting haemoglobinization of red blood cell precursors and considering the recycling of iron by the haemoglobin catabolic system (see Fig. 1). Model assumptions and equations are described and discussed in detail in the methods section. The model contains a number of unknown parameters, which are either determined by biological assumptions, steady state conditions or estimated by fitting the predictions of the model to available data. Details of the fitting process can also be found in the methods section. Model parameters and equations of the erythropoiesis model are listed in the supporting file 1 (“ERYFe_supp1.pdf”). Fitted parameters result in a constant stable steady state of the system.

**Table 1:** Compartments.

Compartment	Meaning
S	stem cells
BE	burst forming unit erythroid
CE	colony forming unit erythroid
PEB	proliferating erythrocytic blasts
MEB	maturing erythrocytic blasts
RET	reticulocytes in circulation
ERY	mature erythrocytes in circulation
EPO	erythropoietin concentration
HEP	hepcidin in plasma
NTBI	non-transferrin bound iron in plasma
F_HB_	haemoglobin recycling
${F}_{S}$	storage iron
TRFl	iron loaded transferrin
TRFu	free transferrin
${{\rm{F}}}_{{\rm{e}}{\rm{n}}{\rm{t}}{\rm{e}}{\rm{r}}{\rm{o}}}$	iron in enterocytes

### Model behaviour

We simulate a number of scenarios to evaluate the qualitative performance of the model.

#### Oral iron application in healthy individuals

Oral iron application is modelled by an influx of additional iron into enterocytes. Here we simulate the application of 150 mg iron two times a day with time distance of 12 h over a period of 50 days. As a result, the numbers of erythrocytes and reticulocytes decrease while the haemoglobin level increases. The levels of serum iron, ferritin and transferrin saturation also increase continuously, whereas serum transferrin and ferroportin decrease, and the iron content of the recycling system increases (see Fig. S1 in the Supplemental file 2).

#### Intravenous EPO injection with oral iron application in healthy individuals

We simulated the effect of oral iron application (300 mg two times a day (day 0–9)) after a single intravenous injection of 200 IU/kg EPO on day 0 in comparison to EPO application alone. After EPO injection, one can observe an increase of erythropoietic cells. Under supporting iron medication, the numbers of erythrocytes, reticulocytes and the level of EPO differ only slightly from those without iron application, while haemoglobin levels are higher. EPO alone results in decreasing iron levels in serum and tissue. This deficiency is less pronounced under supportive iron supplementation, i.e. serum iron, transferrin saturation and plasma ferritin are higher under iron support, and the transferrin level is less elevated. Ferroportin increases after EPO application but less strongly under additional oral iron support (see Fig. S2 in the Supplemental file 3).

#### Iron deficiency

In this scenario, we simulate chronic iron malnutrition over a longer period. In detail, we assume that only 50% of the required daily iron uptake is available from food. Under iron deficiency, the numbers of erythrocytes, reticulocytes, and the EPO level rise. The haemoglobin level decreases. With respect to iron metabolism, levels of serum iron, ferritin and transferrin saturation decrease, whereas serum transferrin level increases. In accordance to Theurl *et al*.^3,4^, iron deficiency leads to a lower hepcidin levels and elevated ferroportin to increase iron uptake and recycling (see Fig. S3 in the Supplemental file 2).

#### Intravenous injection of iron in healthy individuals

Now, the effect of an intravenous iron injection of 100 mg iron is simulated. In analogy to oral iron application, numbers of erythrocytes, reticulocytes, and the level of EPO decrease, while the haemoglobin level increases and remains elevated for a longer period. Iron injection rapidly increases the levels of serum iron, ferritin and transferrin saturation, followed by a return to normal levels, whereas serum transferrin decreases. The hepcidin level increases and ferroportin decreases reducing iron uptake from enterocytes and iron recycling (see Fig. S4 in the Supplemental file 2).

#### Bleeding/Phlebotomy

Bleeding is modelled by an instantaneous loss of 10% of the blood volume. In consequence, numbers of erythrocytes and reticulocytes are reduced and the haemoglobin level decreases. Due to the endogenous EPO feedback, more reticulocytes are released after a short period and their numbers eventually exceed steady state values. Erythrocyte recovery is delayed. The haemoglobin level is continuously recovering but the steady state value is not achieved over the simulated period of 100 days. Serum iron, ferritin and transferrin saturation are rapidly decreased after the bleeding event followed by a slow regeneration without reaching their steady state levels over the simulated period. Transferrin production is elevated and hepcidin is decreased (see Fig. S5 in the Supplemental file 2).

#### Chronic inflammation

Chronic inflammation increases hepcidin levels^3–6^. This is modelled by a constant additional influx into the hepcidin compartment. Under this condition, numbers of erythrocytes, reticulocytes and the level of EPO rise, while the haemoglobin level decreases over the simulated period. Serum iron, ferritin and transferrin saturation are reduced due to the increased blocking of ferroportin, and with it, all routes of iron fluxes including the recycling by macrophages. The iron levels in the storage compartments, enterocytes and the recycling system are elevated (see Fig. S6 in the supplemental file 2).

#### Haemochromatosis

Haemochromatosis is characterized by decreased hepcidin levels^7^. This is modelled by a lower influx into the hepcidin compartment. Under this condition, numbers of erythrocytes, reticulocytes and the level of EPO decrease, while the haemoglobin level increases over the simulated period. Serum iron, ferritin, storage iron and transferrin saturation are elevated due to the increase of ferroportin. The iron levels in enterocytes and the recycling system decrease due to the higher absorption and recycling rate (see Fig. S7 in the Supplemental file 2).

### Comparison of model and data

We now quantitatively compare our model predictions with available clinical data obtained under different treatment conditions. Regarding initial conditions, we either used available measurements at day 0 or the values presented in Tables S3 and S8 in supplemental file 1, which are taken from the literature.

**Figure 2:**
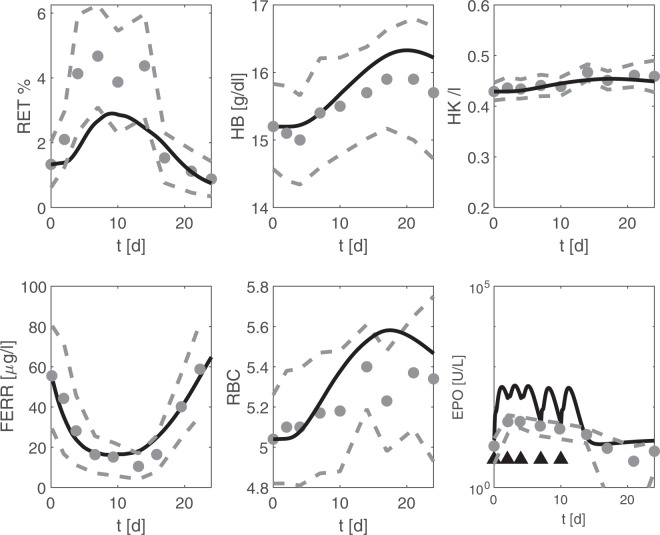
Comparison of model and data for EPO application without iron supplementation. We consider EPO application into male athletes as studied in Souillard *et al*.^8^ and compare model predictions (black line) with the corresponding averaged data (grey). The grey dots represent means of athletes. Grey dashed lines represent mean ± one standard deviation, and triangles denote the time points of EPO injections.

First, Souillard *et al*.^8^ examined pharmacokinetics of EPO. Their study includes 20 healthy male athletes receiving 200 IU/kg EPO on days 0, 2, 4, 7, and 10, without iron medication. We simulate this scenario and compare it to the corresponding data (Fig. 2). Simulation results of reticulocytes, haemoglobin, red blood cells, haematocrit, ferritin and serum EPO are in good agreement with the data.

**Figure 3:**
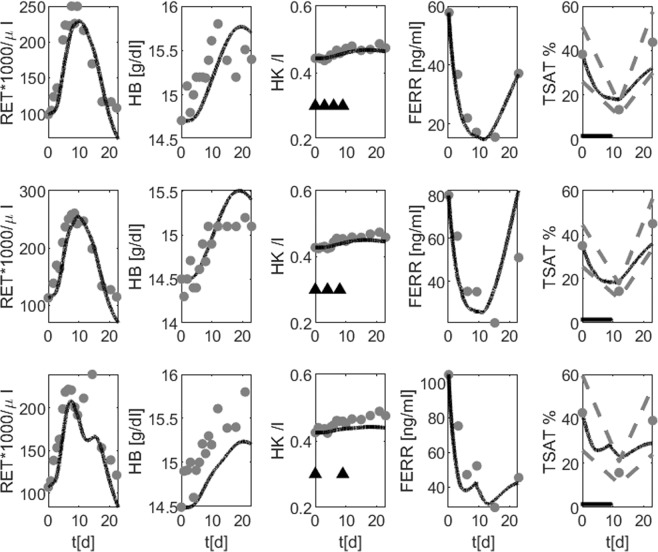
Comparison of model and data for EPO application with iron supplementation. Group 1 (first row) received 300 IU/kg of EPO on days 0, 3, 6, and 9. Group 2 (second row) received 400 IU/kg of EPO on days 0, 4, and 8. Group 3 (third line) received 600 IU/kg of EPO on days 0 and 9. All Groups received 300 mg of iron orally for 10 days beginning at day 0. Simulation results of reticulocytes, haemoglobin, haematocrit, ferritin and transferrin saturation are shown (black row). Data from^9^ are depicted as grey dots. Grey dotted lines represent mean ± one standard deviation if available. Triangles denote the time points of EPO injections, iron medication is denoted by plus.

Second, Rutherford *et al*.^9^ examined the effectiveness of EPO in preoperative use. Their study included 24 healthy male subjects receiving 1200 IU/kg EPO subcutaneously in different time schedules with supportive iron supplementation of 300 mg oral iron daily for the first 10 days. EPO is given until day 9. Due to its quick elimination, EPO is almost removed a day later. Therefore, bone marrow stimulation occurs until day 10 and drops thereafter. Accordingly, reticulocytes increase until day 10 and normalize thereafter. Hemoglobin and hematocrit react with some delay. TSAT and ferritin drop until day 10 due to increased iron consumption by the intensified erythropoiesis. Afterwards, these levels normalize due to the iron recycling system and the increased uptake from enterocytes. Simulation results of reticulocytes, haematocrit, ferritin and transferrin saturation are in good agreement to the corresponding data (Fig. 3). Haemoglobin levels are underestimated especially for the highest dose. Unfortunately, error bars are not available for these data.

**Figure 4:**
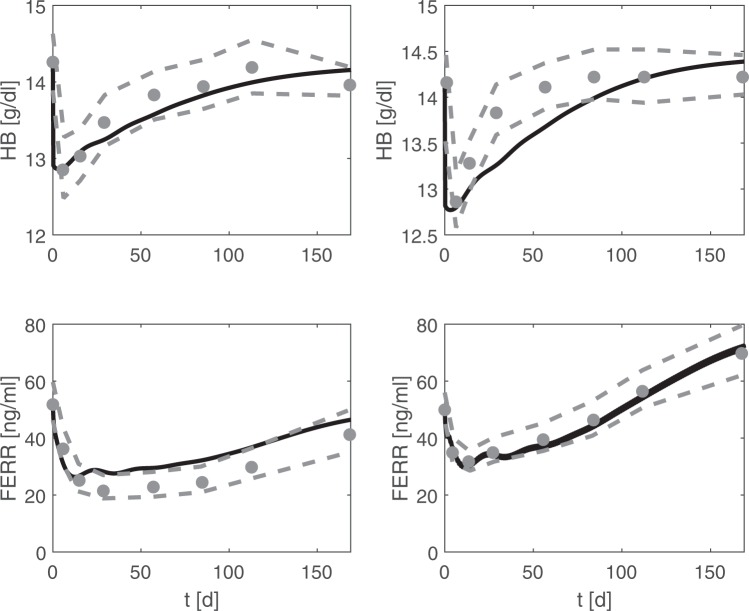
Recovery of erythropoiesis after phlebotomy: Comparison of model and data. Simulation results of haemoglobin and ferritin after blood donation (black line, left panels: without iron medication, right panels: with daily oral iron support) are compared with corresponding averaged data from Kiss *et al*.^10^. Grey dots represent means of study individuals. Grey dashed lines represent mean ± one standard deviation.

Finally, Kiss *et al*.^10^ studied regeneration of erythropoiesis after donation of 500 ml blood with or without iron medication. Study individuals were assessed over a period of 24 weeks. Model simulations of both scenarios are in good agreement with measured haemoglobin and ferritin data (Fig. 4).

### Model prediction

**Figure 5:**
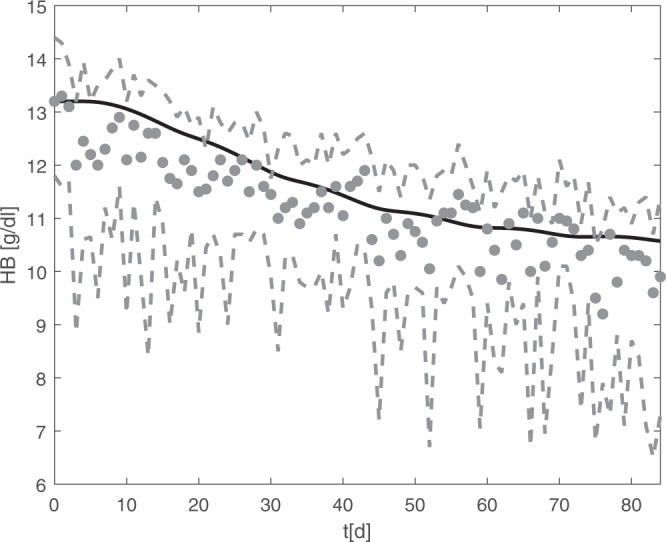
Erythropoiesis under CHOP-14 chemotherapy. Simulation results of haemoglobin during six cycles of CHOP-14 chemotherapy (black line) are shown. Grey dots and dashed lines correspond to patients medians (N = 174) and interquartile ranges, respectively. Data were obtained from studies of the German High-Grade Non-Hodgkin’s Lymphoma Study Group^11,15^.

After establishing the model, it is possible to make predictions regarding yet untested therapy options including EPO and iron. We demonstrate this on a chemotherapy of six cycles CHOP (cyclophosphamide, doxorubicin, vincristine, and prednisone), the standard care of aggressive non-Hodgkin’s lymphoma. Application of the granulopoietic growth factor G-CSF allowed application of this chemotherapy in 14 days intervals with improved outcome^11^. However, this dose dense therapy results in increased anaemia risk^12^. So far, EPO and iron application during this therapy is not standard but has potential to ameliorate this side effect^13,14^. Toxicity of chemotherapeutic drugs is modelled by a delayed and transient depletion of the proliferating erythropoietic bone marrow cells. Toxic effects on maturing cells are neglected. We simulated standard CHOP-14 with our model and compared the results with patients data from the German High-Grade Non-Hodgkin’s Lymphoma Study Group^11,15^. Results are shown in Fig. 5, with good agreement of model and data.

**Figure 6:**
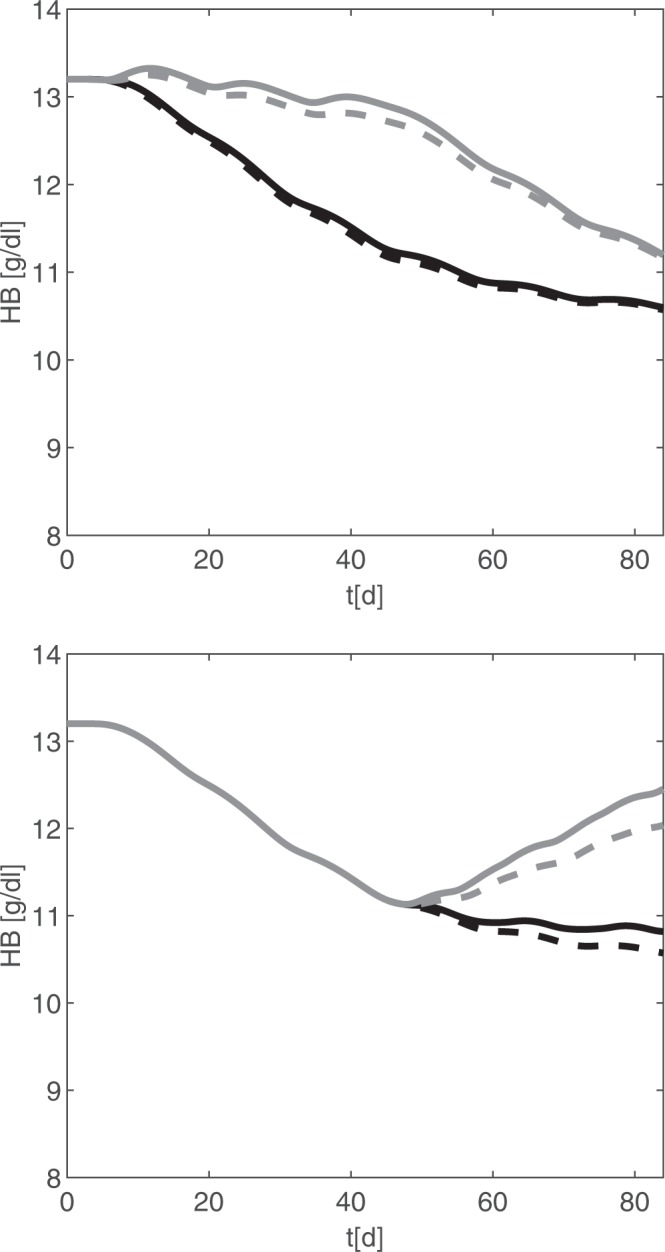
Model simulation of six cycles CHOP-14 with or without EPO or iron support. Simulation results of haemoglobin under CHOP-14 chemotherapy standard care (without EPO and iron, dashed black line), additional intravenous application of iron (100 mg, solid black line), intravenous injection of 40000 IU EPO (equals about 500–600 IU/kg, as recommended in^13^, grey dashed line), and the combination of iron and EPO treatment (grey solid line). Upper panel: (Prophylaxis): iron and EPO are given at days 3, 17, 31. Lower panel (Amelioration): iron and EPO weekly applied, starting at day 45.

To evaluate the beneficial potential, we simulate supportive intravenous iron- and EPO applications and combinations thereof during CHOP-14 chemotherapy. We first simulated prophylactic iron and EPO applications Fig. 6A. We also simulated the recommendation of Auerbach *et al*.^13^ to start treatment when hemoglobin levels drop below 10.5 g/dl, which occurs in the fourth chemotherapy cycle according to our simulations Fig. 6B. We predict that supportive treatment with iron and EPO has strong potential in preventing or ameliorating anaemia in these patients but the strongest improvement is due to EPO rather than iron supplementation. Effect of iron application is stronger in scenario B.

## Discussion

In the present paper, we propose a comprehensive ordinary differential equations model of human iron metabolism and its impact on erythropoiesis. For this purpose, we combined our previously developed model of human erythropoiesis under chemotherapy and EPO applications^1^ with a newly proposed model of iron homoeostasis and treatment. Model equations are based on known biological knowledge and are parametrized on the basis of clinical data. Qualitative and quantitative performance of the model were studied in detail. We also provide predictions regarding yet untested supportive treatment of patients under chemotherapy with iron, EPO and combinations of it.

Rather then building a model from scratch, we follow a multi-stage approach by stepwise increasing the complexity of our model. In our former work^1^, we combined a cell kinetic model of bone marrow erythropoiesis^16–21^, a pharmacokinetic model of EPO applications^22^, a model of EPO absorption after subcutaneous injection^23^ and a model of chemotherapy toxicity derived from granulopiesis modelling^24,25^. We now add the impact of iron metabolism without changing the already established equations.

A few models of iron homoeostatic were proposed in the literature. A differential equations model of iron metabolism was proposed about 40 years ago^26,27^. New insights into regulatory mechanisms mediated by e.g. hepcidin and ferroportin were not available at this time and need to be considered now especially in case of inflammatory conditions such as chronic kidney disease (CKD). The underlying molecular network is complex^28^, so that some investigators develop models describing specific parts of iron metabolism, such as mathematical models of the dynamics of iron storage in ferritin *in vitro*^29^, hepcidin regulation^5^ or iron release from macrophages^30^. An overview and comparison of models concerning the relationship between EPO, soluble transferrin receptors and ferritin was provided by Bressolle *et al*.^31^. Lao *et al*.^32^ developed a compartmental model of iron regulation in mice considering hepatocytes, hepcidin and ferroportin. Enculescu *et al*.^33^ built a model of the regulation of organ iron pools in mice under iron overload or inflammation. Another compartmental model of iron distribution in mice was developed by Parmar *et al*.^7,34^. The most comprehensive model of iron metabolism developed so far was proposed by Sarkar *et al*.^35^. This model is parameterized mainly on the basis of experimental mice data.

In our research, we focused on the human situation aiming at incorporating available biological and clinical data. The intended major field of application is to study treatment effects such as chemotherapy induced anaemia or stabilizing haemoglobin levels in CKD patients by EPO- and iron applications. Therefore, our model describes the dynamics of key players of iron metabolism non-transferrin bound iron, ferritin, transferrin, iron stored in liver and in red blood cells as well as their key regulators hepcidin and ferroportin. We also consider iron loss due to bleeding and excretion, iron replenishment by diet, and, intravenous or oral iron supplementation including absorption of oral iron via enterocytes. Iron storage in erythrocytes and their recycling is modelled by iron compartments which are analogously regulated as respective cellular compartments.

Model equations are derived from knowledge of the physiology of human iron metabolism. Some simplifications were made, either to reduce the number of unknown parameters of the model or due to lack of human data for corresponding model compartments. As example, we do not distinguish between Fe2+ and Fe3+ due to lack of data. Likewise, we do not distinguish between transferrin loaded with one or two iron ions. Moreover, while hepcidin and ferroportin are explicitly modelled, the associated efferent and afferent regulations are only modelled phenomenologically via sigmoidal functions.

The majority of model parameters are defined by assuming a fixed steady state of iron homoeostasis. Remaining 24 free parameters were determined by fitting model predictions to clinical data of patients or volunteers under EPO and iron supplementation or after bleeding. Reliability of parameter estimates were assessed via a comprehensive sensitivity analysis. We obtained a reasonable quantitative agreement of model and data except for an underestimation of haemoglobin levels in a few instances. Regarding qualitative agreement, the model explains recovery times after blood donation which are reported to be in the order of 150 days in accordance to Kiss *et al*.^10^.

A major future application of the model is to describe pathomechanisms and disease courses of CKD. However, this requires additional assumptions for example with respect to reduced EPO production in the kidneys, blood loss due to micro-bleeding, reduced life-time of erythrocytes, the effect of chronic inflammation on the iron metabolism and the effects of continuous treatment with oral or intravenous iron or EPO. We will address these issues in a future study.

## Methods

We construct a comprehensive model of human erythropoiesis and iron homoeostasis by combining the iron metabolism model to be developed in the present paper with our previously developed cell-kinetic model of bone marrow erythropoiesis including pharmacokinetics and -dynamics of several EPO derivatives and routes of application^1,2^. We briefly sketch the structure of the latter model.

### Erythropoiesis Model

The cell kinetic model of erythropoiesis describes the dynamics of erythropoietic cell lineages in bone marrow and circulation, i.e. stem cells (S), burst forming units (BE), colony forming units (CE), proliferating erythrocytic blasts (PEB), maturing erythrocytic blasts (MEB), reticulocytes (RET) and erythrocytes (ERY). Erythrocytes are age-dependently removed from circulation. The regulatory cytokine erythropoietin (EPO) is explicitly modelled. It increases proliferation and shortens maturation of erythroid bone marrow cells. To model external EPO applications of different pharmaceutical derivatives via different routes of application, we included both, a model of EPO absorption adapted from^23^ and a pharmacokinetic (PK) model adapted from^22^. The PK model consists on the compartments EPO in central serum (${C}_{{\rm{EPO}}}^{{\rm{cent}}}$), a peripheral compartment (${C}_{{\rm{EPO}}}^{{\rm{peri}}}$) with first order transitions describing reversible protein binding, and a compartment for receptor bound EPO (${C}_{{\rm{EPO}}}^{{\rm{rb}}}$) by which EPO is internalized and pharmacodynamically active (${C}_{{\rm{EPO}}}^{{\rm{int}}}$). As improvement of our previous model^1^, the endogenous production of EPO is regulated by the haemoglobin content of the erythrocytes. Chemotherapy temporarily reduces counts of bone marrow cells in dependence on drug and dosage. Table 1 contains a list of all relevant compartments of the erythropoiesis model. More details can be found in^1^.

In the present paper, we keep this model essentially unchanged and attach the model of iron metabolism developed here.

### Model of Iron Metabolism and Adaptations of the Erythropoiesis Model

We here introduce biological assumptions and corresponding equations for the model of iron metabolism to be developed. We also describe how it is attached to our erythropoiesis model. Most biological assumptions are retrieved from^36^.

Since free iron is toxic, iron in the body is always bound to proteins. Transferrin is responsible for carrying iron to the bone marrow to support haemoglobin synthesis in erythroid precursors. Iron is recycled from dying erythrocytes via the haemoglobin-catabolic system. Excess iron is stored in the liver in the form of ferritin^37^. Since iron binding capacity is limited^26^ and iron once absorbed can hardly be eliminated^28^, iron uptake is tightly regulated by the hormone hepcidin which suppresses the iron transporter ferroportin effectively blocking iron uptake from the intestine, iron activation from the liver and iron recycling. We translate these processes into ordinary differential equations in the following. Considered model compartments are presented and explained in Table 1. If not stated otherwise, model compartments always represent relative values, i.e. compartments are equal to one in steady state.

### Regulators of iron metabolism

We first introduce regulatory mechanisms which are relevant for several compartments of the iron model: To avoid iron overload, the hormone hepcidin regulates the absorption of iron from diet and the release from cells^36^. This is achieved by blocking Ferroportin which is responsible for iron transport from the inside of a cell to the outside. Hepcidin is produced in the liver. Higher levels of iron in circulation and tissue or inflammation increase hepcidin production. Conversely, intensified erythropoiesis, iron deficiency, and tissue hypoxia decrease it^38,39^.

#### Hepcidin

We assume an increased but saturated production of hepcidin (HEP) in dependence on the level of plasma iron *F*_*P*_ which consists of non-transferrin bound iron and iron loaded transferrin (${F}_{P}={k}_{{\rm{FeTRF}}}\cdot {\rm{TRFl}}+{\rm{NTBI}}$), storage iron *F*_*S*_, or higher oxygen saturation^38^, which is associated with higher haemoglobin levels *HB*:

41 $$\begin{array}{rcl}\frac{d{\rm{HEP}}}{dt} & = & {{\rm{HEP}}}_{{\rm{\max }}}-({{\rm{HEP}}}_{{\rm{\max }}}-{{\rm{HEP}}}_{{\rm{\min }}})\cdot \exp \left(-\,\log \left(\frac{{{\rm{HEP}}}_{{\rm{\max }}}-{{\rm{HEP}}}_{{\rm{\min }}}}{{{\rm{HEP}}}_{{\rm{\max }}}-{{\rm{HEP}}}_{{\rm{nor}}}}\right)\cdot {A}^{{{\rm{HEP}}}_{{\rm{b}}}}\right)\\  &  & -{d}_{{\rm{HEP}}}\cdot {\rm{HEP}}+{{\rm{HEP}}}_{{\rm{inflammation}}},\end{array}$$

where *A* summarizes the contributions of the above mentioned regulators of hepcidin levels:

$$A=\frac{{k}_{{\rm{HEPFS}}}\cdot {F}_{S}+{k}_{{\rm{HEPFP}}}\cdot {F}_{P}+{k}_{{\rm{HEPHB}}}\cdot {\rm{HB}}/{{\rm{HB}}}^{{\rm{nor}}}}{{k}_{{\rm{HEPFS}}}+{k}_{{\rm{HEPFP}}}+{k}_{{\rm{HEPHB}}}}.$$

In case of systemic inflammation or infection, hepcidin production is further increased^38^. This is modelled by an additional constant influx HEP_inflammation_ into the hepcidin compartment. We include an unspecific elimination rate *d*_HEP_ whose value is defined by the steady state condition:

$${d}_{{\rm{HEP}}}=\,\frac{{{\rm{HEP}}}_{{\rm{nor}}}}{{{\rm{HEP}}}_{0}}\mathrm{}.$$

#### Ferroportin

The decreasing sigmoidal function Zferro(HEP) depends on hepcidin and serves as a phenomenological description of ferroportin efficacy

42 $${\rm{Zferro}}({\rm{HEP}})={{\rm{Zferro}}}_{{\rm{\max }}}-({{\rm{Zferro}}}_{{\rm{\max }}}-{{\rm{Zferro}}}_{{\rm{\min }}})\cdot \exp \left(-\,\log \left(\frac{Z{{\rm{ferro}}}_{{\rm{m}}{\rm{a}}{\rm{x}}}-Z{{\rm{ferro}}}_{{\rm{m}}{\rm{i}}{\rm{n}}}}{Z{{\rm{ferro}}}_{{\rm{m}}{\rm{a}}{\rm{x}}}-Z{{\rm{ferro}}}_{{\rm{n}}{\rm{o}}{\rm{r}}}}\right)\cdot {{\rm{HEP}}}^{{{\rm{Zferro}}}_{{\rm{b}}}}\right)\mathrm{}.$$

Ferroportin is responsible for the release of iron from storage (hepatocytes), the influx from enterocytes and the recycling of haemoglobin^36^, i.e. in case of elevated hepcidin levels, iron fluxes of all three routes are reduced.

### Iron compartments

#### Exogenous iron supply

Three sources of exogenous iron support are considered in our model. First, iron is absorbed from the diet with rate ${{\rm{FeDiet}}}_{{\rm{nor}}}$. Second, iron can be supplemented via oral medication with rate $\frac{{{\rm{Fe}}}^{{\rm{oral}}}}{{{\rm{Zferro}}}_{{\rm{\min }}}}$. ${{\rm{Zferro}}}_{{\rm{\min }}}$ is a normalizing factor guaranteeing that the absorbed iron is smaller than the orally applied dose. Finally, iron can be directly injected into circulation ${{\rm{Fe}}}^{{\rm{inj}}}$.

Intravenous injection of iron ${{\rm{Fe}}}^{{\rm{inj}}}(t)$ is modelled as a sum of pulse functions

43 $${{\rm{Fe}}}^{{\rm{inj}}}(t)=\mathop{\sum }\limits_{i\mathrm{=1}}^{N}\frac{{{\rm{dose}}}_{{\rm{Fe}}}^{i}}{{{\rm{Fe}}}_{{\rm{tinf}}}}\cdot ({\rm{Hv}}(t-{\tilde{t}}_{i})-{\rm{Hv}}(t-{\tilde{t}}_{i}-{{\rm{Fe}}}_{{\rm{tinf}}})),$$

where Hv is the Heaviside-function $${\rm{Hv}}=\{\begin{array}{lll}0 & : & x\mathrm{ < 0}\\ 1 & : & x\ge 0\end{array}$$, and ${\tilde{t}}_{i}$ are the time points of injections, where ${{\rm{dose}}}_{{\rm{Fe}}}^{i}$ denote the bioavailable fraction of the injected amounts of iron. The duration of an injection (${{\rm{Fe}}}_{{\rm{tinf}}}$) is set to 15 minutes.

The first two ways of iron uptake are via enterocytes and are under control of ferroportin. The absorption of iron by enterocytes is assumed to be time delayed, modelled by a delay compartment with delay parameter ${{\rm{Delay}}}_{{\rm{intest}}}$.

44 $$\begin{array}{rcl}\frac{d{C}_{{\rm{Fe}}}(t)}{dt} & = & {C}_{{\rm{in}}}(t)-{{\rm{Delay}}}_{{\rm{intest}}}\cdot {C}_{{\rm{Fe}}}(t)\\ {C}_{{\rm{Fe}}}\mathrm{(0)} & = & 0\end{array}$$

The efflux ${{\rm{Delay}}}_{{\rm{intest}}}\cdot {C}_{{\rm{Fe}}}(t)$ of this compartment enters the compartment of enterocytes.

Enterocytes have a short lifespan (decay rate ${d}_{{\rm{entero}}}$) and the iron that was not absorbed during this time is lost^36^. Hepcidin and ferroportin regulate the iron absorption from the intestinal compartment ${F}_{{\rm{entero}}}$ into circulation. Equation 4.5 describes the resulting iron content of enterocytes.

45 $$\frac{d{F}_{{\rm{entero}}}(t)}{dt}=\frac{{k}_{{\rm{intest}}}\cdot {{\rm{Fe}}}_{{\rm{oral}}\_{\rm{in}}}(t)}{{{\rm{Fe}}}_{{\rm{intest}}}^{{\rm{\max }}}+{{\rm{Fe}}}_{{\rm{oral}}\_{\rm{in}}}(t)}-({d}_{{\rm{entero}}}+{\rm{Zferro}}(t))\cdot {F}_{{\rm{entero}}}(t)$$

The first term describes the iron influx due to oral medication or diet. In steady state, a constant amount of ${{\rm{FeDiet}}}_{{\rm{nor}}}\cdot \mathrm{(1}+{d}_{{\rm{entero}}}+{{\rm{Zferro}}}_{{\rm{nor}}})$ is absorbed from diet. Thus, in case of oral iron medication, the absorbed amount is ${{\rm{FeDiet}}}_{{\rm{nor}}}\cdot \mathrm{(1}+{d}_{{\rm{entero}}}+{{\rm{Zferro}}}_{{\rm{nor}}})+\frac{{{\rm{Fe}}}_{{\rm{oral}}\_{\rm{med}}}(t)}{{{\rm{Zferro}}}_{{\rm{\min }}}}$

**Figure 7:**
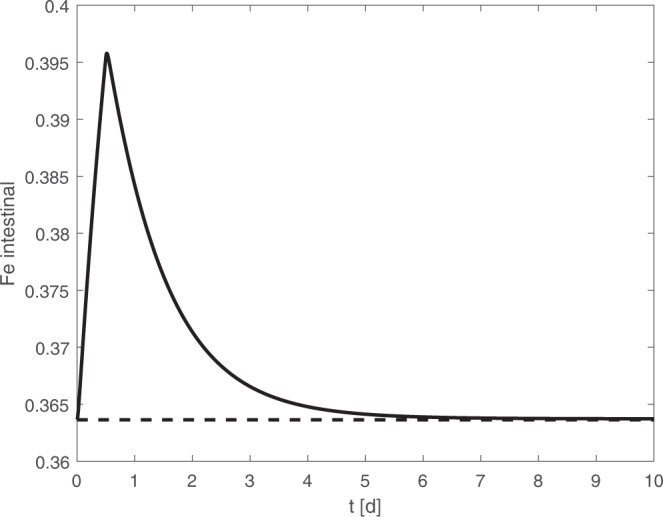
Iron content of enterocytes during iron medication. Iron from medication is absorbed with delay. The rapid decay of enterocytes results in a quick loss of parts of the administered amount of iron.

Figure 7 shows the dynamics of iron content of enterocytes after iron medication.

#### Plasma iron content

To describe the plasma iron content ${F}_{P}$, we consider non-transferrin bound iron and transferrin bound iron^36^. Intestinal absorption or intravenously applied iron, release from storage compartment, reflux from iron loaded transferrin and the haemoglobin recycling replenish the compartment^26^.

In detail, an amount of ${F}_{S}\cdot {\rm{Zferro}}$ is released from storage, and ${k}_{HB}\cdot {F}_{HB}\cdot {\rm{Zferro}}$ describes the iron reflux from the recycling system into the plasma. Iron is bound to transferrin $({k}_{ul}\cdot \frac{{\rm{TRFu}}\times {\rm{NTBI}}}{{{\rm{k}}}_{{\rm{MM}}}+{\rm{NTBI}}})$, and detached from transferrin (${k}_{lu}\cdot {{\rm{k}}}_{{\rm{FeTRF}}}\times {\rm{TRFl}}$). Iron is transferred into storage with rate ${k}_{S}$. Thus, we can describe non-transferrin bound iron NTBI in plasma by the following equation:

46 $$\begin{array}{rcl}\frac{dNTBI}{dt} & = & ({F}_{{\rm{entero}}}+{k}_{S}\cdot {F}_{S}+{k}_{{\rm{HB}}}\cdot {F}_{{\rm{HB}}})\cdot {\rm{Zferro}}+{{\rm{Fe}}}^{{\rm{inj}}}\\  &  & +\,{k}_{lu}\cdot {k}_{{\rm{FeTRF}}}\cdot {\rm{TRFl}}-{k}_{S}\cdot {\rm{NTBI}}-{k}_{ul}\cdot \frac{{\rm{TRFu}}\cdot {\rm{NTBI}}}{{k}_{{\rm{MM}}}+{\rm{NTBI}}}\\ {k}_{FeTRF} & = & \frac{-({{\rm{FeDiet}}}_{{\rm{nor}}}+{k}_{S}\cdot {F}_{{\rm{S}}0}+{k}_{{\rm{HB}}}\cdot {F}_{{\rm{HB}}0})\cdot {{\rm{Zferro}}}_{0}+{k}_{S}\cdot NTB{I}_{0}+{k}_{ul}\cdot \frac{{{\rm{TRFu}}}_{0}\cdot {{\rm{NTBI}}}_{0}}{{k}_{{\rm{MM}}}+{{\rm{NTBI}}}_{0}}}{{k}_{lu}\cdot {{\rm{TRFl}}}_{0}}\end{array}$$

#### Iron storage

Ferritin bound iron can be stored in different tissues, where the parenchymal cells of the liver are the main storage^26^. When required, iron can be released from this reservoir. In our model, the compartment *F*_S_ describes this iron storage. Iron from plasma enters the compartment ${F}_{S}$ with rate ${k}_{S}$, and, dependent on ferroportin, an amount of ${k}_{S}\cdot {\rm{Zferro}}\cdot {F}_{S}$ returns to the plasma. For the sake of parsimony, iron exchange between liver and blood is modelled via NTBI rather than TRF^40^.

47 $$\frac{d{F}_{S}}{dt}=-{k}_{S}\cdot {\rm{Zferro}}\cdot {F}_{{\rm{S}}}+{k}_{S}\cdot {\rm{NTBI}}$$

We assume that measured serum ferritin is correlated with the normalised content of compartment ${F}_{S}$ ^36^.

#### Transferrin

Transferrin delivers iron from the plasma into the bone marrow. We distinguish between loaded transferrin TRFl and unloaded transferrin ${\rm{TRFu}}$. For simplification, we do not distinguish between single and double loaded transferrin. Free transferrin receptors were occupied by available iron $({k}_{ul}\cdot \frac{{\rm{TRFu}}\cdot {\rm{NTBI}}}{{k}_{{\rm{MM}}}+{\rm{NTBI}}})$, and iron is detached from transferrin with rate ${k}_{lu}$. A total of $({k}_{{\rm{PEB}}}+{d}_{{\rm{TRFl}}})\cdot {{\rm{PEB}}}_{{\rm{out}}}\cdot {{\rm{TRFl}}}^{\alpha }$ is transferred to erythropoietic bone marrow cells for haemoglobin synthesis, where ${{\rm{PEB}}}_{{\rm{out}}}$ is the normalised efflux from the PEB (proliferating erythroblasts) compartment. With rate ${d}_{{\rm{TRFl}}}$, transferrin is degraded during this process. The rate ${k}_{{\rm{PEB}}}$ is recycled. Thus, we have the following equations.

48 $$\frac{d{\rm{TRFl}}}{dt}={k}_{ul}\cdot \frac{{\rm{TRFu}}\cdot {\rm{NTBI}}}{{k}_{{\rm{MM}}}+{\rm{NTBI}}}-{k}_{lu}\cdot {\rm{TRFl}}-({k}_{{\rm{PEB}}}+{d}_{{\rm{TRFl}}})\cdot {{\rm{PEB}}}_{{\rm{out}}}\cdot {{\rm{TRFl}}}^{\alpha }$$

49 $$\begin{array}{rcc}\frac{d{\rm{TRFu}}}{dt} & = & -{k}_{ul}\cdot \frac{{\rm{TRFu}}\cdot {\rm{NTBI}}}{{k}_{{\rm{MM}}}+{\rm{NTBI}}}+{k}_{lu}\cdot {\rm{TRFl}}+{k}_{{\rm{PEB}}}\cdot {{\rm{PEB}}}_{{\rm{out}}}\cdot {{\rm{TRFl}}}^{\alpha }\\  &  & +\,{k}_{{\rm{TRFu}}}\cdot {{\rm{PEB}}}_{{\rm{out}}}\cdot {Z}_{{\rm{TRF}}}-{d}_{{\rm{TRFu}}}\cdot {\rm{TRFu}}\\ {k}_{lu} & = & \frac{{k}_{ul}\cdot \frac{{{\rm{TRFu}}}_{0}\cdot {{\rm{NTBI}}}_{0}}{{k}_{{\rm{MM}}}+{{\rm{NTBI}}}_{0}}-({k}_{{\rm{PEB}}}+{d}_{{\rm{TRFl}}})\cdot {{{\rm{TRFl}}}_{0}}^{\alpha }}{{{\rm{TRFl}}}_{0}}\end{array}$$

$$\begin{array}{rcl}{d}_{{\rm{TRFu}}} & = & (-{k}_{ul}\cdot \frac{{{\rm{TRFu}}}_{0}\cdot {{\rm{NTBI}}}_{0}}{{k}_{{\rm{MM}}}+{{\rm{NTBI}}}_{0}}+{k}_{lu}\cdot {{\rm{TRFl}}}_{0}\\  &  & +\,{k}_{{\rm{PEB}}}\cdot {{\rm{TRFl}}}_{0}^{\alpha }+{k}_{{\rm{TRFu}}}\cdot {Z}_{{\rm{TRF}}}\mathrm{(0)})/{{\rm{TRFu}}}_{0}\end{array}$$

Increased levels of circulating transferrin were observed under hypoxia^39^. In our erythropoiesis model, hypoxia is assumed to increase the production of endogenous EPO resulting in an increased production of red blood cells in the bone marrow. To model this issue phenomenologically, we assume a positive correlation of ${{\rm{P}}{\rm{E}}{\rm{B}}}_{{\rm{o}}{\rm{u}}{\rm{t}}}$ and transferrin production. Moreover, transferrin synthesis in the liver increases with iron deficiency^39^. Therefore, we consider plasma and storage iron and the haemoglobin level as negatively correlated. Moreover, inflammatory processes can down-regulate the transferrin level^39^. Thus hepcidin is also considered negatively correlated with transferrin production. All negative correlations are weighted and summarized in the term ${{\rm{Z}}}_{{\rm{T}}{\rm{R}}{\rm{F}}}$.

410 $${{\rm{Z}}}_{{\rm{TRF}}}={{\rm{ZTRF}}}_{{\rm{\max }}}-({{\rm{ZTRF}}}_{{\rm{\max }}}-{{\rm{ZTRF}}}_{{\rm{\min }}})\cdot \exp (-\,\log \left(\frac{{{\rm{ZTRF}}}_{{\rm{\max }}}-{{\rm{ZTRF}}}_{{\rm{\min }}}}{{{\rm{ZTRF}}}_{{\rm{\max }}}-{{\rm{ZTRF}}}_{{\rm{nor}}}}\right)\cdot $$

$${\left(\frac{{{\rm{k}}}_{{\rm{TRFFP}}}\cdot {F}_{P}+{k}_{{\rm{TRFHB}}}\cdot {\rm{HB}}/{{\rm{HB}}}^{{\rm{nor}}}+{k}_{{\rm{TRFFS}}}\cdot {F}_{S}+{k}_{{\rm{TRFHEP}}}\cdot {\rm{HEP}}}{{k}_{{\rm{TRFFP}}}+{k}_{{\rm{TRFHB}}}+{k}_{{\rm{TRFFS}}}+{k}_{{\rm{TRFHEP}}}}\right)}^{ZTR{F}_{b}})$$

After iron transfer, transferrin is degraded in the cell with rate ${d}_{{\rm{TRFl}}}$. Unloaded transferrin is degraded with rate ${d}_{{\rm{TRFu}}}$.

We calculate transferrin and transferrin saturation by

4.11 $${\rm{TRF}}={\rm{TRFu}}+{\rm{TRFl}}$$

4.12 $${\rm{TSAT}}=\frac{{\rm{TRFl}}}{{\rm{TRFu}}+{{\rm{TRF}}}_{{\rm{l}}}}$$

#### Iron in Erythropoietic Cells

Proliferating erythrocytic blasts in the bone marrow are assumed to absorb iron from plasma for haemoglobin synthesis^26^. In our model, iron uptake is assumed for cells leaving the PEB compartment. The absorbed amount of iron

$$({k}_{{\rm{PEB}}}+{d}_{{\rm{TRFl}}})\cdot {{\rm{PEB}}}_{{\rm{out}}}\cdot {{\rm{TRFl}}}^{\alpha }$$

passes through the compartments MEB, RET and ERY which is modelled by the same equations and regulations than the corresponding cell numbers (^1,2^, supplemental file 1). The effluxes of the ERY compartments correspond to dying erythrocytes which are subjected to iron recycling. Likewise, the effluxes of the parallel modelled iron compartments serve as influx into the iron recycling compartment. We assume a loss of ${d}_{Fe}$ during this process which can be interpreted as a certain percentage of non-recycled erythrocytes (e.g. due to blood losses). An iron amount of

$$\mathrm{(1}-{d}_{{\rm{Fe}}})\cdot \frac{{{\rm{Fe}}}_{{\rm{ERY}}}^{{\rm{out}}}}{{{\rm{Fe}}}_{{\rm{ERY}}}^{{\rm{out}}\_{\rm{nor}}}}$$

transfers into the recycling compartment *F*_HB_. The iron content of erythrocytes is used to calculate haemoglobin levels. At this, contributions of reticulocytes are neglected. In case of bleeding or phlebotomy, the reticulocyte compartment, the 15 ageing compartments of erythrocytes and the random ageing compartments are reduced by the same proportion with respect to cell count and iron content^21^.

#### Recycling system

The recycling compartment *F*_HB_ consists of macrophages, which recycle iron from haemoglobin after the end of the lifespan of erythrocytes^26,36,41^. The recycled amount depends on ferroportin^41^. Here, $\mathrm{(1}-{d}_{Fe})\cdot \frac{{{\rm{Fe}}}_{{\rm{ERY}}}^{{\rm{out}}}}{{{\rm{Fe}}}_{{\rm{ERY}}}^{{\rm{out}}\_{\rm{nor}}}}$ is the influx of haemoglobin bound iron into the recycling compartment, and a recycled amount of ${k}_{{\rm{HB}}}\cdot {F}_{{\rm{HB}}}\cdot {\rm{Zferro}}$ re-enters the plasma.

413 $$\frac{d{F}_{{\rm{HB}}}}{dt}=\mathrm{(1}-{d}_{{\rm{Fe}}})\cdot \frac{{{\rm{Fe}}}_{{\rm{ERY}}}^{{\rm{out}}}}{{{\rm{Fe}}}_{{\rm{ERY}}}^{{\rm{out}}\_{\rm{nor}}}}-{k}_{{\rm{HB}}}\cdot {F}_{{\rm{HB}}}\cdot {\rm{Zferro}}$$

$${k}_{{\rm{HB}}}=\frac{1-{d}_{{\rm{Fe}}}}{{F}_{{\rm{HB}}0}\cdot {{\rm{Zferro}}}_{0}}$$

### Numerical methods for simulation

Implementation and simulations of the model were performed using MATLAB 7.5.0.342 (R2007b) with SIMULINK toolbox (The MathWorks Inc., Natick, MA, USA). Numerical integrations of the differential equation system were performed using the variable step solver from Adams and Bashford included in the SIMULINK toolbox (ode113). A small application of the model for 3 scenarios is placed at the Hemato-models platform at Leipzig Health Atlas LHA (https://apps.lha.test.life.uni-leipzig.local/hemato-models/ and https://www.health-atlas.de/models/27, the description can be downloaded from https://www.health-atlas.de/documents/13). Additional we provide the code as Rcpp application in Supplemental file 5.

### Model parameters

Most parameters of the erythropoiesis model are retained from^1^. However, incorporation of the iron metabolism model required a few parameter adaptations and new parameters. These parameters were estimated by comparing model predictions with time series data after treatment with EPO or iron in different scenarios. Fitting is achieved by comparing model prediction and data via the condition

414 $${\int }_{{t}_{0}}^{{t}_{1}}|\,\log ({f}_{{\rm{model}}}(t,{\bf{k}}))-\,\log ({f}_{{\rm{data}}}(t))|dt\to \mathop{min}\limits_{k},$$

where ${f}_{{\rm{model}}}(t,{\bf{k}})$ is the solution of the differential equation system at time *t* for the parameter set ${\bf{k}}={k}_{1},\,\ldots {k}_{n}$. Here, ${t}_{0}\le t\le {t}_{1}$ is the time range with available data. The interpolated time course of patients data is represented by ${f}_{{\rm{data}}}(t)$. In the following, the left hand side of Eq. (4.14) is referred as the *fitness function*. If several data sets were fitted simultaneously, the corresponding fitness functions were added. To find optimal parameter settings, we applied (1 + 3)-evolutionary-strategies with self-adapting mutation step size^42,43^. These non-deterministic algorithms use principles of evolution such as mutation, realisation and survival of the fittest to optimise the agreement of model and data by an appropriate choice of parameters. (1 + 3) is a strategy with one possibly immortal parent parameter set having three children parameter sets in every generation (see^42,43^ for details). A sensitivity analysis of model parameters is provided in the supplemental file 3.

### Data sets

Available literature data sets comprise time course data of ferritin, transferrin saturation, haemoglobin, haematocrit, reticulocyte counts, percentage of reticulocytes, red blood cell counts or serum concentration of EPO after EPO or iron application. The automated tool “ycasd”^44,45^ was used to extract the data from published figures as precisely as possible.

Haemoglobin median and percentiles of CHOP-Chemotherapy patients were obtained from studies of the German High-Grade Non-Hodgkin’s Lymphoma Study Group. All patients had given informed consent and studies were approved by Ethics Committee at the Saarland Medical Association and were carried out in accordance with the principles of good clinical practice and the declaration of Helsinki. Details on ethics committees and reference numbers can be found in the respective publications of the studies^11,15^. For our modelling, we use data of 174 CHOP14-chemotherapy patients who did not receive erythrocyte concentrates. The list of datasets used for modelling is shown in Table 2. The data sets are provided in supplemental file 4.

**Table 2:** Data sets used for model development.

Population	Intervention	Source
healthy male athletes	200 IU/kg EPO sc (d 0, 2, 4, 7, 10) without iron medication	^8^
healthy male subjects	300 IU/kg EPO sc (d 0, 3, 6, 9) 300 mg oral iron (d 0–9)	^9^
healthy male subjects	400 IU/kg EPO sc (d 0, 4, 8) 300 mg oral iron (d 0–9)	^9^
healthy male subjects	600 IU/kg EPO sc (d 0, 9) 300 mg oral iron (d 0–9)	^9^
blood donors	donation of 500 ml blood without iron medication	^10^
blood donors	donation of 500 ml blood with oral ferrous gluconate, 325 mg daily	^10^
lymphoma patients	CHOP chemotherapy	^11,15^
healthy subjects	100 IU EPO iv	^46^

### Ethics statement

Ethics approval and consent to participate: Data were obtained from studies of the German High-Grade Non-Hodgkin’s Lymphoma Study Group. All patients had given informed consent and studies were approved by Ethics Committee at the Saarland Medical Association and were carried out in accordance with the principles of good clinical practice and the declaration of Helsinki. Details on ethics committees and reference numbers can be found in the respective publications of the studies^11,15^.

## Supplementary information

A biomathematical model of human erythropoiesis and iron metabolism: model equations and parameters.

A biomathematical model of human erythropoiesis and iron metabolism: supplemental figures.

A biomathematical model of human erythropoiesis and iron metabolism: sensitivity analysis.

A biomathematical model of human erythropoiesis and iron metabolism: data sets.

A biomathematical model of human erythropoiesis and iron metabolism: Simulation Model.
