# Supplementary material for: A biomathematical model of human erythropoiesis and iron metabolism
Source: Sci Rep. 2020 May 25;10:8602. doi: 10.1038/s41598-020-65313-5 (PMC7248076; doi:10.1038/s41598-020-65313-5)
Supplement: Supplementary file 1 — A biomathematical model of human erythropoiesis and iron metabolism: model equations and parameters. [file 41598_2020_65313_MOESM1_ESM.pdf]

A biomathematical model of human erythropoiesis and iron metabolism: model equations and parameters

Sibylle Schirm[1] and Markus Scholz[1,*]

1 Institute for Medical Informatics, Statistics and Epidemiology, University of Leipzig, Leipzig, Germany

* markus.scholz@imise.uni-leipzig.de

S1 Model Equations and Parameters

Here we present compartments and equations of the cell kinetic model required to model iron metabolism. Equations of the other compartments of the cell kinetic model can be found elsewhere [1,2]. An overview of model compartments can be found in table S1.

Table S1: variables and model compartments

quantity	meaning
C_X	content of compartment X
C_X^{nor}	content of compartment X in steady state
C_X^{rel}	content of compartments X relative to steady state
C_X^{in}	influx in compartment X
$C_X^{in\_nor}$	normal influx
C_X^{out}	efflux from compartment X
$C_X^{out\_nor}$	normal efflux
a_X	proliferative fraction in cell compartment X
A_X	amplification in cell compartment X
A_X^{in}	amplification of influx
A_X^{out}	amplification of efflux
n_X	average number of mitoses, $n_X = \text{ld } A_X$
p	self-renewal probability of stem cells
τ_X	average duration of cell cycle
T_X	average transit time of active cells, $T_X = n_X \tau_X$
T_X^t	total transit time
Y^{min}	quantity Y under minimum stimulation
Y^{nor}	quantity Y in steady state
Y^{int}	quantity Y under intensified stimulation
Y^{max}	quantify Y under maximum stimulation
b_Y	sensitivity of Y under stimulation
S	stem cells
BE	burst forming unit erythorid
CE	colony forming unit erythroid
PEB	proliferating erythrocytic blasts
MEB	maturing erythrocytic blasts
RET	reticulocytes
ERY	erythrocytes
EPO	erythropoietin

See also [1,2] for details.

S1.1 Additional Cell Kinetic Model Equations

We use the same compartment structure as in [2] to model iron content of the erythropoietic cell lineage. The efflux of the PEB compartment affects the iron flux into the compartment MEB. The iron content of MEB, RET or ERY is denoted as FeMEB, FeRET, and FeERY, respectively. In analogy to cell maturation, iron transfer is modelled in dependence on internalised EPO using the same parameter settings as for the cell maturation.

Compartment PEB

$$\begin{aligned}
A_{\text{PEB}} &= Z(C_{EPO}^{\text{rel}}) \\
\frac{d}{dt}C_{\text{PEB}} &= C_{\text{CE}}^{\text{out}} A_{\text{PEB}}^{\text{in}} - \frac{C_{\text{PEB}}}{T_{\text{PEB}}} - \Psi_{\text{PEB}} \cdot C_{\text{PEB}} \\
C_{\text{PEB}}^{\text{out}} &= \frac{C_{\text{PEB}} A_{\text{PEB}}^{\text{out}}}{T_{\text{PEB}}} \\
C_{\text{PEB}}(0) &= C_{\text{PEB}}^{\text{nor}} = C_{\text{CE}}^{\text{out_nor}} A_{\text{PEB}}^{\text{in_nor}} \cdot T_{\text{PEB}}^{\text{nor}} \\
C_{\text{PEB}}^{\text{out}}(0) &= C_{\text{PEB}}^{\text{out_nor}} = C_{\text{CE}}^{\text{out_nor}} A_{\text{PEB}}^{\text{nor}}.
\end{aligned}$$

Ψ_{PEB} denotes the effect of chemotherapy on PEB.

Compartment FeMEB: Iron in Maturing Erythrocytic Blasts

Dependent on the efflux of the compartment PEB ($C_{\text{PEB}}^{\text{out}}$), iron is absorbed into maturing erythrocytic blasts:

$$(k_{\text{PEB}} + d_{\text{TRFl}}) \cdot \text{PEB}_{\text{out}} \cdot \text{TRFl}^{\alpha}.$$

In analogy to [2], maturation is modelled by splitting FeMEB into $N_{\text{FeMEB}} = 15$ subcompartments. Chemotherapy effects on MEB are neglected.

$$\begin{aligned}
T_{\text{FeMEB}} &= Z(C_{EPO}^{\text{rel}}) \\
C_{\text{FeMEB}} &= \sum_{i=1}^{N_{\text{FeMEB}}} C_{\text{FeMEB}_i} \\
\frac{d}{dt}C_{\text{FeMEB}_1} &= C_{\text{FeMEB}}^{\text{in}} - C_{\text{FeMEB}_1}\frac{N_{\text{FeMEB}}}{T_{\text{FeMEB}}} \\
\frac{d}{dt}C_{\text{FeMEB}_i} &= C_{\text{FeMEB}_{i-1}}^{\text{out}} - C_{\text{FeMEB}_i}\frac{N_{\text{FeMEB}}}{T_{\text{FeMEB}}}, \quad i = 2, \ldots, N_{\text{FeMEB}} \\
C_{\text{FeMEB}_i}^{\text{out}} &= C_{\text{FeMEB}_i}\frac{N_{\text{FeMEB}}}{T_{\text{FeMEB}}}, \quad i = 1, \ldots, N_{\text{FeMEB}} \\
C_{\text{FeMEB}}^{\text{out}} &= C_{\text{FeMEB}_{N_{\text{FeMEB}}}}^{\text{out}},
\end{aligned}$$

with the initial values

$$\begin{aligned}
C_{\text{FeMEB}}(0) &= C_{\text{FeMEB}}^{\text{nor}} = (k_{\text{PEB}} + d_{\text{TRFl}}) \cdot C_{\text{PEB}}^{\text{out_nor}} \cdot \text{TRF}_{\text{lnor}}{}^{\alpha} T_{\text{FeMEB}}^{\text{nor}} \\
C_{\text{FeMEB}_i}(0) &= C_{\text{FeMEB}_i}^{\text{nor}} = (k_{\text{PEB}} + d_{\text{TRFl}}) \cdot C_{\text{PEB}}^{\text{out_nor}} \cdot \text{TRF}_{\text{lnor}}{}^{\alpha} \frac{T_{\text{FeMEB}}^{\text{nor}}}{N_{\text{FeMEB}}}, \quad i = 1, \ldots, N_{\text{FeMEB}} \\
C_{\text{FeMEB}_i}^{\text{out}}(0) &= C_{\text{FeMEB}_i}^{\text{out_nor}} = C_{\text{FeMEB}_i}^{\text{nor}} \frac{N_{\text{FeMEB}}}{T_{\text{FeMEB}}^{\text{nor}}} = (k_{\text{PEB}} + d_{\text{TRFl}}) \cdot C_{\text{PEB}}^{\text{out_nor}} \cdot \text{TRF}_{\text{lnor}}{}^{\alpha}, \quad i = 1, \ldots, N_{\text{FeMEB}} \\
C_{\text{FeMEB}}^{\text{out}}(0) &= C_{\text{FeMEB}}^{\text{out_nor}} = C_{\text{FeMEB}_{N_{\text{FeMEB}}}}^{\text{out_nor}} = (k_{\text{PEB}} + d_{\text{TRFl}}) \cdot C_{\text{PEB}}^{\text{out_nor}} \cdot \text{TRF}_{\text{lnor}}{}^{\alpha}.
\end{aligned}$$

Compartment FeRET: Iron in Reticulocytes

$$
\begin{aligned}
T_{\text{FeRET}} &= T^{\text{nor}}_{\text{FeMEB}} + T^{\text{nor}}_{\text{FeRET}} - T_{\text{FeMEB}} \\
\frac{d}{dt} C_{\text{FeRET}} &= C^{\text{out}}_{\text{FeMEB}} - \frac{C_{\text{FeRET}}}{T_{\text{FeRET}}} \\
C^{\text{out}}_{\text{FeRET}} &= \frac{C_{\text{FeRET}}}{T_{\text{FeRET}}} \\
C_{\text{FeRET}}(0) &= C^{\text{nor}}_{\text{FeRET}} = C^{\text{nor}}_{\text{FeERY}} \frac{q_{\text{FeRET}}}{1 - q_{\text{FeRET}}} \\
C^{\text{out}}_{\text{FeRET}}(0) &= C^{\text{out\_nor}}_{\text{FeRET}} = C^{\text{out\_nor}}_{\text{FeMEB}} \\
T^{\text{nor}}_{\text{FeRET}} &= \frac{C^{\text{nor}}_{\text{FeRET}}}{C^{\text{out\_nor}}_{\text{FeRET}}} = \frac{q_{\text{FeRET}}}{1 - q_{\text{FeRET}}} \left((1 - s^{\text{nor}}_{\text{FeERY}}) T_{\text{FeERY\_rnd}} + s^{\text{nor}}_{\text{FeERY}} T_{\text{FeERY\_age}} \right).
\end{aligned}
$$

q_{FeRET} is the ratio of reticulocytes to the total number of red blood cells in steady state. $s^{\text{nor}}_{\text{FeERY}}$, $T_{\text{FeERY\_rnd}}$, and $T_{\text{FeERY\_age}}$ are explained in the next section.

Compartment FeERY: Iron in Erythrocytes

In analogy to [2], the compartment FeERY consists of the subcompartments ”RANDOM” and ”AGE”, corresponding to different mechanisms of erythrocyte degradation. In steady state, most erythrocytes are removed dependent on age and the contained iron enters the recycling compartment. Aging is modelled by division of the FeERY compartment into subcompartments. With EPO medication, erythrocytes are degraded more randomly (see [3]). Therefore, the fluxes into the subcompartments RANDOM and AGE are regulated by the factor s_{FeERY}, which depends on the bone marrow output of the reticulocytes. $T_{\text{FeERY\_rnd}}$, and $T_{\text{FeERY\_age}}$ are the transition times of these compartments [1–5].

$$
\begin{aligned}
s_{\text{FeERY}} &= \exp\left(\left(\frac{C^{\text{out}}_{\text{FeRET}}}{C^{\text{out\_nor}}_{\text{FeRET}}} \right)^2 \ln s^{\text{nor}}_{\text{FeERY}} \right) \\
C_{\text{FeERY}} &= C_{\text{FeERY\_age}} + C_{\text{FeERY\_rnd}} \\
C_{\text{FeERY\_age}} &= \sum_{i=1}^{N_{\text{FeERY}}} C_{\text{FeERY\_age\_}i} \\
\frac{d}{dt} C_{\text{FeERY\_age\_1}} &= s_{\text{FeERY}} C^{\text{out}}_{\text{FeRET}} - C_{\text{FeERY\_age\_1}} \frac{N_{\text{FeERY}}}{T_{\text{FeERY\_age}}} \\
\frac{d}{dt} C_{\text{FeERY\_age\_}i} &= C^{\text{out}}_{\text{FeERY\_age\_}(i-1)} - C^{\text{out}}_{\text{FeERY\_age\_}i}, \quad i = 2, \ldots, N_{\text{FeERY}} \\
C^{\text{out}}_{\text{FeERY\_age\_}i} &= C_{\text{FeERY\_age\_}i} \frac{N_{\text{FeERY}}}{T_{\text{FeERY\_age}}} \\
\frac{d}{dt} C_{\text{FeERY\_rnd}} &= (1 - s_{\text{FeERY}}) C^{\text{out}}_{\text{FeRET}} - C_{\text{FeERY\_rnd}} \frac{1}{T_{\text{FeERY\_rnd}}},
\end{aligned}
$$

with initial conditions

$$
\begin{aligned}
C_{\text{FeERY}}(0) &= C^{\text{nor}}_{\text{FeERY}} = C^{\text{nor}}_{\text{FeERY\_age}} + C^{\text{nor}}_{\text{FeERY\_rnd}} \\
C_{\text{FeERY\_age}}(0) &= C^{\text{nor}}_{\text{FeERY\_age}} = \sum_{i=1}^{N_{\text{FeERY}}} C^{\text{nor}}_{\text{FeERY\_age\_}i} = s^{\text{nor}}_{\text{FeERY}} C^{\text{out\_nor}}_{\text{FeRET}} T_{\text{FeERY\_age}} \\
C_{\text{FeERY\_age\_1}}(0) &= C^{\text{nor}}_{\text{FeERY\_age\_1}} = s^{\text{nor}}_{\text{FeERY}} C^{\text{out\_nor}}_{\text{FeRET}} \frac{T_{\text{FeERY\_age}}}{N_{\text{FeERY}}}
\end{aligned}
$$

$$C_{\text{FeERY\_age\_}i}(0) = C^{\text{nor}}_{\text{FeERY\_age\_}i} = C^{\text{out\_nor}}_{\text{FeERY\_age\_}i-1}\frac{T_{\text{FeERY\_age}}}{N_{\text{FeERY}}}, \quad i = 2, \ldots, N_{\text{FeERY}}$$

$$= s^{\text{nor}}_{\text{FeERY}} C^{\text{out\_nor}}_{\text{FeRET}} \frac{T_{\text{FeERY\_age}}}{N_{\text{FeERY}}}, \quad i = 1, \ldots, N_{\text{FeERY}}$$

$$C^{\text{out}}_{\text{FeERY\_age\_}i}(0) = C^{\text{out\_nor}}_{\text{FeERY\_age\_}i} = C^{\text{nor}}_{\text{FeERY\_age\_}i}\frac{N_{\text{FeERY}}}{T_{\text{FeERY\_age}}} = s^{\text{nor}}_{\text{FeERY}} C^{\text{out\_nor}}_{\text{FeRET}}$$

$$C_{\text{FeERY}_{\text{rnd}}}(0) = C^{\text{nor}}_{\text{FeERY\_rnd}} = (1 - s^{\text{nor}}_{\text{FeERY}}) C^{\text{out\_nor}}_{\text{FeRET}} T_{\text{FeERY}_{\text{rnd}}}$$

Endogenous production of EPO

The endogenous production of EPO (EPO_{prod}) is assumed to be dependent on the tissue oxygen tension in the kidneys and the haemoglobin content of erythrocytes.

Table S2: Variables for endogenous EPO production from [5, 6]

quantity	meaning	type/calculation
$P^t_{O_2}$	tissue oxygen tension in kidneys	function of time
$P^{t\_\text{nor}}_{O_2}$	normal value of tissue oxygen tension in kidneys	
$S^t_{O_2}$	tissue saturation of oxygen	function of time
$S^{t\_\text{nor}}_{O_2}$	normal tissue saturation of oxygen	constant
P_{50}	partial oxygen pressure corresponding to $S^t_{O_2} = 50\%$	26.5 mm Hg
$P^{A\_\text{nor}}_{O_2}$	arterial oxygen tension, normal value	97 mm Hg
ΔSO_2	desaturation of HB (arteriovenous difference), normal value	20 %
γ	Hill coefficient	2.65
P^{endo}_{max}	maximum EPO production	200 (set)
b_{EPO}	sensitivity of EPO production to changes in $P^t_{O_2}$	$\ln 200$ (set)

$$\begin{aligned}
P^t_{O_2} &= P_{50} \cdot \left(\frac{S^t_{O_2}}{100 - S^t_{O_2}}\right)^{\frac{1}{\gamma}} \\
S^t_{O_2} &= \frac{100}{\left(\frac{P_{50}}{P^{A_\text{nor}}_{O_2}}\right)^{\gamma} + 1} - \Delta SO_2 \cdot \frac{\text{HB}^{\text{nor}}}{\text{HB}} \\
S^{t_\text{nor}}_{O_2} &= \frac{100}{\left(\frac{P_{50}}{P^{A_\text{nor}}_{O_2}}\right)^{\gamma} + 1} - \Delta SO_2 \\
P^{t_\text{nor}}_{O_2} &= P_{50} \cdot \left(\frac{S^{t_\text{nor}}_{O_2}}{100 - S^{t_\text{nor}}_{O_2}}\right)^{\frac{1}{\gamma}} \\
f &= \frac{P^t_{O_2}}{P^{t_\text{nor}}_{O_2}} \\
\text{EPO}_{\text{prod}} &= P^{\text{endo}}_{max} \cdot e^{-b_{\text{EPO}} \cdot f} \qquad [5] \\
\text{EPO}_{\text{prod}}(0) &= 1.
\end{aligned}$$

S1.2 Parameters of the Erythropoiesis Model

We here present all parameter settings of the model.

Table S3: **Parameter values (1).**

parameter	value	
q_{RET}	1.60E-02	set
$T_{\mathrm{ERY}}^{\mathrm{rnd}}$	1.02E+03	[7]
$T_{\mathrm{ERY}}^{\mathrm{age}}$	3.06E+03	[7]
$s_{\mathrm{ERY}}^{\mathrm{nor}}$	9.00E-01	[5], p. 40
N_{ERY}	1.00E+01	[5], p. 41
$\mathrm{HK}^{\mathrm{nor}}$	4.30E-01	set
$\mathrm{ERY}^{\mathrm{nor}}$	4.50E+00	set
$\mathrm{RET}^{\mathrm{nor}}$	1.00E+02	set
$\mathrm{RET\%}^{\mathrm{nor}}$	9.50E+00	set
$\mathrm{HB}^{\mathrm{nor}}$	1.35E+01	set
P_{50}	2.65E+01	set
$\overline{P_{O_2}^{A}}$	9.70E+01	set
ΔSO_2	2.00E+01	set
γ	2.65E+00	set
P_{max}^{endo}	2.00E+02	[5]
b_{EPO}	5.30E+00	fitted
$\mathrm{EPO}_{\mathrm{Vc}}$	3.20E-02	[8]
$\mathrm{EPO}_{\mathrm{serum}}$	1.50E+01	[8]
VB	5.50E+00	set
k_{el}	1.60E-01	fitted
k_{pt}	6.97E-02	fitted
k_{tp}	1.07E-01	fitted
k_{on}	1.44E-02	fitted
k_{off}	3.20E+01	fitted
R_0	6.43E+01	[8]
k_{int}	2.00E+00	[8]
k_{deg}	7.43E-02	fitted
w_{RET}	5.00E-02	set
w_{MEB}	9.17E-02	fitted
w_{PEB}	2.36E-01	fitted
w_{CE}	4.33E+00	fitted
w_{BE}	6.14E-02	fitted

Table S4: **Parameter values (2).**

parameter	value	
$T_{\mathrm{BE}}^{\mathrm{min}}$	5.00E+01	fitted
$T_{\mathrm{BE}}^{\mathrm{nor}}$	4.00E+01	[7]
$T_{\mathrm{BE}}^{\mathrm{max}}$	3.50E+01	fitted
$T_{\mathrm{BE}}^{\mathrm{b}}$	2.48E+00	fitted
$A_{\mathrm{BE}}^{\mathrm{min}}$	1.60E+01	fitted
$A_{\mathrm{BE}}^{\mathrm{nor}}$	6.40E+01	[7]
$A_{\mathrm{BE}}^{\mathrm{max}}$	1.28E+02	fitted
$A_{\mathrm{BE}}^{\mathrm{b}}$	6.24E-01	fitted
$A_{\mathrm{CE}}^{\mathrm{min}}$	1.00E+00	[7]
$A_{\mathrm{CE}}^{\mathrm{nor}}$	3.20E+01	[7]
$A_{\mathrm{CE}}^{\mathrm{max}}$	1.28E+02	fitted
$A_{\mathrm{CE}}^{\mathrm{b}}$	1.08E-02	fitted
$T_{\mathrm{CE}}^{\mathrm{min}}$	1.10E+02	fitted
$T_{\mathrm{CE}}^{\mathrm{nor}}$	4.00E+01	[7]
$T_{\mathrm{CE}}^{\mathrm{max}}$	3.50E+01	fitted
$T_{\mathrm{CE}}^{\mathrm{b}}$	4.82E-01	fitted
$T_{\mathrm{PEB}}^{\mathrm{min}}$	4.81E+01	fitted
$T_{\mathrm{PEB}}^{\mathrm{nor}}$	4.80E+01	fitted
$T_{\mathrm{PEB}}^{\mathrm{max}}$	4.29E+01	fitted
$T_{\mathrm{PEB}}^{\mathrm{b}}$	1.30E+00	fitted
$A_{\mathrm{PEB}}^{\mathrm{min}}$	1.00E+00	[7]
$A_{\mathrm{PEB}}^{\mathrm{nor}}$	6.40E+01	[7]
$A_{\mathrm{PEB}}^{\mathrm{max}}$	1.28E+02	fitted
$A_{\mathrm{PEB}}^{\mathrm{b}}$	1.01E+00	fitted
$T_{\mathrm{MEB}}^{\mathrm{min}}$	6.20E+01	set
$T_{\mathrm{MEB}}^{\mathrm{nor}}$	6.00E+01	fitted
$T_{\mathrm{MEB}}^{\mathrm{max}}$	3.10E+01	fitted
$T_{\mathrm{MEB}}^{\mathrm{b}}$	3.29E-01	fitted
k_a^F	2.06E-01	fitted
k_e^F	1.19E+00	fitted
k_{Delay}^L	1.49E-01	fitted
k_a^L	2.88E-01	fitted
k_e^L	4.99E-01	fitted
k_{Delay}^F	1.13E+00	fitted
k_{FL}	1.05E+01	fitted

Table S5: **Parameter values taken from [4].**

parameter	meaning	value
S^{nor}	normal value of stem cells	1.00E+00
τ_S	duration of cell cycle	8.00E+00
p_δ	self-renewal probability	1.00E-01
a_S^{min}	proliferative fraction under minimal stimulation	1.00E-02
a_S^{nor}	proliferative fraction under normal stimulation	1.50E-01
a_S^{int}	proliferative fraction under intensified stimulation	4.50E-01
a_S^{max}	proliferative fraction under maximal stimulation	1.00E+00
α_E	weighting parameter E for regulation of a	1.50E-01
a_{BE}^{min}	proliferative fraction under minimal stimulation	3.00E-01
a_{BE}^{nor}	proliferative fraction under normal stimulation	3.30E-01
a_{BE}^{int}	proliferative fraction under intensified stimulation	6.60E-01
a_{BE}^{max}	proliferative fraction under maximal stimulation	1.00E+00
w_E	weighting parameter E for regulation of a	0.3
w_G	weighting parameter G for regulation of a	0.1
w_S	weighting parameter S for regulation of a	1
ϑ_E	weighting parameter E for regulation of p	-2
ϑ_G	weighting parameter G for regulation of p	-8

Table S6: **Toxicity parameters of CHOP chemotherapy**

parameter	meaning	value	
$f_{\mathrm{fc}}^{\mathrm{CDCHOP}}$	first cycle effect	1.11E+00	fitted
$k_{\mathrm{Delay}}^{\mathrm{CDCHOP}}$	delay of chemotherapy effect	6.35E-02	fitted
$k_{\mathrm{S}}^{\mathrm{CDCHOP}}$	stem cell toxicity	2.16E-01	fitted
$k_{\mathrm{BE}}^{\mathrm{CDCHOP}}$	toxicity effect on BE	1.91E-04	fitted
$k_{\mathrm{CE}}^{\mathrm{CDCHOP}}$	toxicity effect on CE	8.39E-02	fitted
$k_{\mathrm{PEB}}^{\mathrm{CDCHOP}}$	toxicity effect on PEB	5.57E-02	fitted
$k_{\mathrm{MEB}}^{\mathrm{CDCHOP}}$	toxicity effect on MEB	0.00E+00	set
$k_{\mathrm{RET}}^{\mathrm{CDCHOP}}$	toxicity effect on RET	0.00E+00	set

S1.3 Parameters of the iron metabolism model

Table S7: **Parameter values.**

parameter	meaning	value	
d_{Fe}	loss due to non-recycled iron	3.64E-01	set
k_{ul}	iron binding rate of transferrin	3.84E+01	fitted
k_{PEB}	iron absorption rate of erythroblasts	2.06E-01	fitted
k_{MM}	Michaelis-Menten constant of TRF iron uptake	9.88E+00	fitted
$\mathrm{TRFZ}_{\mathrm{max}}$	TRF production under high plasma iron	9.99E-01	fitted
$\mathrm{TRFZ}_{\mathrm{min}}$	TRF production under low plasma iron	1.04E+00	fitted
$\mathrm{TRFZ}_{\mathrm{nor}}$	TRF production under steady-state plasma iron	1.00E+00	set
$\mathrm{TRFZ}_{\mathrm{b}}$	Sensitivity of TRF production	7.54E+01	fitted
k_{TRFFP}	F_{P} influence on TRF production	8.94E-01	fitted
k_{TRFHB}	HB influence on TRF production	3.52E+04	fitted
k_{TRFFS}	F_{S} influence on TRF production	4.78E+01	fitted
k_{TRFHEP}	HEP influence on TRF production	4.42E+00	fitted
k_{lu}	transferrin iron detache rate	3.31E+00	set
k_{TRFu}	production of TRF in steady-state	2.09E-02	fitted
d_{TRFl}	TRFl degradation rate	1.69E-02	fitted
$\mathrm{d}_{\mathrm{TRFu}}$	TRFu degradation rate	3.97E-03	set
$\mathrm{k}_{\mathrm{FeTRF}}$	iron detachment from TRF	7.65E-01	set
$\mathrm{ZFerro}_{\mathrm{max}}$	ferroportin for high hepcidin	3.95E-02	fitted
$\mathrm{ZFerro}_{\mathrm{min}}$	ferroportin for low hepcidin	1.59E+01	fitted
$\mathrm{ZFerro}_{\mathrm{nor}}$	ferroportin for steady-state hepcidin	1.00E+00	set
$\mathrm{ZFerroport}_{\mathrm{b}}$	Sensitivity of Ferroportin regulation	5.73E-02	fitted
$\mathrm{FeDiet}_{\mathrm{nor}}$	iron from diet	3.64E-01	set
k_{HEPFS}	F_{S} influence on hepcidin production	7.80E-03	fitted
k_{HEPFP}	F_{P} influence on hepcidin production	2.24E-02	fitted
k_{HEPHB}	HB influence on hepcidin production	6.74E-04	fitted
$\mathrm{HEP}_{\mathrm{max}}$	maximum hepcidin production	2.24E+00	fitted
$\mathrm{HEP}_{\mathrm{min}}$	minimum hepcidin production	4.22E-03	fitted
$\mathrm{HEP}_{\mathrm{nor}}$	hepcidin production in steady-state	1.00E+00	set
$\mathrm{HEP}_{\mathrm{b}}$	sensitivity of hepcidin production	1.10E+00	fitted
d_{HEP}	degradation of hepcidin	1.00E+00	fitted
α	non-linearity of iron uptake	1.00E+00	set
$\mathrm{ZFerroport}_{0}$	steady-state value of ferroportin efficacy	1.00E+00	set
$\mathrm{Fe}_{\mathrm{ERY}}^{\mathrm{out\_nor}}$	steady-state of iron influx into recycling	2.23E-01	set
k_{S}	iron transfer to storage	1.30E+00	fitted
k_{HB}	iron recycling rate from HB	6.36E-01	set
$\mathrm{Fe}_{\mathrm{intest\,max}}$	maximal intestinal absorption of iron	1.82E+03	set
$\mathrm{Delay}_{\mathrm{intest}}$	delay of intestinal iron absorption	4.17E-02	set
d_{entero}	loss rate of enterocytes	1.50E+00	set
$\mathrm{k}_{\mathrm{intest}}$	iron absorption rate of enterocytes	1.30E+03	set

Table S8: **Initial values and normal values (set).**

F_{S0}	1.00E+00
F_{HB0}	1.00E+00
$TRFu_0$	1.00E+00
$TRFl_0$	1.00E+00
TRF_0	3.00E+00
$FERR_{P0}$	1.00E+00
$TSAT_0$	3.33E-01
HEP_0	1.00E+00
F_{HBnor}	6.00E+02
TRF_{nor}	3.00E+00
TRF_{lnor}	1.00E+00
TRF_{unor}	2.00E+00
F_{Snor}	1.00E+00
$TSAT_{nor}$	3.33E-01
F_{Pnor}	1.00E+03
HEP_{nor}	1.00E+00
HEP_{normal}	1.73E+01
$FERR_{Pnor}$	6.39E+01
$NTBI_{nor}$	1.00E+01

S1.4 Pharmacokinetic Model of Erythropoietin

The pharmacokinetic model of EPO is based on [2, 8, 9] consisting of three compartments: EPO in central serum ($C^{\text{cent}}_{\text{EPO}}$), EPO protein binding (peripheral compartment ($C^{\text{peri}}_{\text{EPO}}$) and EPO bound to receptors ($C^{\text{rb}}_{\text{EPO}}$). The corresponding model equations are summarised in the following.

S1.4.1 Central compartment

$$\begin{aligned} \frac{dC^{\text{cent}}_{\text{EPO}}}{dt} &= P^{\text{endo}}_{\text{EPO}}(t) - k_{\text{on}} \cdot R(t) \cdot C^{\text{cent}}_{\text{EPO}}(t) + k_{\text{off}} \cdot C^{\text{rb}}_{\text{EPO}}(t) - k_{\text{el}} \cdot C^{\text{cent}}_{\text{EPO}}(t) \\ &\quad - k_{pt} \cdot C^{\text{cent}}_{\text{EPO}}(t) + k_{tp} \cdot \frac{C^{\text{peri}}_{\text{EPO}}(t)}{\text{EPO}_{\text{Vc}}} + \frac{\text{EPO}^{\text{exogen}}(t)}{\text{EPO}_{\text{Vc}}} \qquad \text{(S1.1)} \\ C^{\text{cent}}_{\text{EPO}}(0) &= \text{EPO}_{\text{serum}} \qquad \text{(S1.2)} \end{aligned}$$

$$P^{\text{endo}}_{\text{EPO}}(t) = \left(C^{\text{cent}}_{\text{EPO}}(0) \cdot k_{\text{el}} - C^{\text{rb}}_{\text{EPO}}(0) \cdot k_{\text{off}} + k_{\text{on}} \cdot R(0) \cdot C^{\text{cent}}_{\text{EPO}}(0)\right) \cdot \text{EPO}_{\text{prod}}(t), \qquad \text{(S1.3)}$$

S1.4.2 Peripheral compartment

$$\begin{aligned} \frac{dC^{\text{peri}}_{\text{EPO}}}{dt} &= k_{pt} \cdot C^{\text{cent}}_{\text{EPO}}(t) \cdot \text{EPO}_{\text{Vc}} - k_{tp} \cdot C^{\text{peri}}_{\text{EPO}}(t) \\ \frac{dC^{\text{rb}}_{\text{EPO}}}{dt} &= k_{\text{on}} \cdot R(t) \cdot C^{\text{cent}}_{\text{EPO}}(t) - (k_{\text{off}} + k_{\text{int}}) \cdot C^{\text{rb}}_{\text{EPO}}(t). \end{aligned}$$

S1.4.3 EPO-receptors

$$\frac{dR}{dt} = k_{\text{off}} \cdot C^{\text{rb}}_{\text{EPO}}(t) - k_{\text{on}} \cdot R(t) \cdot C^{\text{cent}}_{\text{EPO}}(t) - k_{\text{deg}} \cdot R(t) + k_{\text{syn}} \cdot R^{\text{rel}}(t) \qquad \text{(S1.4)}$$

$$R^{\text{rel}}(t) = \frac{w_{\text{RET}} \cdot C_{\text{RET}} + w_{\text{MEB}} \cdot C_{\text{MEB}} + w_{\text{PEB}} \cdot C_{\text{PEB}} + w_{\text{CE}} \cdot C_{\text{CE}} + w_{\text{BE}} \cdot C_{\text{BE}}}{w_{\text{RET}} \cdot C^{\text{nor}}_{\text{RET}} + w_{\text{MEB}} \cdot C^{\text{nor}}_{\text{MEB}} + w_{\text{PEB}} \cdot C^{\text{nor}}_{\text{PEB}} + w_{\text{CE}} \cdot C^{\text{nor}}_{\text{CE}} + w_{\text{BE}} \cdot C^{\text{nor}}_{\text{BE}}} \qquad \text{(S1.5)}$$

$$\begin{aligned} C^{\text{rb}}_{\text{EPO}}(0) &= \frac{k_{\text{on}}}{k_{\text{off}} + k_{\text{int}}} \cdot R(0) \cdot C^{\text{cent}}_{\text{EPO}}(0) \\ P^{\text{endo}}_{\text{EPO}}(0) &= C^{\text{cent}}_{\text{EPO}}(0) \cdot k_{\text{el}} - C^{\text{rb}}_{\text{EPO}}(0) \cdot k_{\text{off}} + k_{\text{on}} \cdot R(0) \cdot C^{\text{cent}}_{\text{EPO}}(0) \\ C^{\text{peri}}_{\text{EPO}}(0) &= \frac{k_{12}}{k_{21}} \cdot C^{\text{cent}}_{\text{EPO}}(0) \end{aligned}$$

S1.4.4 Internalised EPO

($C^{\text{rel}}_{\text{EPO\_int}}$) regulates namely amplifications (A) and transition times (T).

$$\begin{aligned} C_{\text{EPO\_int}}(t) &= C^{\text{rb}}_{\text{EPO}}(t) \cdot k_{\text{int}} \\ C^{\text{rel}}_{\text{EPO\_int}}(t) &= \frac{C_{\text{EPO\_int}}(t)}{C_{\text{EPO\_int}}(0)}. \end{aligned}$$

S1.4.5 EPO Injections

$$\text{EPO}^{\text{inj}}(t) = \frac{\text{EPO}^{\text{dose}}}{\text{EPO}_{\text{t\_inf}}} \cdot \sum_{i=1}^{N} \big(\text{Hv}(t - \tilde{t_i}) - \text{Hv}(t - \tilde{t_i} - \text{EPO}_{\text{t\_inf}})\big) \quad \text{(S1.6)}$$

$$\text{Hv}(t) = \begin{cases} 0 : t \le 0 \\ 1 : t > 0 \end{cases} .$$

($\text{EPO}_{\text{t\_inf}}$) is set to five minutes. The applied dose is normalised to this injection time.

$$\frac{d}{dt} C^{\text{SC}}_{\text{EPO}}(t) = \text{EPO}^{\text{inj}}(t) - C^{\text{SC}}_{\text{EPO}}(t) \cdot \big(k_a^F + k_{FL} + k_e^F\big), \quad \text{(S1.7)}$$
$$C^{\text{SC}}_{\text{EPO}}(0) = 0.$$

$$\frac{dC^{(i)}_{\text{EPO\_F}}(t)}{dt} = C^{(i-1)}_{\text{EPO\_F\_out}}(t) - k^{\text{F}}_{\text{Delay}} \cdot C^{(i)}_{\text{EPO\_F}}(t), \quad i = 1, \ldots, 4 \quad \text{(S1.8)}$$
$$C^{(0)}_{\text{EPO\_F\_out}}(t) = C^{\text{SC}}_{\text{EPO}}(t) \cdot k_a^F \quad \text{(S1.9)}$$

$$C^{(i)}_{\text{EPO\_F\_out}}(t) = k^{\text{F}}_{\text{Delay}} \cdot C^{(i)}_{\text{EPO\_F}}(t) \quad i = 1, \ldots, 4$$

$$\frac{dC^{(i)}_{\text{EPO\_L}}(t)}{dt} = C^{(i-1)}_{\text{EPO\_L\_out}}(t) - k^{\text{L}}_{\text{Delay}} \cdot C^{(i)}_{\text{EPO\_L}}(t), \quad i = 1, \ldots, 4 \quad \text{(S1.10)}$$
$$C^{(0)}_{\text{EPO\_L\_out}}(t) = C^{\text{SC}}_{\text{EPO}}(t) \cdot k_{FL} \quad \text{(S1.11)}$$
$$C^{(i)}_{\text{EPO\_L\_out}}(t) = k^{\text{L}}_{\text{Delay}} \cdot C^{(i)}_{\text{EPO\_L}}(t) \quad i = 1, \ldots, 4 \quad \text{(S1.12)}$$

$$\frac{d}{dt} C^{\text{L}}_{\text{EPO}}(t) = C^{(4)}_{\text{EPO\_L\_out}}(t) - C^{\text{L}}_{\text{EPO}}(t) \cdot (k_a^L + k_e^L), \quad \text{(S1.13)}$$
$$C^{\text{L}}_{\text{EPO}}(0) = 0 \quad \text{(S1.14)}$$

$$\text{EPO}^{\text{exogen}}(t) = C^{\text{L}}_{\text{EPO}}(t) \cdot k_a^L + C^{(4)}_{\text{EPO\_F\_out}}(t), \text{EPO}^{\text{exogen}}(t) = \qquad \text{EPO}^{\text{inj}}(t) \quad \text{(S1.15)}$$

S1.5 Cell Kinetic Model

The equations in this chapter and detailed explanations are already published in [1–5, 10].

Amplification Splitting

In compartments BE, CE, and PEB the influx and efflux of cells were amplified with the over-all amplification ($A^{\text{in}}_X(t) \cdot A^{\text{out}}_X(t) = A_X(t)$) [1].

Self renewal probability p

$$p = F\big(C^{\text{rel}}_S(t), C^{\text{rel}}_E(t), C^{\text{rel}}_G(t), p_\delta, \theta_E, \theta_G, \theta_S\big).$$

$C_G^{\text{rel}}(t) = 1$.

$$p_\delta = p^{nor} - p^{min} = p^{max} - p^{nor}$$
$$\theta_S(t) = \begin{cases} \frac{2}{C_S^{\text{rel}}(t)^{0.6}} & \text{for } C_S^{\text{rel}}(t) \le 1 \\ 2 & \text{for } C_S^{\text{rel}}(t) \le 1 \end{cases}$$
$$p = p_\delta \tanh\Big(- \theta_S(t)\big(C_S^{\text{rel}}(t) - 1\big) - \theta_E(t)\big(C_E^{\text{rel}}(t) - 1\big)\Big) + 0.5\ .$$

Proliferative Fraction a_X

$$a_X = F\big(C_S^{\text{rel}}(t), C_E^{\text{rel}}(t), C_G^{\text{rel}}(t), a_X^{\text{min}}, a_X^{\text{nor}}, a_X^{\text{int}}, a_X^{\text{max}}, \omega_E, \omega_G, \omega_S\big),$$

$$y = -\frac{1}{2\ln 2}\left(\ln\left(\frac{a_X^{\text{int}} - a_X^{\text{max}}}{a_X^{\text{min}} - a_X^{\text{int}}}\right) - \ln\left(\frac{a_X^{\text{nor}} - a_X^{\text{max}}}{a_X^{\text{min}} - a_X^{\text{nor}}}\right)\right)x + \frac{1}{2}\ln\left(\frac{a_X^{\text{nor}} - a_X^{\text{max}}}{a_X^{\text{min}} - a_X^{\text{nor}}}\right)$$

$$a_X = \begin{cases} \frac{a_X^{\text{max}} e^{-y} + a_X^{\text{min}} e^{y}}{e^{-y} + e^{y}} & \text{for} \quad a_X^{\text{min}} < a_X^{\text{nor}} < a_X^{\text{int}} < a_X^{\text{max}} \\ a_X^{\text{nor}} & \text{for} \quad a_X^{\text{min}} = a_X^{\text{nor}} = a_X^{\text{int}} = a_X^{\text{max}} \end{cases}$$

$$x = \omega_E \ln C_E^{\text{rel}}(t) + \omega_S \begin{cases} \ln C_S^{\text{rel}}(t), & \text{for} \quad C_S^{\text{rel}} \le 1 \\ C_S^{\text{rel}}(t) - 1, & \text{for} \quad C_S^{\text{rel}} > 1 \end{cases}$$

Stem cell compartment S

$$\frac{d}{dt} C_S = (2p - 1) C_S \frac{a_S}{\tau_S} - \Psi_S \cdot C_S \tag{S1.16}$$
$$C_S^{\text{out}} = 2(1 - p) C_S \frac{a_S}{\tau_S} \tag{S1.17}$$

$$C_S(0) = C_S^{\text{nor}} = 1 \tag{S1.18}$$
$$C_S^{\text{out}}(0) = C_S^{\text{out\_nor}} = 2(1 - p^{\text{nor}}) C_S^{\text{nor}} \frac{a_S^{\text{nor}}}{\tau_S}. \tag{S1.19}$$

Compartment BE

$$\frac{d}{dt} C_{\text{BE}} = \alpha_E C_S^{\text{out}} A_{\text{BE}}^{\text{in}} - C_{\text{BE}} \frac{a_{\text{BE}}}{\tau_{\text{BE}}} - \Psi_{\text{BE}} \cdot C_{\text{BE}}$$
$$C_{\text{BE}}^{\text{out}} = C_{\text{BE}} A_{\text{BE}}^{\text{out}} \frac{a_{\text{BE}}}{T_{\text{BE}}}$$

$$C_{\text{BE}}(0) = C_{\text{BE}}^{\text{nor}} = \alpha_E C_S^{\text{out\_nor}} A_{\text{BE}}^{\text{in\_nor}} \frac{T_{\text{BE}}^{\text{nor}}}{a_{\text{BE}}^{\text{nor}}}$$
$$C_{\text{BE}}^{\text{out\_nor}} = C_{\text{BE}}^{\text{nor}} A_{\text{BE}}^{\text{out\_nor}} \frac{a_{\text{BE}}^{\text{nor}}}{T_{\text{BE}}^{\text{nor}}} = \alpha_E C_S^{\text{out\_nor}} A_{\text{BE}}^{\text{nor}}.$$

Compartment CE

$$\begin{aligned}
A_{\mathrm{CE}} &= Z(C^{\mathrm{rel}}_{EPO}) \\
\frac{d}{dt}C_{\mathrm{CE}} &= C^{\mathrm{out}}_{\mathrm{BE}} A^{\mathrm{in}}_{\mathrm{CE}} - C_{\mathrm{CE}} \frac{a_{\mathrm{CE}}}{T_{\mathrm{CE}}} - \Psi_{\mathrm{CE}} \cdot C_{\mathrm{CE}} \\
C^{\mathrm{out}}_{\mathrm{CE}} &= C_{\mathrm{CE}} A^{\mathrm{out}}_{\mathrm{CE}} \frac{a_{\mathrm{CE}}}{T_{\mathrm{CE}}}.
\end{aligned}$$

$a_{\mathrm{CE}} = 1$

$$\begin{aligned}
C_{\mathrm{CE}}(0) &= C^{\mathrm{nor}}_{\mathrm{CE}} = C^{\mathrm{out_nor}}_{\mathrm{BE}} A^{\mathrm{in_nor}}_{\mathrm{CE}} T^{\mathrm{nor}}_{\mathrm{CE}} \\
C^{\mathrm{out}}_{\mathrm{CE}}(0) &= C^{\mathrm{out_nor}}_{\mathrm{CE}} = C^{\mathrm{out_nor}}_{\mathrm{BE}} A^{\mathrm{nor}}_{\mathrm{CE}}
\end{aligned}$$

Compartment MEB

$N_{\mathrm{MEB}} = 15$

$$\begin{aligned}
T_{\mathrm{MEB}} &= Z(C^{\mathrm{rel}}_{EPO}) \\
C_{\mathrm{MEB}} &= \sum_{i=1}^{N_{\mathrm{MEB}}} C_{\mathrm{MEB}_i} \\
\frac{d}{dt}C_{\mathrm{MEB}_1} &= C^{\mathrm{out}}_{\mathrm{PEB}} - C_{\mathrm{MEB}_1} \frac{N_{\mathrm{MEB}}}{T_{\mathrm{MEB}}} \\
\frac{d}{dt}C_{\mathrm{MEB}_i} &= C^{\mathrm{out}}_{\mathrm{MEB}_{i-1}} - C_{\mathrm{MEB}_i} \frac{N_{\mathrm{MEB}}}{T_{\mathrm{MEB}}}, \quad i = 2, \ldots, N_{\mathrm{MEB}} \\
C^{\mathrm{out}}_{\mathrm{MEB}_i} &= C_{\mathrm{MEB}_i} \frac{N_{\mathrm{MEB}}}{T_{\mathrm{MEB}}}, \quad i = 1, \ldots, N_{\mathrm{MEB}} \\
C^{\mathrm{out}}_{\mathrm{MEB}} &= C^{\mathrm{out}}_{\mathrm{MEB}_{N_{\mathrm{MEB}}}},
\end{aligned}$$

$$\begin{aligned}
C_{\mathrm{MEB}}(0) &= C^{\mathrm{nor}}_{\mathrm{MEB}} = C^{\mathrm{out_nor}}_{\mathrm{PEB}} T^{\mathrm{nor}}_{\mathrm{MEB}} \\
C_{\mathrm{MEB}_i}(0) &= C^{\mathrm{nor}}_{\mathrm{MEB}_i} = C^{\mathrm{out_nor}}_{\mathrm{PEB}} \frac{T^{\mathrm{nor}}_{\mathrm{MEB}}}{N_{\mathrm{MEB}}}, \quad i = 1, \ldots, N_{\mathrm{MEB}} \\
C^{\mathrm{out}}_{\mathrm{MEB}_i}(0) &= C^{\mathrm{out_nor}}_{\mathrm{MEB}_i} = C^{\mathrm{nor}}_{\mathrm{MEB}_i} \frac{N_{\mathrm{MEB}}}{T^{\mathrm{nor}}_{\mathrm{MEB}}} = C^{\mathrm{out_nor}}_{\mathrm{PEB}}, \quad i = 1, \ldots, N_{\mathrm{MEB}} \\
C^{\mathrm{out}}_{\mathrm{MEB}}(0) &= C^{\mathrm{out_nor}}_{\mathrm{MEB}} = C^{\mathrm{out_nor}}_{\mathrm{MEB}_{N_{\mathrm{MEB}}}} = C^{\mathrm{out_nor}}_{\mathrm{PEB}}.
\end{aligned}$$

Compartment RET

$$\begin{aligned}
T_{\mathrm{RET}} &= T^{\mathrm{nor}}_{\mathrm{MEB}} + T^{\mathrm{nor}}_{\mathrm{RET}} - T_{\mathrm{MEB}} \\
\frac{d}{dt}C_{\mathrm{RET}} &= C^{\mathrm{out}}_{\mathrm{MEB}} - \frac{C_{\mathrm{RET}}}{T_{\mathrm{RET}}} \\
C^{\mathrm{out}}_{\mathrm{RET}} &= \frac{C_{\mathrm{RET}}}{T_{\mathrm{RET}}} \\
C_{\mathrm{RET}}(0) &= C^{\mathrm{nor}}_{\mathrm{RET}} = C^{\mathrm{nor}}_{\mathrm{ERY}} \frac{q_{\mathrm{RET}}}{1 - q_{\mathrm{RET}}} \\
C^{\mathrm{out}}_{\mathrm{RET}}(0) &= C^{\mathrm{out_nor}}_{\mathrm{RET}} = C^{\mathrm{out_nor}}_{\mathrm{MEB}} \\
T^{\mathrm{nor}}_{\mathrm{RET}} &= \frac{C^{\mathrm{nor}}_{\mathrm{RET}}}{C^{\mathrm{out_nor}}_{\mathrm{RET}}} = \frac{q_{\mathrm{RET}}}{1 - q_{\mathrm{RET}}} \big((1 - s^{\mathrm{nor}}_{\mathrm{ERY}}) T_{\mathrm{ERY_rnd}} + s^{\mathrm{nor}}_{\mathrm{ERY}} T_{\mathrm{ERY_age}} \big)
\end{aligned}$$

Compartment ERY

[1–5]

$$
\begin{aligned}
s_{\text{ERY}} &= \exp\left(\left(\frac{C_{\text{RET}}^{\text{out}}}{C_{\text{RET}}^{\text{out\_nor}}}\right)^2 \ln s_{\text{ERY}}^{\text{nor}}\right)\\
C_{\text{ERY}} &= C_{\text{ERY\_age}} + C_{\text{ERY\_rnd}}\\
C_{\text{ERY\_age}} &= \sum_{i=1}^{N_{\text{ERY}}} C_{\text{ERY\_age\_}i}\\
\frac{d}{dt}C_{\text{ERY\_age\_1}} &= s_{\text{ERY}}C_{\text{RET}}^{\text{out}} - C_{\text{ERY\_age\_1}}\frac{N_{\text{ERY}}}{T_{\text{ERY\_age}}}\\
\frac{d}{dt}C_{\text{ERY\_age\_}i} &= C_{\text{ERY\_age\_}(i-1)}^{\text{out}} - C_{\text{ERY\_age\_}i}^{\text{out}}, \quad i = 2, \ldots, N_{\text{ERY}}\\
C_{\text{ERY\_age\_}i}^{\text{out}} &= C_{\text{ERY\_age\_}i}\frac{N_{\text{ERY}}}{T_{\text{ERY\_age}}}\\
\frac{d}{dt}C_{\text{ERY\_rnd}} &= (1 - s_{\text{ERY}})C_{\text{RET}}^{\text{out}} - C_{\text{ERY\_rnd}}\frac{1}{T_{\text{ERY\_rnd}}},
\end{aligned}
$$

$$
\begin{aligned}
C_{\text{ERY}}(0) &= C_{\text{ERY}}^{\text{nor}} = C_{\text{ERY\_age}}^{\text{nor}} + C_{\text{ERY\_rnd}}^{\text{nor}}\\
C_{\text{ERY\_age}}(0) &= C_{\text{ERY\_age}}^{\text{nor}} = \sum_{i=1}^{N_{\text{ERY}}} C_{\text{ERY\_age\_}i}^{\text{nor}} = s_{\text{ERY}}^{\text{nor}}C_{\text{RET}}^{\text{out\_nor}}T_{\text{ERY\_age}}\\
C_{\text{ERY\_age\_1}}(0) &= C_{\text{ERY\_age\_1}}^{\text{nor}} = s_{\text{ERY}}^{\text{nor}}C_{\text{RET}}^{\text{out\_nor}}\frac{T_{\text{ERY\_age}}}{N_{\text{ERY}}}
\end{aligned}
$$

$$
\begin{aligned}
C_{\text{ERY\_age\_}i}(0) &= C_{\text{ERY\_age\_}i}^{\text{nor}} = C_{\text{ERY\_age\_}i-1}^{\text{out\_nor}}\frac{T_{\text{ERY\_age}}}{N_{\text{ERY}}}, \quad i = 2, \ldots, N_{\text{ERY}}\\
&= s_{\text{ERY}}^{\text{nor}}C_{\text{RET}}^{\text{out\_nor}}\frac{T_{\text{ERY\_age}}}{N_{\text{ERY}}}, \quad i = 1, \ldots, N_{\text{ERY}}\\
C_{\text{ERY\_age\_}i}^{\text{out}}(0) &= C_{\text{ERY\_age\_}i}^{\text{out\_nor}} = C_{\text{ERY\_age\_}i}^{\text{nor}}\frac{N_{\text{ERY}}}{T_{\text{ERY\_age}}} = s_{\text{ERY}}^{\text{nor}}C_{\text{RET}}^{\text{out\_nor}}\\
C_{\text{ERY}_{\text{rnd}}}(0) &= C_{\text{ERY\_rnd}}^{\text{nor}} = (1 - s_{\text{ERY}}^{\text{nor}})C_{\text{RET}}^{\text{out\_nor}}T_{\text{ERY}_{\text{rnd}}}
\end{aligned}
$$

S1.5.1 Chemotherapy Model

[1, 2, 10, 11].

$$
\text{CHEMO}^{\text{drug}}(t) = \sum_{i=1}^{N^{\text{cycle}}} \left(\text{Hv}(t - \tilde{t}_i) - \text{Hv}(t - \tilde{t}_i - t_{\text{inf}}^{\text{CHEMO}})\right) \tag{S1.20}
$$

N^{cycle} is the number of chemotherapy cycles and $t_{\text{inf}}^{\text{CHEMO}}$ is the duration of chemotherapy application.

$$
\frac{d\Psi_{\text{drug}}^{(i)}(t)}{dt} = \Psi_{\text{drug\_out}}^{(i-1)}(t) - k_{\text{Delay}}^{\text{drug}} \cdot \Psi_{\text{drug}}^{(i)}(t), \quad i = 1, \ldots, 4, \tag{S1.21}
$$

$$
\begin{aligned}
\Psi_{\text{drug}}^{(0)}(t) &= \text{CHEMO}^{\text{drug}}(t),\\
\Psi_{\text{drug\_out}}^{(i)}(t) &= k_{\text{Delay}}^{\text{drug}} \cdot \Psi_{\text{drug}}^{(i)}(t).
\end{aligned}
$$

$\Psi^{(4)}_{\mathrm{drug\_out}}(t)$ is multiplied by the toxicity parameters of the compartments $k^{\mathrm{drug}}_{\mathrm{S}}$, $k^{\mathrm{drug}}_{\mathrm{BE}}$, $k^{\mathrm{drug}}_{\mathrm{CE}}$, $k^{\mathrm{drug}}_{\mathrm{PEB}}$, $k^{\mathrm{drug}}_{\mathrm{MEB}}$, and $k^{\mathrm{drug}}_{\mathrm{RET}}$ respectively.

References

[1] Scholz, M., Engel, C. & Loeffler, M. Modelling human granulopoiesis under polychemotherapy with g-csf support. *J Math Biol* **50**, 397–439 (2005).

[2] Schirm, S., Scholz, M., Loeffler, M. & Engel, C. A biomathematical model of human erythropoiesis under erythropoietin and chemotherapy administration. *PLoS ONE* **8**, doi:10.1371/journal.pone.0065630 (2013).

[3] Loeffler, M., Pantel, K., Wulff, H. & Wichmann, H. A mathematical model of erythropoiesis in mice and rats. part 1: Structure of the model. *Cell Tissue Kinet* **22**, 13–30 (1989).

[4] Wichmann, H. & Loeffler, M. *Mathematical modeling of cell proliferation: Stem cell regulation in hemopoiesis, Vol. 1, 2.* (CRC Press, 1985).

[5] Pantel, K. *Erweiterung eines kybernetischen Modelles der Erythropoese und dessen Anwendung für normale und pathologische Mäuse. Dissertation, Universität zu Köln (in german)* (1987).

[6] Wichmann, H. *Computer modeling of erythropoiesis. In: Current Concepts in Erythropoiesis, Chapter V.* (John Wiley and Sons, 1983).

[7] Franke, M. & Schmitz, S. *Modell Mensch aus Übersicht über die Modelle zur Hämatopoese 1979-1993.* (Universität zu Köln, 1993).

[8] Krzyzanski, W. & Wyska, E. Pharmacokinetics and pharmacodynamics of erythropoietin receptor in healthy volunteers. *Naunyn-Schmiedeberg's Arch Pharmacol* **377**, 637–645 (2008).

[9] Kota, J. *et al.* Lymphatic absorption of subcutaneously administered proteins: Influence of different injection sites on the absorption of darbepoetin alfa using a sheep model. *Drug Metab Dispos* **35**, 2211–2217 (2007).

[10] Scholz, M., Gross, A. & Loeffler, M. A biomathematical model of human thrombopoiesis under chemotherapy. *J Theor Biol* **264**, 287–300 (2010).

[11] Scholz, M., Schirm, S., Wetzler, M., Engel, C. & Loeffler, M. Pharmacokinetic and -dynamic modelling of g-csf derivatives in humans. *Theoretical Biology and Medical Modelling* **9:32, doi:10.1186/1742-4682-9-32** (2012).
