# Supplementary material for: A biomathematical model of human erythropoiesis and iron metabolism
Source: Sci Rep. 2020 May 25;10:8602. doi: 10.1038/s41598-020-65313-5 (PMC7248076; doi:10.1038/s41598-020-65313-5)
Supplement: Supplementary file 2 — A biomathematical model of human erythropoiesis and iron metabolism: supplemental figures. [file 41598_2020_65313_MOESM2_ESM.pdf]

A biomathematical model of human erythropoiesis and iron metabolism: supplemental figures

Sibylle Schirm[1] and Markus Scholz[1,*]

1 Institute for Medical Informatics, Statistics and Epidemiology, University of Leipzig, Leipzig, Germany

* markus.scholz@imise.uni-leipzig.de

Model behavior

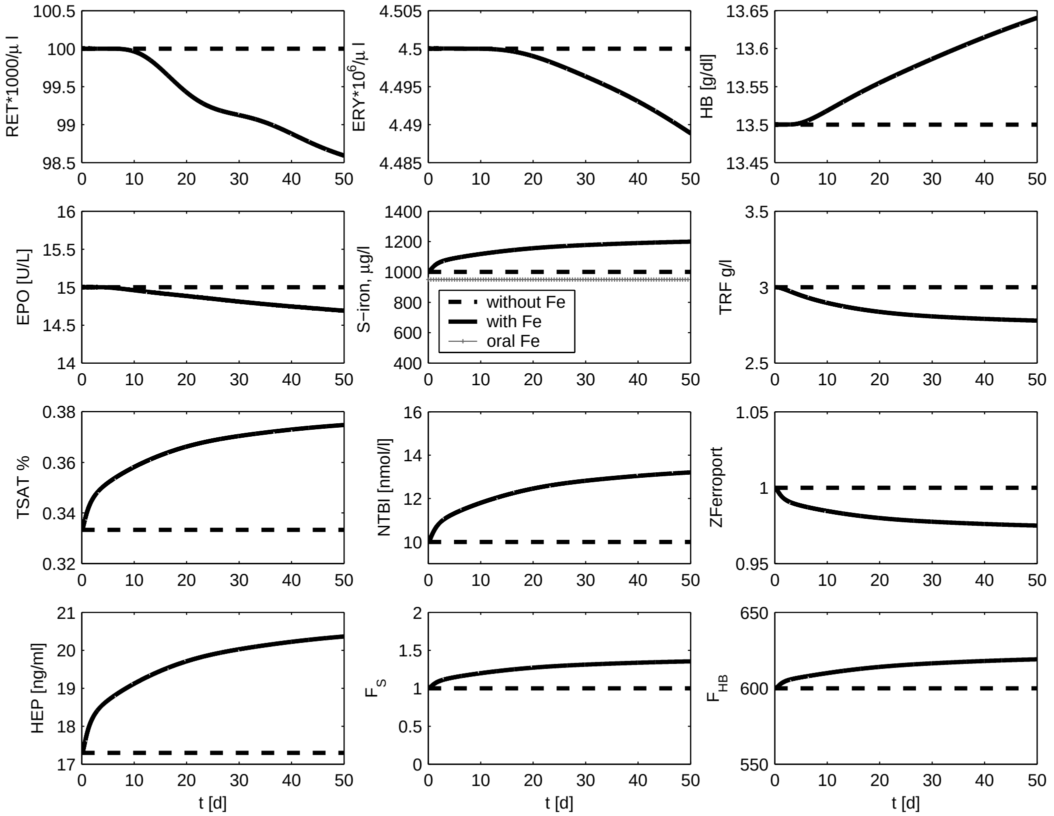

Figure S1: **Oral iron application.** Oral iron application (Fe 150 mg two times a day) in healthy individuals is simulated. The dashed lines show the steady state.

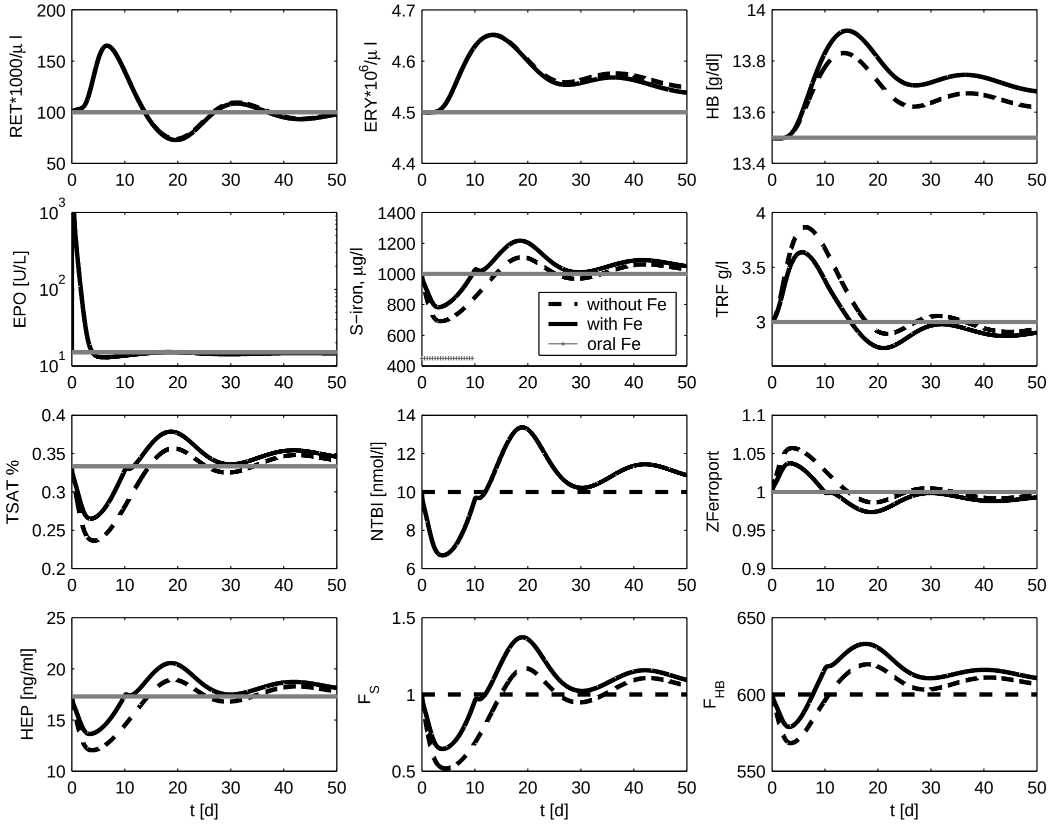

Figure S2: **Intravenous EPO injections with or without oral iron supplementation.** Simulation of intravenous injection of 200 IU/kg EPO on day 0 with 300 mg oral iron on day 0-9 (black lines) or intravenous injection of 200 IU/kg EPO on day 0 without iron supplementation (dashed lines) are simulated. Grey lines show steady state values.

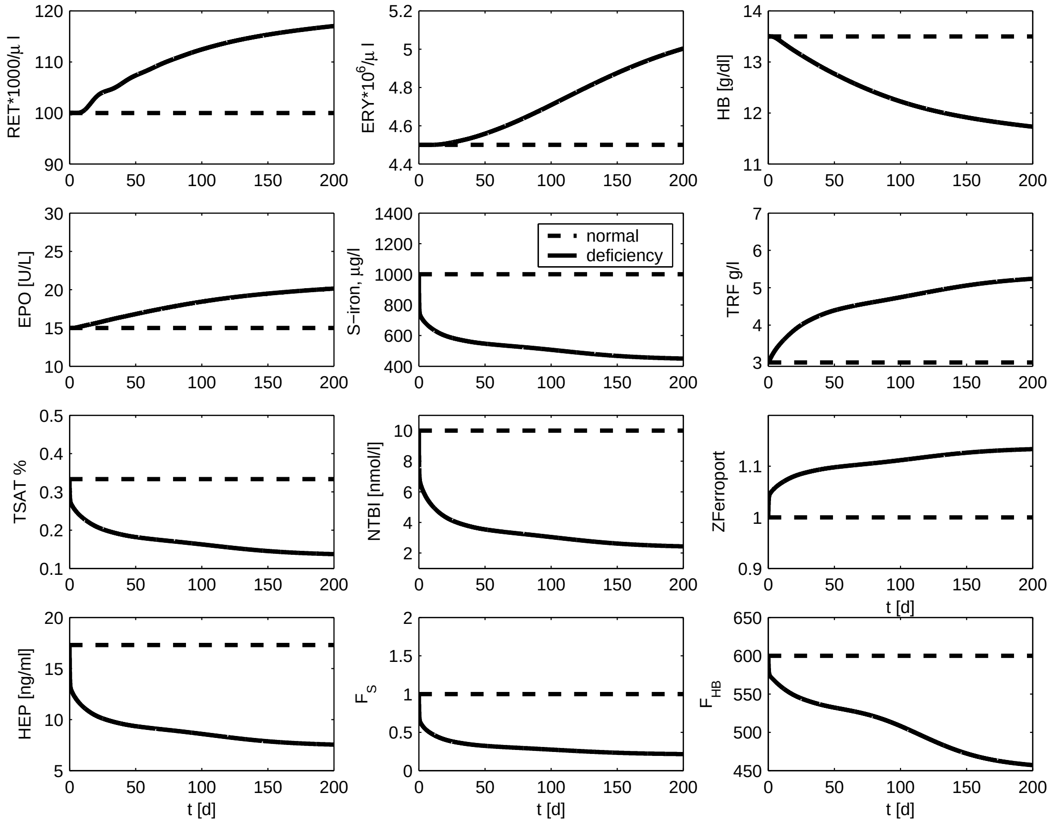

Figure S3: **Simulation of iron deficiency.** Iron malnutrition (only 50% of the required daily iron uptake is assumed) is simulated (black lines). Dashed lines show steady state values.

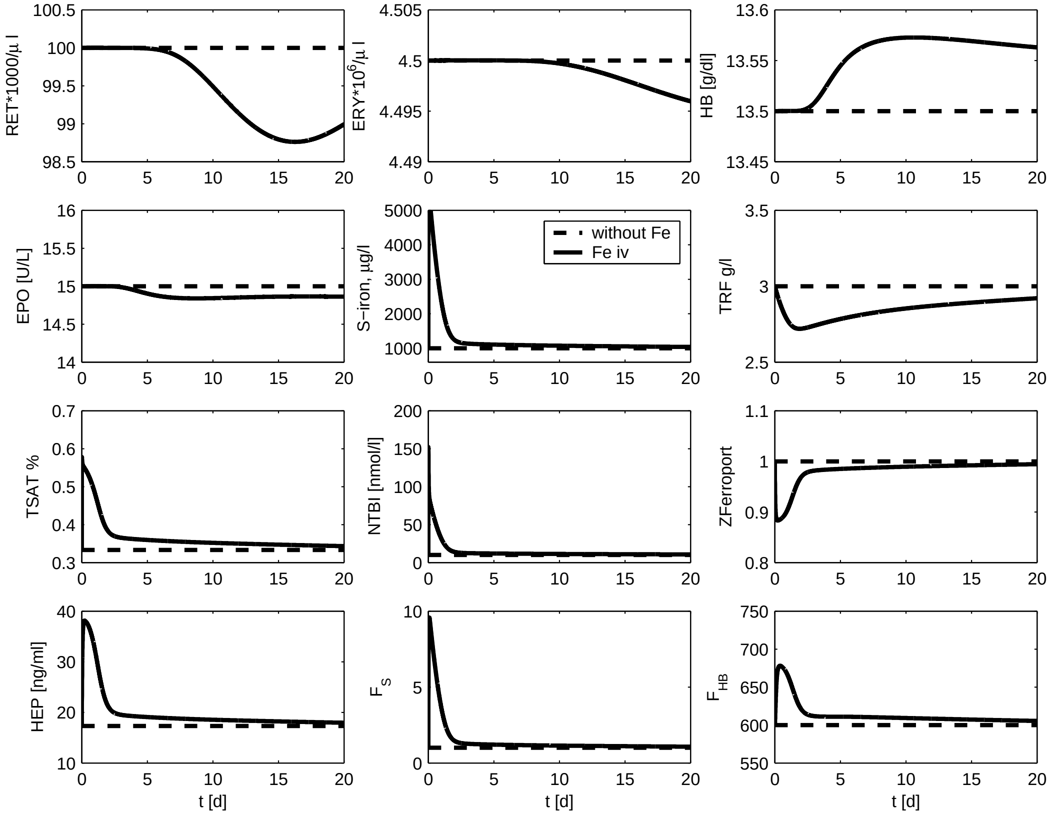

Figure S4: **Simulation of intravenous iron injection.** Intravenous iron injection of 100 mg on day 0 is simulated (black lines). The dashed lines show steady state values.

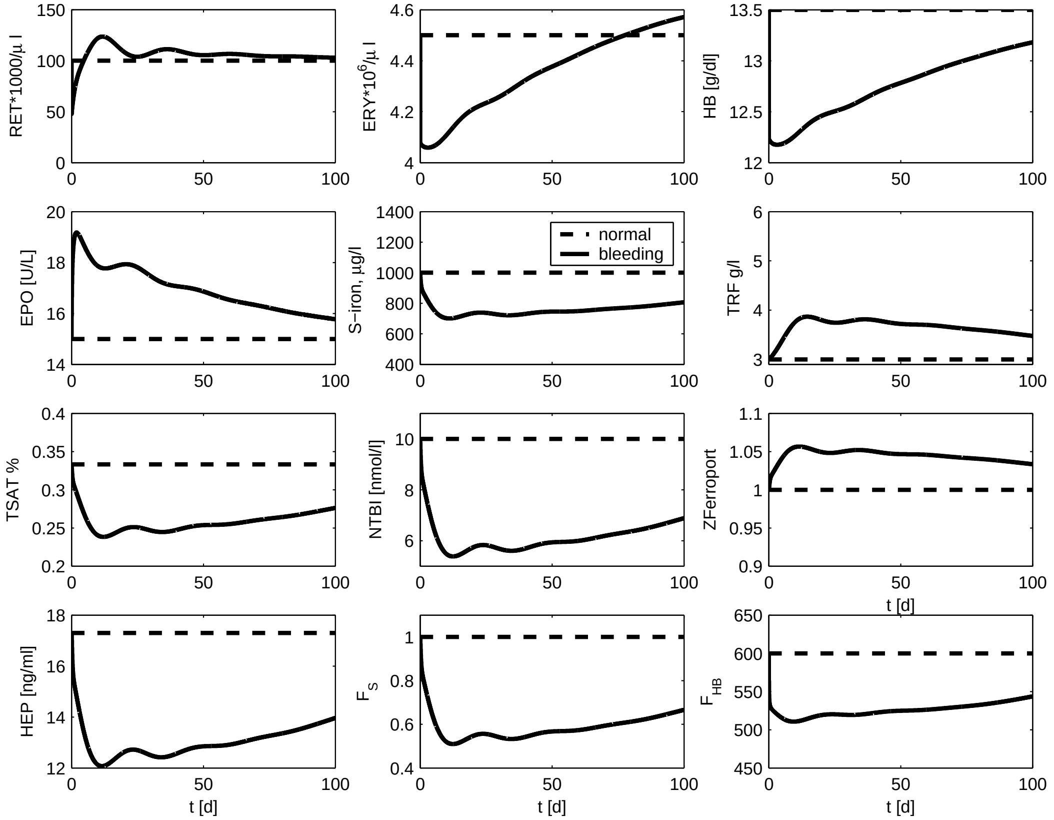

Figure S5: **Simulation of bleeding / phlebotomy.** A blood loss of about 10% is simulated (black lines). Dashed lines show steady state values.

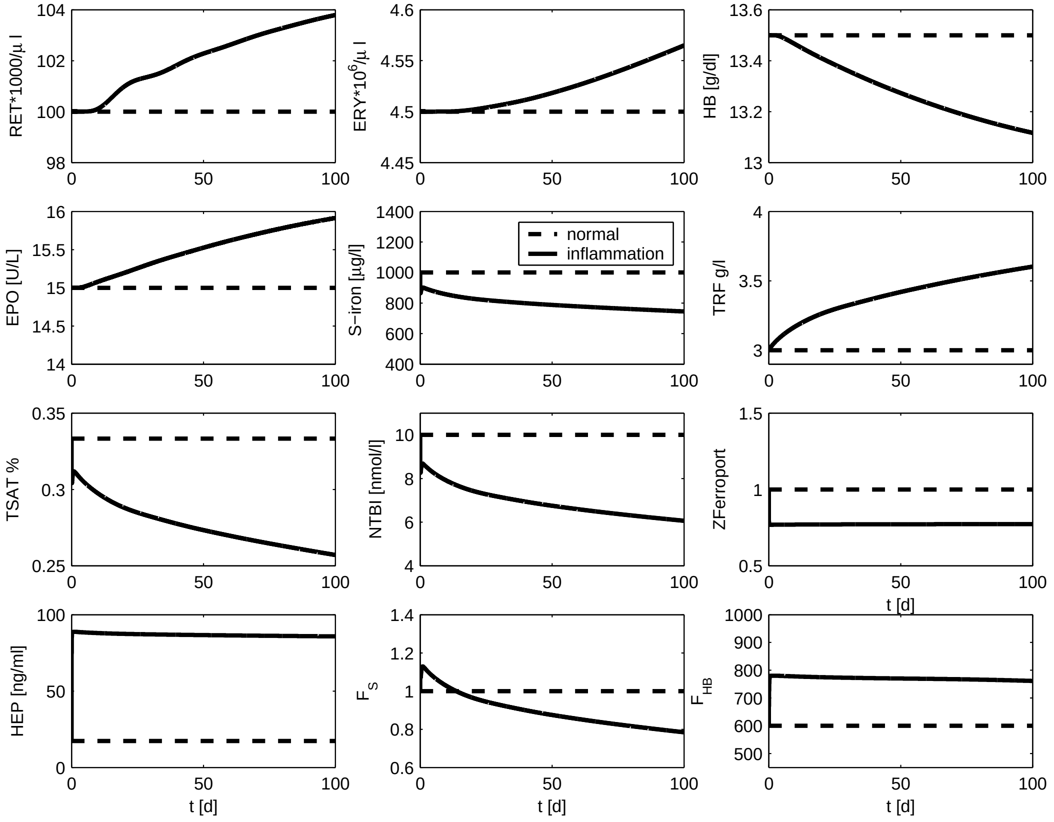

Figure S6: **Simulation of chronic inflammation.** An additional influx of hepcidin is simulated to mimic chronic inflammation (black lines). Dashed lines show the steady state levels.

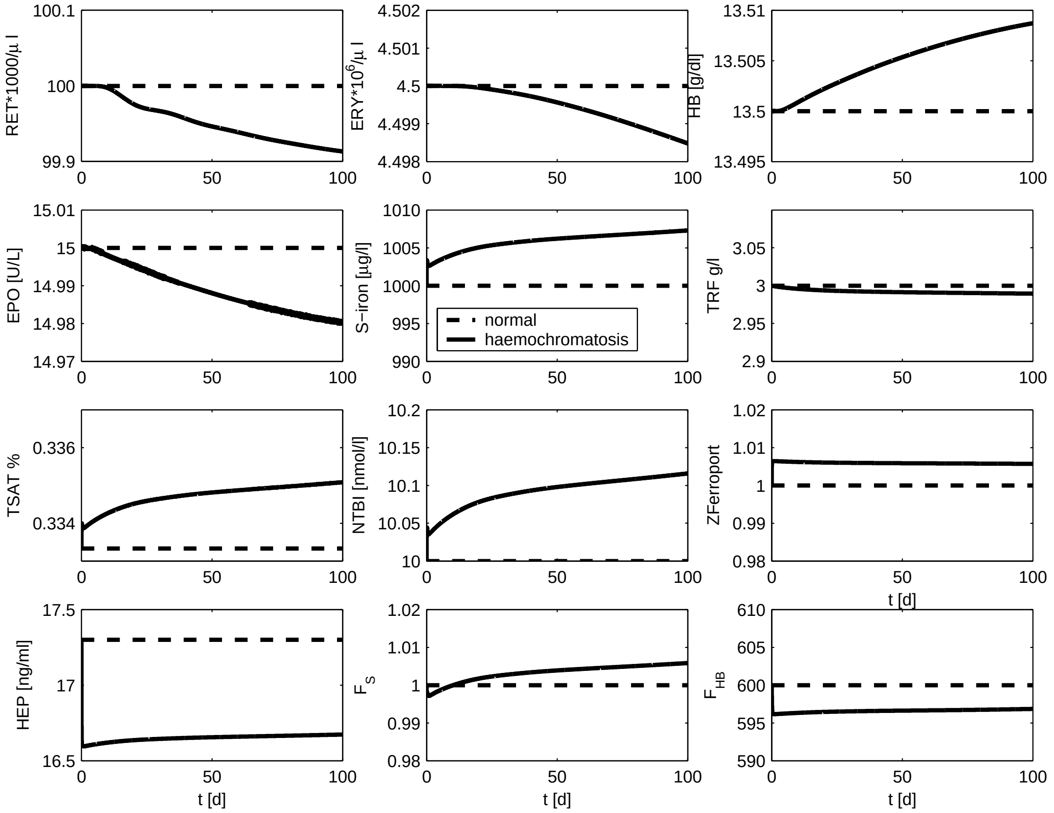

Figure S7: **Simulation of haemochromatosis.** An efflux of hepcidin is simulated to mimic haemochromatosis (black lines). Dashed lines show the steady state levels.

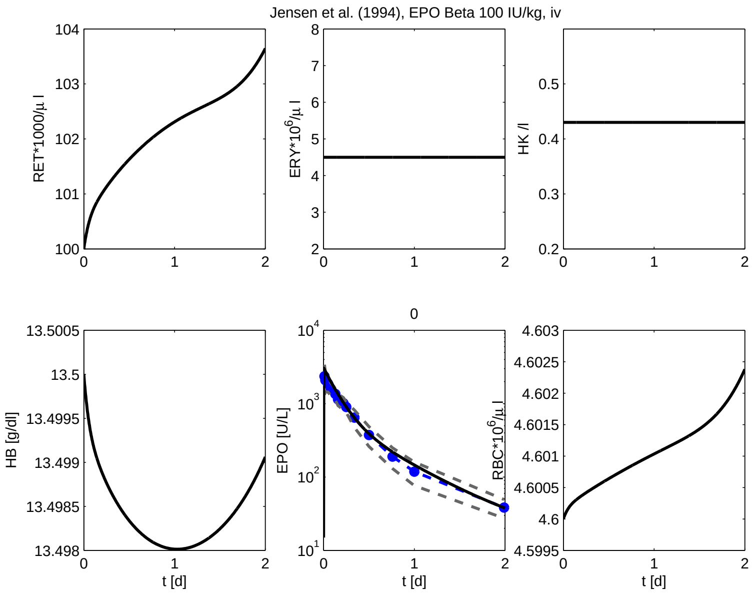

Figure S8: **Intraveneous injection of EPO.** The data from [1] were included in the fitting procedure.

References

[1] Jensen, J., Madsen, J., Jensen, L. & Pedersen, E. Reduction absorption and elimination of erythropoietin in uremia compared with healthy volunteers. *J Am Soc Nephrol* **5**, 177–185 (1994).
