# Supplementary material for: A biomathematical model of human erythropoiesis and iron metabolism
Source: Sci Rep. 2020 May 25;10:8602. doi: 10.1038/s41598-020-65313-5 (PMC7248076; doi:10.1038/s41598-020-65313-5)
Supplement: Supplementary file 3 — A biomathematical model of human erythropoiesis and iron metabolism: sensitivity analysis. [file 41598_2020_65313_MOESM3_ESM.pdf]

A biomathematical model of human erythropoiesis and iron metabolism: sensitivity analysis

Sibylle Schirm[1] and Markus Scholz[1,*]

1 Institute for Medical Informatics, Statistics and Epidemiology, University of Leipzig, Leipzig, Germany

* markus.scholz@imise.uni-leipzig.de

Sensitivity analysis

We performed a sensitivity analysis of the newly introduced parameters to determine their identifiability. For this purpose, we changed their values by 1% and calculated corresponding deteriorations of the fitness function. The parameters $\mathrm{TRFZ}_{\mathrm{max}}$ and $\mathrm{TRFZ}_{\mathrm{nor}}$ could not be changed by 1% without violating constraints. The parameters k_{PEB}, $\mathrm{TRFZ}_{\mathrm{min}}$ $\mathrm{ZFerro}_{\mathrm{min}}$, $\mathrm{ZFerro}_{\mathrm{nor}}$, k_{TRFu}, d_{TRFl}, $\mathrm{FeDiet}_{\mathrm{nor}}$, α are most sensitive, while the parameters k_{TRFHEP}, $\mathrm{ZFerro}_{\mathrm{max}}$, $\mathrm{HEP}_{\mathrm{min}}$, $\mathrm{HEP}_{\mathrm{nor}}$, and $\mathrm{Fe}_{\mathrm{intest}}^{\mathrm{max}}$ show reduced identifiability. Results are shown in figure S9.

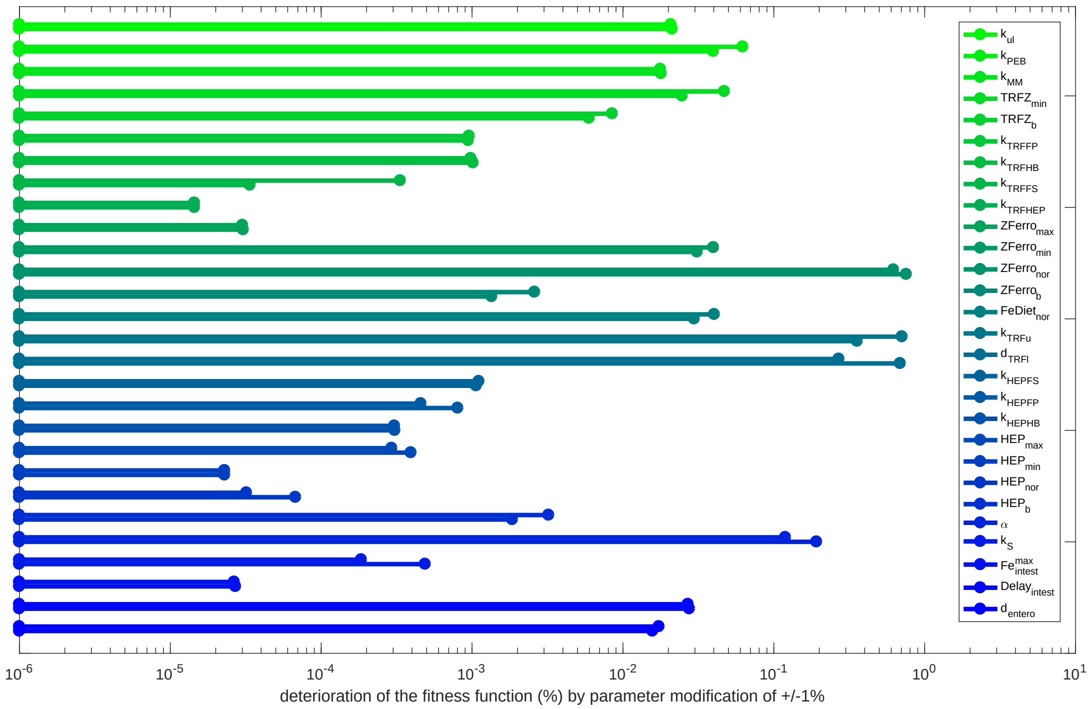

Figure S9: **Sensitivity of new model parameters.** Single parameters were modified by +/- 1 %, the other parameters were kept constant. Bars show the deterioration of the corresponding fitness function (%). Longer bars describe higher sensitivity of the parameter.
