# Supplementary material for: A biomathematical model of human erythropoiesis and iron metabolism
Source: Sci Rep. 2020 May 25;10:8602. doi: 10.1038/s41598-020-65313-5 (PMC7248076; doi:10.1038/s41598-020-65313-5)
Supplement: Supplementary file 5 — A biomathematical model of human erythropoiesis and iron metabolism: Simulation Model. [file 41598_2020_65313_MOESM5_ESM.zip › ErythroShort/Rcpp/doc/index.html]

R: Vignettes and other documentation

# Vignettes and other documentation

---

## Vignettes from package 'Rcpp'

| Rcpp::Rcpp-attributes |  | Rcpp-attributes | PDF | source |  |
| Rcpp::Rcpp-extending |  | Rcpp-extending | PDF | source |  |
| Rcpp::Rcpp-FAQ |  | Rcpp-FAQ | PDF | source |  |
| Rcpp::Rcpp-introduction |  | Rcpp-introduction | PDF | source |  |
| Rcpp::Rcpp-jss-2011 |  | Rcpp-JSS-2011 | PDF | source |  |
| Rcpp::Rcpp-modules |  | Rcpp-modules | PDF | source |  |
| Rcpp::Rcpp-package |  | Rcpp-package | PDF | source |  |
| Rcpp::Rcpp-quickref |  | Rcpp-quickref | PDF | source |  |
| Rcpp::Rcpp-sugar |  | Rcpp-sugar | PDF | source |  |
