# Supplementary material for: A biomathematical model of human erythropoiesis and iron metabolism
Source: Sci Rep. 2020 May 25;10:8602. doi: 10.1038/s41598-020-65313-5 (PMC7248076; doi:10.1038/s41598-020-65313-5)
Supplement: Supplementary file 5 — A biomathematical model of human erythropoiesis and iron metabolism: Simulation Model. [file 41598_2020_65313_MOESM5_ESM.zip › ErythroShort/Rcpp/doc/Rcpp-extending.pdf]

Rcpp Extending

Dirk Eddelbuettel[a] and Romain François[b]

[a]http://dirk.eddelbuettel.com; [b]https://romain.rbind.io/

This version was compiled on November 8, 2019

This note provides an overview of the steps programmers should follow to extend Rcpp (Eddelbuettel *et al.*, 2019a; Eddelbuettel and François, 2011) for use with their own classes. This document is based on our experience in extending Rcpp to work with the Armadillo (Sanderson, 2010) classes, available in the separate package RcppArmadillo (Eddelbuettel *et al.*, 2019b). This document assumes knowledge of Rcpp as well as some knowledge of C++ templates (Abrahams and Gurtovoy, 2004).

Rcpp | extending | R | C++

1. Introduction

Rcpp facilitates data interchange between R and C++ through the templated functions `Rcpp::as` (for conversion of objects from R to C++) and `Rcpp::wrap` (for conversion from C++ to R). In other words, we convert between the so-called S-expression pointers (in type `SEXP`) to a templated C++ type, and vice versa. The corresponding function declarations are as follows:

```
// conversion from R to C++
template <typename T> T as(SEXP x);

// conversion from C++ to R
template <typename T> SEXP wrap(const T& object);
```

These converters are often used implicitly, as in the following code chunk:

```
#include <Rcpp.h>
using namespace Rcpp;

// [[Rcpp::export]]
List fx(List input) { // we get a list from R
// pull std::vector<double> from R list
// this is achieved through an implicit
// call to Rcpp::as
std::vector<double> x = input["x"];

// return an R list; this is achieved
// through an implicit call to Rcpp::wrap
return List::create(_["front"] = x.front(),
                    _["back"]  = x.back());
}
```

Example:

```
# Run sourceCpp compilation to include file
# Rcpp::sourceCpp(file= "code.cpp")
input <- list( x = seq(1, 10, by = 0.5) )
fx(input)
#  $front
#  [1] 1
#
#  $back
#  [1] 10
```

The **Rcpp** converter functions `Rcpp::as` and `Rcpp::wrap` are extensible to user-defined types and third-party types.

2. Extending `Rcpp::wrap`

The **Rcpp::wrap** converter is extensible in essentially two ways : intrusive and non-intrusive.

2.1. Intrusive extension.

When extending **Rcpp** with your own data type, the recommended way is to implement a conversion to `SEXP`. This lets `Rcpp::wrap` know about the new data type. The template meta programming (or TMP) dispatch is able to recognize that a type is convertible to a `SEXP` and `Rcpp::wrap` will use that conversion.

The caveat is that the type must be declared before the main header file `Rcpp.h` is included.

```
#include <RcppCommon.h>

class Foo {
public:
    Foo();

    // this operator enables implicit Rcpp::wrap
    operator SEXP();
}

#include <Rcpp.h>
```

This is called *intrusive* because the conversion to `SEXP` operator has to be declared within the class.

2.2. Non-intrusive extension.

It is often desirable to offer automatic conversion to third-party types, over which the developer has no control and can therefore not include a conversion to `SEXP` operator in the class definition.

To provide automatic conversion from C++ to R, one must declare a specialization of the `Rcpp::wrap` template between the includes of `RcppCommon.h` and `Rcpp.h`.

```
#include <RcppCommon.h>

// third party library that declares class Bar
#include <foobar.h>

// declaring the specialization
namespace Rcpp {
    template <> SEXP wrap(const Bar&);
}

// this must appear after the specialization,
// otherwise the specialization will not be
// seen by Rcpp types
#include <Rcpp.h>
```

It should be noted that only the declaration is required. The implementation can appear after the `Rcpp.h` file is included, and therefore take full advantage of the **Rcpp** type system.

Another non-intrusive option is to expose an external pointer. The macro `RCPP_EXPOSED_WRAP` provides an easy way to expose a C++ class to R as an external pointer. It can be used instead of specializing `Rcpp::wrap`, and should not be used simultaneously. Note that the C++ class has to use Rcpp modules. See the Rcpp modules vignette for more details.

```
#include <Rcpp.h>
#include <foobar.h>

RCPP_EXPOSED_WRAP(Bar)
```

2.3. Templates and partial specialization. It is perfectly valid to declare a partial specialization for the `Rcpp::wrap` template. The compiler will identify the appropriate overload:

```
#include <RcppCommon.h>

// third party library that declares
// a template class Bling<T>
#include <foobar.h>

// declaring the partial specialization
namespace Rcpp {
    namespace traits {

        template <typename T>
        SEXP wrap(const Bling<T>&);

    }
}

// this must appear after the specialization, or
// specialization will not be seen by Rcpp types
#include <Rcpp.h>
```

3. Extending `Rcpp::as`

Conversion from R to C++ is also possible in both intrusive and non-intrusive ways.

3.1. Intrusive extension. As part of its template meta programming dispatch logic, `Rcpp::as` will attempt to use the constructor of the target class taking a `SEXP`.

```
#include <RcppCommon.h>

class Foo{
    public:
        Foo();

        // this ctor enables implicit Rcpp::as
        Foo(SEXP);
}

// this must appear after the specialization, or
// specialization will not be seen by Rcpp types
#include <Rcpp.h>
```

3.2. Non-intrusive extension. It is also possible to fully specialize `Rcpp::as` to enable non-intrusive implicit conversion capabilities.

```
#include <RcppCommon.h>

// third party library that declares class Bar
#include <foobar.h>

// declaring the specialization
namespace Rcpp {
    template <> Bar as(SEXP);
}

// this must appear after the specialization, or
// specialization will not be seen by Rcpp types
#include <Rcpp.h>
```

Furthermore, another non-intrusive option is to opt for sharing an R external pointer. The macro `RCPP_EXPOSED_AS` provides an easy way to extend `Rcpp::as` to expose R external pointers to C++. It can be used instead of specializing `Rcpp::as`, and should not be used simultaneously. Note that the C++ class has to use Rcpp modules. See the Rcpp modules vignette for more details.

```
#include <Rcpp.h>
#include <foobar.h>

RCPP_EXPOSED_AS(Bar)
```

With this being said, there is one additional macro that can be used to simultaneously define both `Rcpp::wrap` and `Rcpp::as` specialization for an external pointer. The macro `RCPP_EXPOSED_CLASS` can be use to transparently exchange a class between R and C++ as an external pointer. Do not simultaneously use it alongside `RCPP_EXPOSED_AS`, `RCPP_EXPOSED_WRAP`, `Rcpp::wrap`, or `Rcpp::as`.

3.3. Templates and partial specialization. The signature of `Rcpp::as` does not allow partial specialization. When exposing a templated class to `Rcpp::as`, the programmer must specialize the **Rcpp::traits::Exporter** template class. The TMP dispatch will recognize that a specialization of `Exporter` is available and delegate the conversion to this class. **Rcpp** defines the `Rcpp::traits::Exporter` template class as follows :

```
namespace Rcpp {
    namespace traits {

        template <typename T> class Exporter{
        public:
            Exporter(SEXP x) : t(x){}
            inline T get() { return t; }

        private:
            T t;
        };
    }
}
```

This is the reason why the default behavior of `Rcpp::as` is to invoke the constructor of the type `T` taking a `SEXP`.

Since partial specialization of class templates is allowed, we can expose a set of classes as follows:

```
#include <RcppCommon.h>

// third party library that declares
// a template class Bling<T>
#include <foobar.h>

// declaring the partial specialization
namespace Rcpp {
    namespace traits {
        template <typename T>
        class Exporter< Bling<T> >;
    }
}

// this must appear after the specialization, or
// specialization will not be seen by Rcpp types
#include <Rcpp.h>
```

Using this approach, the requirements for the `Exporter< Bling<T> >` class are:

- it should have a constructor taking a `SEXP`
- it should have a methods called `get` that returns an instance of the `Bling<T>` type.

4. Summary

The **Rcpp** package greatly facilitates the transfer of objects between R and C++. This note has shown how to extend **Rcpp** to either user-defined or third-party classes via the `Rcpp::as` and `Rcpp::wrap` template functions. Both intrusive and non-intrusive approaches were discussed.

References

Abrahams D, Gurtovoy A (2004). *C++ Template Metaprogramming: Concepts, Tools and Techniques from Boost and Beyond*. Addison-Wesley, Boston.

Eddelbuettel D, François R (2011). "Rcpp: Seamless R and C++ Integration." *Journal of Statistical Software*, **40**(8), 1–18. URL http://www.jstatsoft.org/v40/i08/.

Eddelbuettel D, François R, Allaire J, Ushey K, Kou Q, Russel N, Chambers J, Bates D (2019a). *Rcpp: Seamless R and C++ Integration*. R package version 1.0.3, URL http://CRAN.R-Project.org/package=Rcpp.

Eddelbuettel D, François R, Bates D, Ni B (2019b). *RcppArmadillo: Rcpp integration for Armadillo templated linear algebra library*. R package version 0.9.800.1.0, URL http://CRAN.R-Project.org/package=RcppArmadillo.

Sanderson C (2010). "Armadillo: An open source C++ Algebra Library for Fast Prototyping and Computationally Intensive Experiments." *Technical report*, NICTA. URL http://arma.sf.net.
