# Supplementary material for: A biomathematical model of human erythropoiesis and iron metabolism
Source: Sci Rep. 2020 May 25;10:8602. doi: 10.1038/s41598-020-65313-5 (PMC7248076; doi:10.1038/s41598-020-65313-5)
Supplement: Supplementary file 5 — A biomathematical model of human erythropoiesis and iron metabolism: Simulation Model. [file 41598_2020_65313_MOESM5_ESM.zip › ErythroShort/Rcpp/doc/Rcpp-FAQ.pdf]

Rcpp FAQ

Dirk Eddelbuettel[a] **and Romain François**[b]

[a]http://dirk.eddelbuettel.com; [b]https://romain.rbind.io/

This version was compiled on November 8, 2019

This document attempts to answer the most Frequently Asked Questions (FAQ) regarding the Rcpp (Eddelbuettel *et al.*, 2019a; Eddelbuettel and François, 2011; Eddelbuettel, 2013) package.

Rcpp | FAQ | R | C++

Contents

1 Getting started 1
1.1 How do I get started . . . 1
1.2 What do I need . . . 2
1.3 What compiler can I use . . . 2
1.4 What other packages are useful . . . 2
1.5 What licenses can I choose for my code . . . 2

2 Compiling and Linking 2
2.1 How do I use **Rcpp** in my package . . . 2
2.2 How do I quickly prototype my code . . . 2
2.2.1 Using inline . . . 2
2.2.2 Using Rcpp Attributes . . . 3
2.3 How do I convert my prototyped code to a package 3
2.4 How do I quickly prototype my code in a package . 3
2.5 But I want to compile my code with R CMD SHLIB 3
2.6 But R CMD SHLIB still does not work . . . 4
2.7 What about `LinkingTo` . . . 4
2.8 Does **Rcpp** work on windows . . . 4
2.9 Can I use **Rcpp** with Visual Studio . . . 4
2.10 I am having problems building Rcpp on macOS, any help . . . 4
2.10.1 Lack of a Compiler . . . 4
2.10.2 Differing macOS R Versions Leading to Binary Failures . . . 4
2.10.3 OpenMP Support . . . 4
2.10.4 Additional Information and Help . . . 4
2.11 Does **Rcpp** work on solaris/suncc . . . 4
2.12 Does **Rcpp** work with Revolution R . . . 4
2.13 Is it related to Rho (formerly CXXR) . . . 4
2.14 How do I quickly prototype my code using Attributes 5
2.15 What about the new 'no-linking' feature . . . 5
2.16 I am having problems building RcppArmadillo on macOS, any help . . . 5

3 Examples 5
3.1 Can I use templates with **Rcpp** . . . 5
3.1.1 Using inline with Templated Code . . . 5
3.1.2 Using Rcpp Attributes with Templated Code 5
3.2 Can I do matrix algebra with Rcpp . . . 5
3.2.1 Using inline with RcppArmadillo . . . 6
3.2.2 Using Rcpp Attributes with RcppArmadillo . 6
3.3 Can I use code from the Rmath header and library with **Rcpp** . . . 6
3.4 Can I use `NA` and `Inf` with **Rcpp** . . . 7
3.5 Can I easily multiply matrices . . . 7
3.6 How do I write a plugin for **inline** and/or Rcpp Attributes . . . 7
3.7 How can I pass one additional flag to the compiler 7
3.8 How can I set matrix row and column names . . . 8
3.9 Why can long long types not be cast correctly . . . 8
3.10 What LaTeX packages do I need to typeset the vignettes 8
3.11 Why is there a limit of 20 on some constructors . . 8
3.12 Can I use default function parameters with **Rcpp** . 9
3.13 Can I use C++11, C++14, C++17, . . . with **Rcpp** . 9
3.14 How do I use it within (Python's) Conda setup? . . 9
3.15 Can I speed up compilation? . . . 9

4 Support 9
4.1 Is the API documented . . . 9
4.2 Does it really work . . . 9
4.3 Where can I ask further questions . . . 10
4.4 Where can I read old questions and answers . . . 10
4.5 I like it. How can I help . . . 10
4.6 I don't like it. How can I help . . . 10
4.7 Can I have commercial support for **Rcpp** . . . 10
4.8 I want to learn quickly. Do you provide training courses . . . 10
4.9 Where is the code repository . . . 10

5 Known Issues 10
5.1 **Rcpp** changed the (const) object I passed by value . 10
5.2 Issues with implicit conversion from an **Rcpp** object to a scalar or other **Rcpp** object . . . 11
5.3 Using `operator=` with a scalar replaced the object instead of filling element-wise . . . 11
5.4 Long Vector support on Windows . . . 12
5.5 Sorting with STL on a `CharacterVector` produces problematic results . . . 12
5.6 Lexicographic order of string sorting differs due to capitalization . . . 13
5.7 Package building fails with 'symbols not found' . . . 13
5.8 Can we use exceptions and stop() across shared libraries? . . . 13

1. Getting started

1.1. How do I get started. If you have **Rcpp** installed, please execute the following command in R to access the introductory vignette (which is a variant of the Eddelbuettel and François (2011) and Eddelbuettel and Balamuta (2017, 2018) papers) for a detailed introduction, ideally followed by at least the Rcpp Attributes (Allaire *et al.*, 2019) vignette:

```r
vignette("Rcpp-jss-2011")
vignette("Rcpp-introduction")
vignette("Rcpp-attributes")
```

If you do not have **Rcpp** installed, these documents should also be available whereever you found this document, *i.e.,* on every mirror site of CRAN.

1.2. What do I need. Obviously, R must be installed. **Rcpp** provides a C++ API as an extension to the R system. As such, it is bound by the choices made by R and is also influenced by how R is configured.

In general, the standard environment for building a CRAN package from source (particularly when it contains C or C++ code) is required. This means one needs:

- a development environment with a suitable compiler (see below), header files and required libraries;
- R should be built in a way that permits linking and possibly embedding of R; this is typically ensured by the `--enable-shared-lib` option;
- standard development tools such as `make` etc.

Also see the RStudio documentation on pre-requisites for R package development.

1.3. What compiler can I use. On almost all platforms, the GNU Compiler Collection (or `gcc`, which is also the name of its C language compiler) has to be used along with the corresponding `g++` compiler for the C++ language. A minimal suitable version is a final 4.2.* release; earlier 4.2.* were lacking some C++ features (and even 4.2.1, still used on OS X as the last gcc release), has issues).

Generally speaking, the default compilers on all the common platforms are suitable.

Specific per-platform notes:

Windows users need the `Rtools` package from the site maintained by Duncan Murdoch which contains all the required tools in a single package; complete instructions specific to Windows are in the 'R Administration' manual (R Core Team, 2018a, Appendix D). As of August 2014, it still installs the `gcc/g++` 4.6.* compiler which limits the ability to use modern C++ standards so only `s-std=c++0x` is supported. R 3.1.0 and above detect this and set appropriate flags.

OS X users, as noted in the 'R Administration' manual (R Core Team, 2018a, Appendix C.4), need to install the Apple Developer Tools (*e.g.*, Xcode (OS X $\leq$ 10.8) or Xcode Command Line Tools (OS X $\geq$ 10.9) (as well as `gfortran` if R or Fortran-using packages are to be built); also see FAQ 2.10 and FAQ 2.16 below. Depending on whether on OS X release before or after Mavericks is used, different additional installation may be needed. Consult the `r-sig-mac` list (and its archives) for (current) details.

Linux user need to install the standard developement packages. Some distributions provide helper packages which pull in all the required packages; the `r-base-dev` package on Debian and Ubuntu is an example.

The `clang` and `clang++` compilers from the LLVM project can also be used. On Linux, they are inter-operable with `gcc` et al. On OS X, they are unfortunately not ABI compatible. The `clang++` compiler is interesting as it emits much more comprehensible error messages than `g++` (though `g++` 4.8 and 4.9 have caught up).

The Intel `icc` family has also been used successfully as its output files can also be combined with those from `gcc`.

1.4. What other packages are useful. Additional packages that we have found useful are:

inline which is invaluable for direct compilation, linking and loading of short code snippets—but now effectively superseded by the Rcpp Attributes (see FAQ 2.2.2 and FAQ 2.14) feature provided by **Rcpp**;

RUnit is used for unit testing; the package is recommended and will be needed to re-run some of our tests but it is not strictly required during use of **Rcpp**;

rbenchmark to run simple timing comparisons and benchmarks; it is also recommended but not required.

microbenchmark is an alternative for benchmarking.

devtools can help the process of building, compiling and testing a package but it too is entirely optional.

1.5. What licenses can I choose for my code. The **Rcpp** package is licensed under the terms of the GNU GPL 2 or later, just like R itself. A key goal of the **Rcpp** package is to make extending R more seamless. But by *linking* your code against R (as well as **Rcpp**), the combination is bound by the GPL as well. This is very clearly stated at the FSF website:

> Linking a GPL covered work statically or dynamically with other modules is making a combined work based on the GPL covered work. Thus, the terms and conditions of the GNU General Public License cover the whole combination.

So you are free to license your work under whichever terms you find suitable (provided they are GPL-compatible, see the FSF site for details). However, the combined work will remain under the terms and conditions of the GNU General Public License. This restriction comes from both R which is GPL-licensed as well as from **Rcpp** and whichever other GPL-licensed components you may be linking against.

2. Compiling and Linking

2.1. How do I use Rcpp in my package. **Rcpp** has been specifically designed to be used by other packages. Making a package that uses **Rcpp** depends on the same mechanics that are involved in making any R package that use compiled code — so reading the *Writing R Extensions* manual (R Core Team, 2018c) is a required first step.

Further steps, specific to **Rcpp**, are described in a separate vignette.

```
vignette("Rcpp-package")
```

2.2. How do I quickly prototype my code. There are two toolchains which can help with this:

- The older one is provided by the **inline** package and described in Section~2.2.1.
- Starting with **Rcpp** 0.10.0, the Rcpp Attributes feature (described in Section~2.2.2) offered an even easier alternative via the function `evalCpp`, `cppFunction` and `sourceCpp`.

The next two subsections show an example each.

2.2.1. Using inline. The **inline** package (Sklyar *et al.*, 2018) provides the functions `cfunction` and `cxxfunction`. Below is a simple function that uses `accumulate` from the (C++) Standard Template Library to sum the elements of a numeric vector.

```r
fx <- cxxfunction(signature(x = "numeric"),
    'NumericVector xx(x);
     return wrap(
              std::accumulate(xx.begin(),
                              xx.end(),
                              0.0)
            );',
    plugin = "Rcpp")
res <- fx(seq(1, 10, by=0.5))
res
#  [1] 104.5
```

One might want to use code that lives in a C++ file instead of writing the code in a character string in R. This is easily achieved by using `readLines`:

```r
fx <- cxxfunction(signature(),
                  paste(readLines("myfile.cpp"),
                        collapse="\n"),
                  plugin = "Rcpp")
```

The `verbose` argument of `cxxfunction` is very useful as it shows how **inline** runs the show.

2.2.2. Using Rcpp Attributes.

Rcpp Attributes (Allaire *et al.*, 2019), and also discussed in FAQ 2.14 below, permits an even easier route to integrating R and C++. It provides three key functions. First, `evalCpp` provide a means to evaluate simple C++ expression which is often useful for small tests, or to simply check if the toolchain is set up correctly. Second, `cppFunction` can be used to create C++ functions for R use on the fly. Third, `Rcpp::sourceCpp` can integrate entire files in order to define multiple functions.

The example above can now be rewritten as:

```r
cppFunction('double accu(NumericVector x) {
   return(
      std::accumulate(x.begin(), x.end(), 0.0)
   );
}')
res <- accu(seq(1, 10, by=0.5))
res
#  [1] 104.5
```

The `cppFunction` parses the supplied text, extracts the desired function names, creates the required scaffolding, compiles, links and loads the supplied code and makes it available under the selected identifier.

Similarly, `sourceCpp` can read in a file and compile, link and load the code therein.

2.3. How do I convert my prototyped code to a package.

Since release 0.3.5 of **inline**, one can combine FAQ 2.2.1 and FAQ 2.1. See `help("package.skeleton-methods")` once **inline** is loaded and use the skeleton-generating functionality to transform a prototyped function into the minimal structure of a package. After that you can proceed with working on the package in the spirit of FAQ 2.1.

Rcpp Attributes (Allaire *et al.*, 2019) also offers a means to convert functions written using Rcpp Attributes into a function via the `compileAttributes` function; see the vignette for details.

2.4. How do I quickly prototype my code in a package.

The simplest way may be to work directly with a package. Changes to both the R and C++ code can be compiled and tested from the command line via:

```sh
$ R CMD INSTALL mypkg && \
     Rscript --default-packages=mypkg -e \
         'someFunctionToTickle(3.14)'
```

This first installs the packages, and then uses the command-line tool `Rscript` (which ships with R) to load the package, and execute the R expression following the `-e` switch. Such an expression can contain multiple statements separated by semicolons. `Rscript` is available on all three core operating systems.

On Linux, one can also use `r` from the `littler` package by Horner and Eddelbuettel which is an alternative front end to R designed for both `#!` (hashbang) scripting and command-line use. It has slightly faster start-up times than `Rscript`; and both give a guaranteed clean slate as a new session is created.

The example then becomes

```sh
$ R CMD INSTALL mypkg && \
     r -l mypkg -e 'someFunctionToTickle(3.14)'
```

The `-l` option calls 'suppressMessages(library(mypkg))' before executing the R expression. Several packages can be listed, separated by a comma.

More choice are provide by the **devtools** package, and by using RStudio. See the respective documentation for details.

2.5. But I want to compile my code with R CMD SHLIB.

The recommended way is to create a package and follow FAQ 2.1. The alternate recommendation is to use **inline** and follow FAQ 2.2.1 because it takes care of all the details.

However, some people have shown that they prefer not to follow recommended guidelines and compile their code using the traditional `R CMD SHLIB`. To do so, we need to help `SHLIB` and let it know about the header files that **Rcpp** provides and the C++ library the code must link against.

On the Linux command-line, you can do the following:

```sh
$ # if Rcpp older than 0.11.0
$ export PKG_LIBS=`Rscript -e "Rcpp:::LdFlags()"`
$ export PKG_CXXFLAGS=\
             `Rscript -e "Rcpp:::CxxFlags()"`
$ R CMD SHLIB myfile.cpp
```

which first defines and exports two relevant environment variables which `R CMD SHLIB` then relies on. On other operating systems, appropriate settings may have to be used to define the environment variables.

This approach corresponds to the very earliest ways of building programs and can still be found in some deprecated documents (as *e.g.* some of Dirk's older 'Intro to HPC with R' tutorial slides). It is still not recommended as there are tools and automation mechanisms that can do the work for you.

Rcpp versions 0.11.0 or later can do with the definition of `PKG_LIBS` as a user-facing library is no longer needed (and hence no longer shipped with the package). One still needs to set `PKG_CXXFLAGS` to tell R where the **Rcpp** headers files are located.

Once `R CMD SHLIB` has created the dyanmically-loadable file (with extension `.so` on Linux, `.dylib` on OS X or `.dll` on Windows), it can be loaded in an R session via `dyn.load`, and the

function can be executed via `.Call`. Needless to say, we *strongly* recommend using a package, or at least Rcpp Attributes as either approach takes care of a lot of these tedious and error-prone manual steps.

2.6. But R CMD SHLIB still does not work. We have had reports in the past where build failures occurred when users had non-standard code in their `~/.Rprofile` or `Rprofile.site` (or equivalent) files.

If such code emits text on `stdout`, the frequent and implicit invocation of `Rscript -e "..."` (as in FAQ 2.5 above) to retrieve settings directly from **Rcpp** will fail.

You may need to uncomment such non-standard code, or protect it by wrapping it inside `if (interactive())`, or possibly try to use `Rscript --vanilla` instead of plain `Rscript`.

2.7. What about `LinkingTo`. R has only limited support for cross-package linkage.

We now employ the `LinkingTo` field of the `DESCRIPTION` file of packages using **Rcpp**. But this only helps in having R compute the location of the header files for us.

The actual library location and argument still needs to be provided by the user. How to do so has been shown above, and we recommned you use either FAQ 2.1 or FAQ 2.2.1 both which use the **Rcpp** function `Rcpp::LdFlags()`.

If and when `LinkingTo` changes and lives up to its name, we will be sure to adapt **Rcpp** as well.

An important change arrive with **Rcpp** release 0.11.0 and concern the automatic registration of functions; see Section~2.15 below.

2.8. Does Rcpp work on windows. Yes of course. See the Windows binaries provided by CRAN.

2.9. Can I use Rcpp with Visual Studio. Not a chance.

And that is not because we are meanies but because R and Visual Studio simply do not get along. As **Rcpp** is all about extending R with C++ interfaces, we are bound by the available toolchain. And R simply does not compile with Visual Studio. Go complain to its vendor if you are still upset.

2.10. I am having problems building Rcpp on macOS, any help. There are three known issues regarding Rcpp build problems on macOS. If you are building packages with RcppArmadillo, there is yet another issue that is addressed separately in FAQ 2.16 below.

2.10.1. Lack of a Compiler. By default, macOS does not ship with an active compiler. Depending on the R version being used, there are different development environment setup procedures. For the current R version, we recommend observing the official procedure used in Section 6.3.2 macOS and Section C.3 macOS of the R Installation and Administration manual.

2.10.2. Differing macOS R Versions Leading to Binary Failures. There are currently *three* distinct versions of R for macOS. The first version is a legacy version meant for macOS 10.6 (Snow Leopard) - 10.8 (Mountain Lion). The second version is for more recent system macOS 10.9 (Mavericks) and 10.10 (Yosemite). Finally, the third and most up-to-date version supports macOS 10.11 (El Capitan), 10.12 (Sierra), and 10.13 (High Sierra). The distinction comes as a result of a change in the compilers shipped with the operating system as highlighted previously. As a result, avoid sending **package binaries** to collaborators if they are working on older operating systems as the R binaries for these versions will not be able to mix. In such cases, it is better to provide collaborators with the **package source** and allow them to build the package locally.

2.10.3. OpenMP Support. By default, the macOS operating environment lacks the ability to parallelize sections of code using the [OpenMP](http://openmp.org/wp/) standard. Within R 3.4.*, the default developer environment was *changed* to allow for OpenMP to be used on macOS by using a non-default toolchain provided by R Core Team maintainers for macOS. Having said this, it is still important to protect any reference to OpenMP as some users may not yet have the ability to use OpenMP.

To setup the appropriate protection for using OpenMP, the process is two-fold. First, protect the inclusion of headers with:

```
#ifdef _OPENMP
  #include <omp.h>
#endif
```

Second, when parallelizing portions of code use:

```
#ifdef _OPENMP
    // multithreaded OpenMP version of code
#else
    // single-threaded version of code
#endif
```

Under this approach, the code will be *safely* parallelized when support exists for OpenMP on Windows, macOS, and Linux.

2.10.4. Additional Information and Help. Below are additional resources that provide information regarding compiling Rcpp code on macOS.

1. A helpful post was provided by Brian Ripley regarding the use of compiling R code with macOS in April 2014 on the `r-sig-mac` list, which is generally recommended for OS X-specific questions and further consultation.
2. Another helpful write-up for installation / compilation on OS X Mavericks is provided by the BioConductor project.
3. Lastly, another resource that exists for installation / compilation help is provided at http://thecoatlessprofessor.com/programming/r-compiler-tools-for-rcpp-on-os-x/.

Note: If you are running into trouble compiling code with **RcppArmadillo**, please also see FAQ 2.16 listed below.

2.11. Does Rcpp work on solaris/suncc. Yes, it generally does. But as we do not have access to such systems, some issues persist on the CRAN test systems.

2.12. Does Rcpp work with Revolution R. We have not tested it yet. **Rcpp** might need a few tweaks to work with the compilers used by Revolution R (if those differ from the defaults).

2.13. Is it related to Rho (formerly CXXR). Rho, previously known as CXXR, is an ambitious project that aims to totally refactor the R interpreter in C++. There are a few similaritites with **Rcpp** but the projects are unrelated.

Rho / CXXR and **Rcpp** both want R to make more use of C++ but they do it in very different ways.

2.14. How do I quickly prototype my code using Attributes. **Rcpp** version 0.10.0 and later offer a new feature 'Rcpp Attributes' which is described in detail in its own vignette (Allaire *et al.*, 2019). In short, it offers functions `evalCpp`, `cppFunction` and `sourceCpp` which extend the functionality of the `cxxfunction` function.

2.15. What about the new 'no-linking' feature. Starting with **Rcpp** 0.11.0, functionality provided by **Rcpp** and used by packages built with **Rcpp** accessed via the registration facility offered by R (and which is used by **lme4** and **Matrix**, as well as by **xts** and **zoo**). This requires no effort from the user / programmer, and even frees us from explicit linking instruction. In most cases, the files `src/Makevars` and `src/Makevars.win` can now be removed. Exceptions are the use of **RcppArmadillo** (which needs an entry `PKG_LIBS=$(LAPACK_LIBS) $(BLAS_LIBS) $(FLIBS)`) and packages linking to external libraries they use.

But for most packages using **Rcpp**, only two things are required:

- an entry in `DESCRIPTION` such as `Imports: Rcpp` (which may be versioned as in `Imports: Rcpp (>= 0.11.0)`), and
- an entry in `NAMESPACE` to ensure **Rcpp** is correctly instantiated, for example `importFrom(Rcpp, evalCpp)`.

The name of the symbol does not really matter; once one symbol is imported all symbols should be available.

2.16. I am having problems building RcppArmadillo on macOS, any help. Odds are your build failures are due to the absence of `gfortran` and its associated libraries. The errors that you may receive are related to either `-lgfortran` or `-lquadmath`.

To rectify the root of these errors, there are two options available. The first option is to download and use a fixed set of `gfortran` binaries that are used to compile R for macOS (e.g. given by the maintainers of the macOS build). The second option is to either use pre-existing `gfortran` binaries on your machine or download the latest. These options are described in-depth in Section C.3 macOS of the R Installation and Administration manual. Please consult this manual for up-to-date information regarding `gfortran` binaries on macOS. We have also documented *other* common macOS compile issues in Section FAQ 2.10.

3. Examples

The following questions were asked on the Rcpp-devel mailing list, which is our preferred place to ask questions as it guarantees exposure to a number of advanced Rcpp users. The StackOverflow tag for rcpp is an alternative; that site is also easily searchable.

Several dozen fully documented examples are provided at the Rcpp Gallery – which is also open for new contributions.

3.1. Can I use templates with Rcpp.

> I'm curious whether one can provide a class definition inline in an R script and then initialize an instance of the class and call a method on the class, all inline in R.

This question was initially about using templates with **inline**, and we show that (older) answer first. It is also easy with Rcpp Attributes which is what we show below.

3.1.1. Using inline with Templated Code. Most certainly, consider this simple example of a templated class which squares its argument:

```
inc <- 'template <typename T>
        class square :
          public std::unary_function<T,T> {
            public:
              T operator()( T t) const {
                return t*t;
              }
        };
       '

src <- '
       double x = Rcpp::as<double>(xs);
       int i = Rcpp::as<int>(is);
       square<double> sqdbl;
       square<int> sqint;
       return Rcpp::DataFrame::create(
                    Rcpp::Named("x", sqdbl(x)),
                    Rcpp::Named("i", sqint(i)));
       '
fun <- cxxfunction(signature(xs="numeric",
                             is="integer"),
                   body=src, include=inc,
                   plugin="Rcpp")

fun(2.2, 3L)
#       x i
#  1 4.84 9
```

3.1.2. Using Rcpp Attributes with Templated Code. We can also use 'Rcpp Attributes' (Allaire *et al.*, 2019)—as described in FAQ 2.2.2 and FAQ 2.14 above. Simply place the following code into a file and use `sourceCpp` on it. It will even run the R part at the end.

```
#include <Rcpp.h>

template <typename T> class square :
    public std::unary_function<T,T> {
      public:
      T operator()( T t) const {
        return t*t ;
      }
};

// [[Rcpp::export]]
Rcpp::DataFrame fun(double x, int i) {
       square<double> sqdbl;
       square<int> sqint;
       return Rcpp::DataFrame::create(
           Rcpp::Named("x", sqdbl(x)),
           Rcpp::Named("i", sqint(i)));
}

/*** R
fun(2.2, 3L)
*/
```

3.2. Can I do matrix algebra with Rcpp.

> **Rcpp** allows element-wise operations on vector and matrices through operator overloading and STL interface, but what if I want to multiply a matrix by a vector, etc ...

Currently, **Rcpp** does not provide binary operators to allow operations involving entire objects. Adding operators to **Rcpp** would be a major project (if done right) involving advanced techniques such as expression templates. We currently do not plan to go in this direction, but we would welcome external help. Please send us a design document.

However, we have developed the **RcppArmadillo** package (Eddelbuettel *et al.*, 2019b; Eddelbuettel and Sanderson, 2014) that provides a bridge between **Rcpp** and **Armadillo** (Sanderson, 2010). **Armadillo** supports binary operators on its types in a way that takes full advantage of expression templates to remove temporaries and allow chaining of operations. That is a mouthful of words meaning that it makes the code go faster by using fiendishly clever ways available via the so-called template meta programming, an advanced C++ technique. Also, the **RcppEigen** package (Bates and Eddelbuettel, 2013) provides an alternative using the Eigen template library.

3.2.1. Using inline with RcppArmadillo. The following example is adapted from the examples available at the project page of Armadillo. It calculates $x' \times Y^{-1} \times z$

```
lines = '// copy the data to armadillo structures
arma::colvec x = Rcpp::as<arma::colvec> (x_);
arma::mat Y = Rcpp::as<arma::mat>(Y_) ;
arma::colvec z = Rcpp::as<arma::colvec>(z_) ;

// calculate the result
double result = arma::as_scalar(
                 arma::trans(x) * arma::inv(Y) * z
                );

// return it to R
return Rcpp::wrap( result );'

writeLines(a, file = "myfile.cpp")
```

If stored in a file `myfile.cpp`, we can use it via **inline**:

```
fx <- cxxfunction(signature(x_="numeric",
                            Y_="matrix",
                            z_="numeric" ),
                  paste(readLines("myfile.cpp"),
                        collapse="\n"),
                  plugin="RcppArmadillo" )
fx(1:4, diag(4), 1:4)
```

The focus is on the code `arma::trans(x) * arma::inv(Y) * z`, which performs the same operation as the R code `t(x) %*% solve(Y) %*% z`, although Armadillo turns it into only one operation, which makes it quite fast. Armadillo benchmarks against other C++ matrix algebra libraries are provided on the Armadillo website.

It should be noted that code below depends on the version `0.3.5` of **inline** and the version `0.2.2` of **RcppArmadillo**.

3.2.2. Using Rcpp Attributes with RcppArmadillo. We can also write the same example for use with Rcpp Attributes:

```
#include <RcppArmadillo.h>

// [[Rcpp::depends(RcppArmadillo)]]

// [[Rcpp::export]]
double fx(arma::colvec x, arma::mat Y,
          arma::colvec z) {
    // calculate the result
    double result = arma::as_scalar(
        arma::trans(x) * arma::inv(Y) * z
    );
    return result;
}

/*** R
fx(1:4, diag(4), 1:4)
*/
```

Here, the additional `Rcpp::depends(RcppArmadillo)` ensures that code can be compiled against the **RcppArmadillo** header, and that the correct libraries are linked to the function built from the supplied code example.

Note how we do not have to concern ourselves with conversion; R object automatically become (Rcpp)Armadillo objects and we can focus on the single computing a (scalar) result.

3.3. Can I use code from the Rmath header and library with Rcpp.

> Can I call functions defined in the Rmath header file and the standalone math library for R–as for example the random number generators?

Yes, of course. This math library exports a subset of R, but **Rcpp** has access to much more. Here is another simple example. Note how we have to use and instance of the `RNGScope` class to set and re-set the random-number generator. This also illustrates Rcpp sugar as we are using a vectorised call to `rnorm`. Moreover, because the RNG is reset, the two calls result in the same random draws. If we wanted to control the draws, we could explicitly set the seed after the `RNGScope` object has been instantiated.

```
fx <- cxxfunction(signature(),
                  'RNGScope();
                   return rnorm(5, 0, 100);',
                  plugin="Rcpp")
set.seed(42)
fx()
#  [1] 137.096 -56.470  36.313  63.286  40.427
fx()
#  [1] 137.096 -56.470  36.313  63.286  40.427
```

Newer versions of Rcpp also provide the actual Rmath function in the `R` namespace, *i.e.* as `R::rnorm(m,s)` to obtain a scalar random variable distributed as $N(m,s)$.

Using Rcpp Attributes, this can be as simple as

```
cppFunction('Rcpp::NumericVector ff(int n) {
              return rnorm(n, 0, 100);  }')
set.seed(42)
ff(5)
#  [1] 137.096 -56.470  36.313  63.286  40.427
ff(5)
#  [1] -10.6125 151.1522  -9.4659 201.8424  -6.2714
set.seed(42)
rnorm(5, 0, 100)
#  [1] 137.096 -56.470  36.313  63.286  40.427
rnorm(5, 0, 100)
```

```
#  [1] -10.6125 151.1522  -9.4659 201.8424  -6.2714
```

This illustrates the Rcpp Attributes adds the required `RNGScope` object for us. It also shows how setting the seed from R affects draws done via C++ as well as R, and that identical random number draws are obtained.

3.4. Can I use `NA` and `Inf` with Rcpp.

> R knows about `NA` and `Inf`. How do I use them from C++?

Yes, see the following example:

```
src <- 'Rcpp::NumericVector v(4);
        v[0] = R_NegInf; // -Inf
        v[1] = NA_REAL;  // NA
        v[2] = R_PosInf; // Inf
        v[3] = 42;       // c.f. Hitchhiker Guide
        return Rcpp::wrap(v);'
fun <- cxxfunction(signature(), src, plugin="Rcpp")
fun()
#  [1] -Inf   NA  Inf   42
```

Similarly, for Rcpp Attributes:

```
#include <Rcpp.h>

// [[Rcpp::export]]
Rcpp::NumericVector fun(void) {
    Rcpp::NumericVector v(4);
    v[0] = R_NegInf; // -Inf
    v[1] = NA_REAL;  // NA
    v[2] = R_PosInf; // Inf
    v[3] = 42;       // c.f. Hitchhiker Guide
    return v;
}
```

3.5. Can I easily multiply matrices.

> Can I multiply matrices easily?

Yes, via the **RcppArmadillo** package which builds upon **Rcpp** and the wonderful Armadillo library described above in FAQ 3.2:

```
txt <- 'arma::mat Am = Rcpp::as< arma::mat >(A);
        arma::mat Bm = Rcpp::as< arma::mat >(B);
        return Rcpp::wrap( Am * Bm );'
mmult <- cxxfunction(signature(A="numeric",
                               B="numeric"),
                     body=txt,
                     plugin="RcppArmadillo")
A <- matrix(1:9, 3, 3)
B <- matrix(9:1, 3, 3)
C <- mmult(A, B)
C
```

Armadillo supports a full range of common linear algebra operations.

The **RcppEigen** package provides an alternative using the Eigen template library.

Rcpp Attributes, once again, makes this even easier:

```
#include <RcppArmadillo.h>

// [[Rcpp::depends(RcppArmadillo)]]

// [[Rcpp::export]]
arma::mat mult(arma::mat A, arma::mat B) {
    return A*B;
}

/*** R
A <- matrix(1:9, 3, 3)
B <- matrix(9:1, 3, 3)
mult(A,B)
*/
```

which can be built, and run, from R via a simple `sourceCpp` call—and will also run the small R example at the end.

3.6. How do I write a plugin for inline and/or Rcpp Attributes.

> How can I create my own plugin for use by the **inline** package?

Here is an example which shows how to it using GSL libraries as an example. This is merely for demonstration, it is also not perfectly general as we do not detect locations first—but it serves as an example:

```
# simple example of seeding RNG and
# drawing one random number
gslrng <- '
int seed = Rcpp::as<int>(par) ;
gsl_rng_env_setup();
gsl_rng *r = gsl_rng_alloc (gsl_rng_default);
gsl_rng_set (r, (unsigned long) seed);
double v = gsl_rng_get (r);
gsl_rng_free(r);
return Rcpp::wrap(v);'

plug <- Rcpp::Rcpp.plugin.maker(
    include.before = "#include <gsl/gsl_rng.h>",
    libs = paste(
"-L/usr/local/lib/R/site-library/Rcpp/lib -lRcpp",
"-Wl,-rpath,/usr/local/lib/R/site-library/Rcpp/lib",
"-L/usr/lib -lgsl -lgslcblas -lm")
)
registerPlugin("gslDemo", plug )
fun <- cxxfunction(signature(par="numeric"),
                   gslrng, plugin="gslDemo")
fun(0)
```

Here the **Rcpp** function `Rcpp.plugin.maker` is used to create a plugin 'plug' which is then registered, and subsequently used by **inline**.

The same plugins can be used by Rcpp Attributes as well.

3.7. How can I pass one additional flag to the compiler.

> How can I pass another flag to the g++ compiler without writing a new plugin?

The quickest way is to modify the return value from an existing plugin. Here we use the default one from **Rcpp** itself in

order to pass the new flag -std=c++0x. As it does not set the PKG_CXXFLAGS variable, we simply assign this. For other plugins, one may need to append to the existing values instead.

```
myplugin <- getPlugin("Rcpp")
myplugin$env$PKG_CXXFLAGS <- "-std=c++11"
f <- cxxfunction(signature(),
                 settings = myplugin, body = '
    // fails without -std=c++0x
    std::vector<double> x = { 1.0, 2.0, 3.0 };
    return Rcpp::wrap(x);
')
f()
```

For Rcpp Attributes, the attributes Rcpp::plugin() can be used. Currently supported plugins are for C++11 as well as for OpenMP.

3.8. How can I set matrix row and column names.

> Ok, I can create a matrix, but how do I set its row and columns names?

Pretty much the same way as in R itself: We define a list with two character vectors, one each for row and column names, and assign this to the dimnames attribute:

```
src <- '
  Rcpp::NumericMatrix x(2,2);
  x.fill(42);           // or another value
  Rcpp::List dimnms =   // list with 2 vecs
    Rcpp::List::create( // with static names
      Rcpp::CharacterVector::create("cc", "dd"),
      Rcpp::CharacterVector::create("ee", "ff")
    );
  // and assign it
  x.attr("dimnames") = dimnms;
  return(x);
'
fun <- cxxfunction(signature(),
                   body=src, plugin="Rcpp")
fun()
```

The same logic, but used with Rcpp Attributes:

```
#include <Rcpp.h>

// [[Rcpp::export]]
Rcpp::List fun(void) {
    Rcpp::NumericMatrix x(2,2);
    x.fill(42);           // or another value
    Rcpp::List dimnms =   // list with 2 vecs
      Rcpp::List::create( // with static names
        Rcpp::CharacterVector::create("cc", "dd"),
        Rcpp::CharacterVector::create("ee", "ff"));
    // and assign it
    x.attr("dimnames") = dimnms;
    return(x);
}
```

3.9. Why can long long types not be cast correctly. That is a good and open question. We rely on the basic R types, notably integer and numeric. These can be cast to and from C++ types without problems. But there are corner cases. The following example, contributed by a user, shows that we cannot reliably cast long types (on a 64-bit machines).

```
BigInts <- cxxfunction(signature(),
  'std::vector<long> bigints;
   bigints.push_back(12345678901234567LL);
   bigints.push_back(12345678901234568LL);
   Rprintf("Difference of %ld\\n",
       12345678901234568LL - 12345678901234567LL);
   return wrap(bigints);',
  plugin="Rcpp", includes="#include <vector>")

retval <- BigInts()

# Unique 64-bit integers were cast to identical
# lower precision numerics behind my back with
# no warnings or errors whatsoever.  Error.

stopifnot(length(unique(retval)) == 2)
```

While the difference of one is evident at the C++ level, it is no longer present once cast to R. The 64-bit integer values get cast to a floating point types with a 53-bit mantissa. We do not have a good suggestion or fix for casting 64-bit integer values: 32-bit integer values fit into integer types, up to 53 bit precision fits into numeric and beyond that truly large integers may have to converted (rather crudely) to text and re-parsed. Using a different representation as for example from the GNU Multiple Precision Arithmetic Library may be an alternative.

3.10. What LaTeX packages do I need to typeset the vignettes.

> I would like to typeset the vignettes. What do I need?

The TeXLive distribution seems to get bigger and bigger. What you need to install may depend on your operating system.

Specific per-platform notes:

- **Windows** users probably want the MiKTeX. Suggestions for a more detailed walk through would be appreciated.
- **OS X** users seem to fall into camps which like or do not like brew / homebrew. One suggestion was to install MacTeX but at approximately 2.5gb (as of January 2016) this is not lightweight.
- **Linux** users probably want the full TeXLive set from their distribution. On Debian these packages are installed to build the R package itself: texlive-base, texlive-latex-base, texlive-generic-recommended, texlive-fonts-recommended, texlive-fonts-extra, texlive-extra-utils, texlive-latex-recommended, texlive-latex-extra. Using texlive-full may be a shortcut. Fedora and other distributions should have similar packages.

3.11. Why is there a limit of 20 on some constructors.

> Ok, I would like to pass N object but you only allow 20. How come?

In essence, and in order to be able to compile it with the largest number of compilers, **Rcpp** is constrained by the older C++ standards which do not support variadic function arguments. So we

actually use macros and code generator scripts to explicitly enumerate arguments, and that number has to stop at some limit. We chose 20.

A good discussion is available at this StackOverflow question concering data.frame creation with **Rcpp**. One solution offers a custom `ListBuilder` class to circumvent the limit; another suggests to simply nest lists.

3.12. Can I use default function parameters with Rcpp. Yes, you can use default parameters with *some* limitations. The limitations are mainly related to string literals and empty vectors. This is what is currently supported:

- String literals delimited by quotes (e.g. `"foo"`)
- Integer and Decimal numeric values (e.g. `10` or `4.5`)
- Pre-defined constants including:
  - Booleans: `true` and `false`
  - Null Values: `R_NilValue`, `NA_STRING`, `NA_INTEGER`, `NA_REAL`, and `NA_LOGICAL`.
- Selected vector types can be instantiated using the empty form of the `::create` static member function.
  - `CharacterVector`, `IntegerVector`, and `NumericVector`
- Matrix types instantiated using the rows, cols constructor `Rcpp::<Type>Matrix n(rows,cols)`
  - `CharacterMatrix`, `IntegerMatrix`, and `NumericMatrix`

To illustrate, please consider the following example that provides a short how-to:

```
#include <Rcpp.h>

// [[Rcpp::export]]
void sample_defaults(
        NumericVector x =
        NumericVector::create(), // Size 0 vector
        bool bias = true,          // Set to true
        std::string method =
            "rcpp rules!") {       // Set string

    Rcpp::Rcout << "x size: " << x.size() << ", ";
    Rcpp::Rcout << "bias value: " << bias << ", ";
    Rcpp::Rcout << "method value: " << "."; 

}

/*** R
sample_defaults()                # all defaults
sample_defaults(1:5)             # supply x values

sample_defaults(bias = FALSE,    # supply bool
                method = "Rlang") # and string
*/
```

Note: In cpp, the default `bool` values are `true` and `false` whereas in R the valid types are `TRUE` or `FALSE`.

3.13. Can I use C++11, C++14, C++17, . . . with Rcpp. But of course. In a nutshell, this boils down to *what your compiler supports*, and also *what R supports*. We expanded a little on this in Rcpp Gallery article providing more detail. What follows in an abridged summary.

You can always *locally* set appropriate `PKG_CXXFLAGS` as an environment variable, or via `~/.R/Makevars`. You can also plugins and/or R support from `src/Makevars`:

- *C++11*: has been supported since early 2013 via a plugin selecting the language standard which is useful for `sourceCpp()` etc. For packages, R has supported it since R 3.1.0 which added the option to select the language standard via `CXX_STD = CXX11`. As of early 2017, over 120 packages on CRAN use this.
- *C++14*: has been supported since early 2016 via a plugin selecting the language standard which is useful for `sourceCpp()` etc. For packages, R supports it since R 3.4.0 adding the option to select the language standard via `CXX_STD = CXX14`.
- *C++17*: is itself more experimental now, but if you have a compiler supporting (at least parts of) it, you can use it via plugin (starting with Rcpp 0.12.10) for use via `sourceCpp()`, or via `PKG_CXXFLAGS` or other means to set compiler options. R support may be available at a later date.

3.14. How do I use it within (Python's) Conda setup?. In a comment to issue ticket #770 it is stated that running

```
conda install gxx_linux-64
```

helps within this environment as it installs the corresponding `x86_64-conda_cos6-linux-gnu-c++` compiler. Documentation for this and other systems is provided at this page.

3.15. Can I speed up compilation?. Somewhat. One option is to cache as much as possible via ccache by adding it to `~/.R/Makevars`.

Depending on what parts of Rcpp are being used, compilation speed can be increased by turning use of Modules off. Starting with version 1.0.3, the `RCPP_NO_MODULES` define can be used. It can be set in `src/Makevars` as an argument to `PKG_CXXFLAGS` (or one of the other C++ dialect options) as `-DRCPP_NO_MODULES`. This has the advantage of affecting *all* files in the package, including the auto-generated `RcppExports.cpp` where it might be trickier to set it manually.

Beyond modules, RTTI support can also be turned off. this implies turning Modules support off as well so. To select this approach, use the `RCPP_NO_RTTI` define.

4. Support

4.1. Is the API documented. You bet. We use doxygen to generate html, latex and man page documentation from the source. The html documentation is available for browsing, as a very large pdf file, and all three formats are also available a zip-archives: html, latex, and man.

4.2. Does it really work. We take quality seriously and have developed an extensive unit test suite to cover many possible uses of the **Rcpp** API.

We are always on the look for more coverage in our testing. Please let us know if something has not been tested enough.

4.3. Where can I ask further questions. The Rcpp-devel mailing list hosted at R-forge is by far the best place. You may also want to look at the list archives to see if your question has been asked before.

You can also use StackOverflow via its 'rcpp' tag.

4.4. Where can I read old questions and answers. The normal Rcpp-devel mailing list hosting at R-forge contains an archive, which can be searched via swish.

Alternatively, one can also use Mail-Archive on Rcpp-devel which offers web-based interfaces, including searching.

4.5. I like it. How can I help. We maintain a list of open issues in the Github repository. We welcome pull requests and suggest that code submissions come corresponding unit tests and, if applicable, documentation.

If you are willing to donate time and have skills in C++, let us know. If you are willing to donate money to sponsor improvements, let us know too.

You can also spread the word about **Rcpp**. There are many packages on CRAN that use C++, yet are not using **Rcpp**. You could blog about it, or get the word out otherwise.

Last but not least the Rcpp Gallery is open for user contributions.

4.6. I don't like it. How can I help. It is very generous of you to still want to help. Perhaps you can tell us what it is that you dislike. We are very open to *constructive* criticism.

4.7. Can I have commercial support for Rcpp. Sure you can. Just send us an email, and we will be happy to discuss the request.

4.8. I want to learn quickly. Do you provide training courses. Yes. Just send us an email.

4.9. Where is the code repository. From late 2008 to late 2013, we used the Subversion repository at R-Forge which contained **Rcpp** and a number of related packages. It still has the full history as well as number of support files.

We have since switched to a Git repository at Github for **Rcpp** (as well as for **RcppArmadillo** and **RcppEigen**).

5. Known Issues

Contained within this section is a list of known issues regarding **Rcpp**. The issues listed here are either not able to be fixed due to breaking application binary interface (ABI), would impact the ability to reproduce pre-existing results, or require significant work. Generally speaking, these issues come to light only when pushing the edge capabilities of **Rcpp**.

5.1. Rcpp changed the (const) object I passed by value. **Rcpp** objects are wrappers around the underlying R objects' SEXP, or S-expression. The SEXP is a pointer variable that holds the location of where the R object data has been stored (R Core Team, 2018b, Section 1.1). That is to say, the SEXP does *not* hold the actual data of the R object but merely a reference to where the data resides. When creating a new **Rcpp** object for an R object to enter C++, this object will use the same SEXP that powers the original R object if the types match otherwise a new SEXP must be created to be type safe. In essence, the underlying SEXP objects are passed by reference without explicit copies being made into C++. We refer to this arrangement as a *proxy model*.

As for the actual implementation, there are a few consequences of the proxy model. The foremost consequence within this paradigm is that pass by value is really a pass by reference. In essence, the distinction between the following two functions is only visual sugar:

```
void implicit_ref(NumericVector X);
void explicit_ref(NumericVector& X);
```

In particular, when one is passing by value what occurs is the instantiation of the new **Rcpp** object that uses the same SEXP for the R object. As a result, the **Rcpp** object is "linked" to the original R object. Thus, if an operation is performed on the **Rcpp** object, such as adding 1 to each element, the operation also updates the R object causing the change to be propagated to R's interactive environment.

```
#include<Rcpp.h>

// [[Rcpp::export]]
void implicit_ref(Rcpp::NumericVector X) {
  X = X + 1.0;
}

// [[Rcpp::export]]
void explicit_ref(Rcpp::NumericVector& X) {
  X = X + 1.0;
}
```

R use

```
a <- 1.5:4.5
b <- 1.5:4.5
implicit_ref(a)
a
#  [1] 2.5 3.5 4.5 5.5
explicit_ref(b)
b
#  [1] 2.5 3.5 4.5 5.5
```

There are two exceptions to this rule. The first exception is that a deep copy of the object can be made by explicit use of Rcpp:clone(). In this case, the cloned object has no link to the original R object. However, there is a time cost associated with this procedure as new memory must be allocated and the previous values must be copied over. The second exception, which was previously foreshadowed, is encountered when **Rcpp** and R object types do not match. One frequent example of this case is when the R object generated from seq() or a:b reports a class of "integer" while the **Rcpp** object is setup to receive the class of "numeric" as its object is set to NumericVector or NumericMatrix. In such cases, this would lead to a new SEXP object being created behind the scenes and, thus, there would *not* be a link between the **Rcpp** object and R object. So, any changes in C++ would not be propagated to R unless otherwise specified.

```
#include<Rcpp.h>

// [[Rcpp::export]]
void int_vec_type(Rcpp::IntegerVector X) {
  X = X + 1.0;
}
```

```
// [[Rcpp::export]]
void num_vec_type(Rcpp::NumericVector X) {
   X = X + 1.0;
}
```

R use:

```
a <- 1:5
b <- 1:5
class(a)
#  [1] "integer"
int_vec_type(a)
a # variable a changed as a side effect
#  [1] 2 3 4 5 6
num_vec_type(b)
b # b unchanged as copy was made for numeric
#  [1] 1 2 3 4 5
```

With this being said, there is one last area of contention with the proxy model: the keyword `const`. The `const` declaration indicates that an object is not allowed to be modified by any action. Due to the way the proxy model paradigm works, there is a way to "override" the `const` designation. Simply put, one can create a new **Rcpp** object without the `const` declaration from a pre-existing one. As a result, the new **Rcpp** object would be allowed to be modified by the compiler and, thus, modifying the initial `SEXP` object. Therefore, the initially secure R object would be altered. To illustrate this phenomenon, consider the following scenario:

```
#include <Rcpp.h>

// [[Rcpp::export]]
Rcpp::IntegerVector const_override_ex(
        const Rcpp::IntegerVector& X) {

  Rcpp::IntegerVector Y(X); // Create object
                            // from SEXP

  Y = Y * 2;                // Modify new object

  return Y;                 // Return new object
}
```

R use:

```
x <- 1:10    # an integer sequence
# returning an altered value
const_override_ex(x)
#   [1]  2  4  6  8 10 12 14 16 18 20
# but the original value is altered too!
x
#   [1]  2  4  6  8 10 12 14 16 18 20
```

So we see that with `SEXP` objects, the `const` declaration can be circumvented as it is really a pointer to the underlying R object.

5.2. Issues with implicit conversion from an Rcpp object to a scalar or other Rcpp object. Not all **Rcpp** expressions are directly compatible with `operator=`. Compability issues stem from many **Rcpp** objects and functions returning an intermediary result which requires an explicit conversion. In such cases, the user may need to assist the compiler with the conversion.

There are two ways to assist with the conversion. The first is to construct storage variable for a result, calculate the result, and then store a value into it. This is typically what is needed when working with `Character<Type>` and `String` in **Rcpp** due to the `Rcpp::internal::string_proxy` class. Within the following code snippet, the aforementioned approach is emphasized:

```
#include<Rcpp.h>

// [[Rcpp::export]]
std::string explicit_string_conv(
        Rcpp::CharacterVector X) {

    std::string s;  // define storage
    s = X[0];       // assign from CharacterVector

    return s;
}
```

If one were to use a direct allocation and assignment strategy, e.g. `std::string s = X[0]`, this would result in the compiler triggering a conversion error on *some* platforms. The error would be similar to:

```
error: no viable conversion from 'Proxy'
(aka 'string_proxy<16>') to 'std::string'
(aka 'basic_string<char, char_traits<char>,
allocator<char> >')
```

The second way to help the compiler is to use an explicit **Rcpp** type conversion function, if one were to exist. Examples of **Rcpp** type conversion functions include `as<T>()`, `.get()` for `cumsum()`, `is_true()` and `is_false()` for `any()` or `all()`.

5.3. Using `operator=` with a scalar replaced the object instead of filling element-wise. Assignment using the `operator=` with either `Vector` and `Matrix` classes will not elicit an element-wise fill. If you seek an element-wise fill, then use the `.fill()` member method to propagate a single value throughout the object. With this being said, the behavior of `operator=` differs for the `Vector` and `Matrix` classes.

The implementation of the `operator=` for the `Vector` class will replace the existing vector with the assigned value. This behavior is valid even if the assigned value is a scalar value such as 3.14 or 25 as the object is cast into the appropriate **Rcpp** object type. Therefore, if a `Vector` is initialized to have a length of 10 and a scalar is assigned via `operator=`, then the resulting `Vector` would have a length of 1. See the following code snippet for the aforementioned behavior.

```
#include<Rcpp.h>

// [[Rcpp::export]]
void vec_scalar_assign(int n, double fill_val) {
  Rcpp::NumericVector X(n);
  Rcpp::Rcout << "Value of Vector " <<
      "on Creation: " <<
      std::endl << X << std::endl;

  X = fill_val;

  Rcpp::Rcout << "Value of Vector " <<
      "after Assignment: " <<
```

```
    std::endl << X << std::endl;
}
```

R use:

```
vec_scalar_assign(5L, 3.14)
#  Value of Vector on Creation:
#  0 0 0 0 0
#  Value of Vector after Assignment:
#  3.14
```

Now, the `Matrix` class does not define its own `operator=` but instead uses the `Vector` class implementation. This leads to unexpected results while attempting to use the assignment operator with a scalar. In particular, the scalar will be coerced into a square `Matrix` and then assigned. For an example of this behavior, consider the following code:

```
#include<Rcpp.h>

// [[Rcpp::export]]
void mat_scalar_assign(int n, double fill_val) {
  Rcpp::NumericMatrix X(n, n);
  Rcpp::Rcout << "Value of Matrix " <<
      "on Creation: " <<
      std::endl << X << std::endl;

  X = fill_val;

  Rcpp::Rcout << "Value of Matrix " <<
      "after Assignment: " <<
      std::endl << X << std::endl;
}
```

R use:

```
mat_scalar_assign(2L, 3.0)
#  Value of Matrix on Creation:
#  0.00000 0.00000
#  0.00000 0.00000
#
#  Value of Matrix after Assignment:
#  0.00000 0.00000 0.00000
#  0.00000 0.00000 0.00000
#  0.00000 0.00000 0.00000
```

5.4. Long Vector support on Windows. Prior to R's 3.0.0, the largest vector one could obtain was at most $2^{31}-1$ elements. With the release of R's 3.0.0, long vector support was added to allow for largest vector possible to increase up to 2^{52} elements on x64 bit operating systems (c.f. Long Vectors help entry). Once this was established, support for long vectors within the **Rcpp** paradigm was introduced with **Rcpp** version 0.12.0 (c.f **Rcpp** 0.12.0 annoucement).

However, the requirement for using long vectors in **Rcpp** necessitates the presence of compiler support for the `R_xlen_t`, which is platform dependent on how `ptrdiff_t` is implemented. Unfortunately, this means that on the Windows platform the definition of `R_xlen_t` is of type `long` instead of `long long` when compiling under the C++98 specification. Therefore, to solve this issue one must compile under the specification for C++11 or later version.

There are three options to trigger compilation with C++11. The first – and most likely option to use – will be the plugin support offered by **Rcpp** attributes. This is engaged by adding `// [[Rcpp::plugins(cpp11)]]` to the top of the C++ script. For diagnostic and illustrativative purposes, consider the following code which checks to see if `R_xlen_t` is available on your platform:

```
#include <Rcpp.h>
// Force compilation mode to C++11
// [[Rcpp::plugins(cpp11)]]

// [[Rcpp::export]]
bool test_long_vector_support() {
#ifdef RCPP_HAS_LONG_LONG_TYPES
  return true;
#else
  return false;
#endif
}
```

R use:

```
test_long_vector_support()
#  [1] TRUE
```

The remaining two options are for users who have opted to embed **Rcpp** code within an R package. In particular, the second option requires adding `CXX_STD = CXX11` to a `Makevars` file found in the `/src` directory. Finally, the third option is to add `SystemRequirements:C++11` in the package's `DESCRIPTION` file.

Please note that the support for C++11 prior to R v3.3.0 on Windows is limited. Therefore, plan accordingly if the goal is to support older versions of R.

5.5. Sorting with STL on a `CharacterVector` produces problematic results. The Standard Template Library's (STL) `std::sort` algorithm performs adequately for the majority of **Rcpp** data types. The notable exception that makes what would otherwise be a universal quantifier into an existential quantifier is the `CharacterVector` data type. Chiefly, the issue with sorting strings is related to how the `CharacterVector` relies upon the use of `Rcpp::internal::string_proxy`. In particular, `Rcpp::internal::string_proxy` is *not* MoveAssignable since the left hand side of `operator=(const string_proxy \&rhs)` is *not* viewed as equivalent to the right hand side before the operation (ISO/IEC, 2011, p. 466, Table 22). This further complicates matters when using `CharacterVector` with `std::swap`, `std::move`, `std::copy` and their variants.

To avoid unwarranted pain with sorting, the preferred approach is to use the `.sort()` member function of **Rcpp** objects. The member function correctly applies the sorting procedure to **Rcpp** objects regardless of type. Though, sorting is slightly problematic due to locale as explained in the next entry. In the interim, the following code example illustrates the preferred approach alongside the problematic STL approach:

```
#include <Rcpp.h>

// [[Rcpp::export]]
Rcpp::CharacterVector preferred_sort(
        Rcpp::CharacterVector x) {

  Rcpp::CharacterVector y = Rcpp::clone(x);
  y.sort();

  return y;
```

```
}

// [[Rcpp::export]]
Rcpp::CharacterVector stl_sort(
        Rcpp::CharacterVector x) {

  Rcpp::CharacterVector y = Rcpp::clone(x);
  std::sort(y.begin(), y.end());

  return y;
}
```

R use:

```
set.seed(123)
(X <- sample(c(LETTERS[1:5], letters[1:6]), 11))
#   [1] "C" "f" "B" "a" "e" "E" "D" "d" "c" "A" "b"
preferred_sort(X)
#   [1] "A" "B" "C" "D" "E" "a" "b" "c" "d" "e" "f"
stl_sort(X)
#   [1] "f" "f" "f" "f" "f" "f" "f" "f" "f" "C" "f"
```

In closing, the results of using the STL approach do change depending on whether libc++ or libstdc++ standard library is used to compile the code. When debugging, this does make the issue particularly complex to sort out. Principally, compilation with libc++ and STL has been shown to yield the correct results. However, it is not wise to rely upon this library as a majority of code is compiled against libstdc++ as it more complete.

5.6. Lexicographic order of string sorting differs due to capitalization. Comparing strings within R hinges on the ability to process the locale or native-language environment of the string. In R, there is a function called Scollate that performs the comparison on locale. Unfortunately, this function has not been made publicly available and, thus, **Rcpp** does *not* have access to it within its implementation of StrCmp. As a result, strings that are sorted under the .sort() member function are ordered improperly. Specifically, if capitalization is present, then capitalized words are sorted together followed by the sorting of lowercase words instead of a mixture of capitalized and lowercase words. The issue is illustrated by the following code example:

```
#include <Rcpp.h>

// [[Rcpp::export]]
Rcpp::CharacterVector rcpp_sort(
        Rcpp::CharacterVector X) {
  X.sort();
  return X;
}
```

R use:

```
x <- c("B", "b", "c", "A", "a")
sort(x)
#  [1] "a" "A" "b" "B" "c"
rcpp_sort(x)
#  [1] "A" "B" "a" "b" "c"
```

5.7. Package building fails with 'symbols not found'. R 3.4.0 and later strongly encourage registering dynamically loadable symbols. In the stronger form (where .registration=TRUE is added to the useDynLib() statement in NAMESPACE), only registered symbols can be loaded. This is fully supported by Rcpp 0.12.12 and later, and the required code is added to src/RcppExports.cpp.

However, the transition from the previously generated file src/RcppExports.cpp to the new one may require running compileAttributes() twice (which does not happen when, *e.g.*, devtools is used). When Rcpp::compileAttributes() is called, it also calls tools::package_native_routine_registration_skeleton(), which crawls through usages of .Call() in the R/ source files of the package to figure out what routines need to be registered. If an older RcppExports.R file is discovered, its out-of-date symbol names get picked up, and registration rules for those symbols get written as well. This will register more symbols for the package than are actually defined, leading to an error. This point has been discussed at some length both in the GitHub issue tickes, on StackOverflow and elsewhere.

So if your autogenerated file fails, and a symbols not found error is reported by the linker, consider running compileAttributes() twice. Deleting R/RcppExports.R and src/RcppExports.cpp may also work.

5.8. Can we use exceptions and stop() across shared libraries?. Within limits, yes. Code that is generated via Rcpp Attributes (see Allaire *et al.* (2019) and Section~2.2.2) generally handles this correctly and gracefully via the try-catch layer it adds shielding the exception from propagating to another, separate dynamic library.

However, this mechanism relies on dynamic linking with the (system library) libgcc providing the C++ standard library (as well as on using the same C++ standard library across all compiled components). But this library is linked statically on Windows putting a limitation on the use of stop() from within Rcpp Modules (Eddelbuettel and François, 2019). Some more background on the linking requirement is in this SO question.

References

Allaire JJ, Eddelbuettel D, François R (2019). *Rcpp Attributes*. Vignette included in R package Rcpp, URL http://CRAN.R-Project.org/package=Rcpp.

Bates D, Eddelbuettel D (2013). "Fast and Elegant Numerical Linear Algebra Using the RcppEigen Package." *Journal of Statistical Software*, **52**(5), 1–24. URL http://www.jstatsoft.org/v52/i05/.

Eddelbuettel D (2013). *Seamless R and C++ Integration with Rcpp*. Use R! Springer, New York. ISBN 978-1-4614-6867-7.

Eddelbuettel D, Balamuta JJ (2017). "Extending R with C++: A Brief Introduction to Rcpp." *PeerJ Preprints*, **5**. doi: 10.7287/peerj.preprints.3188v1/.

Eddelbuettel D, Balamuta JJ (2018). "Extending R with C++: A Brief Introduction to Rcpp." *The American Statistician*, **72**(1). doi: 10.1080/00031305.2017.1375990.

Eddelbuettel D, François R (2011). "Rcpp: Seamless R and C++ Integration." *Journal of Statistical Software*, **40**(8), 1–18. URL http://www.jstatsoft.org/v40/i08/.

Eddelbuettel D, François R (2019). *Exposing C++ functions and classes with Rcpp modules*. Vignette included in R package Rcpp, URL http://CRAN.R-Project.org/package=Rcpp.

Eddelbuettel D, François R, Allaire J, Ushey K, Kou Q, Russel N, Chambers J, Bates D (2019a). *Rcpp: Seamless R and C++ Integration*. R package version 1.0.3, URL http://CRAN.R-Project.org/package=Rcpp.

Eddelbuettel D, François R, Bates D, Ni B (2019b). *RcppArmadillo: Rcpp integration for Armadillo templated linear algebra library*. R package version 0.9.800.1.0, URL http://CRAN.R-Project.org/package=RcppArmadillo.

Eddelbuettel D, Sanderson C (2014). "RcppArmadillo: Accelerating R with High-Performance C++ Linear Algebra." *Computational Statistics and Data

Analysis, **71**, 1054–1063. doi:10.1016/j.csda.2013.02.005. URL http://dx.doi.org/10.1016/j.csda.2013.02.005.

ISO/IEC (2011). "C++ 2011 Standard Document 14882:2011." ISO/IEC Standard Group for Information Technology / Programming Languages / C++. URL http://www.iso.org/iso/iso_catalogue/catalogue_tc/catalogue_detail.htm?csnumber=50372.

R Core Team (2018a). *R Installation and Administration*. R Foundation for Statistical Computing, Vienna, Austria. URL http://CRAN.R-Project.org/doc/manuals/R-admin.html.

R Core Team (2018b). *R internals*. R Foundation for Statistical Computing, Vienna, Austria. URL http://CRAN.R-Project.org/doc/manuals/R-ints.html.

R Core Team (2018c). *Writing R extensions*. R Foundation for Statistical Computing, Vienna, Austria. URL http://CRAN.R-Project.org/doc/manuals/R-exts.html.

Sanderson C (2010). "Armadillo: An open source C++ Algebra Library for Fast Prototyping and Computationally Intensive Experiments." *Technical report*, NICTA. URL http://arma.sf.net.

Sklyar O, Murdoch D, Smith M, Eddelbuettel D, François R (2018). *inline: Inline C, C++, Fortran function calls from R*. R package version 0.3.15, URL http://CRAN.R-Project.org/package=inline.
