# Supplementary material for: A biomathematical model of human erythropoiesis and iron metabolism
Source: Sci Rep. 2020 May 25;10:8602. doi: 10.1038/s41598-020-65313-5 (PMC7248076; doi:10.1038/s41598-020-65313-5)
Supplement: Supplementary file 5 — A biomathematical model of human erythropoiesis and iron metabolism: Simulation Model. [file 41598_2020_65313_MOESM5_ESM.zip › ErythroShort/Rcpp/doc/Rcpp-introduction.pdf]

Extending *R* with C++: A Brief Introduction to Rcpp

Dirk Eddelbuettel[a] **and James Joseph Balamuta**[b]

[a]Debian and R Projects; Chicago, IL, USA; edd@debian.org; [b]Depts of Informatics and Statistics, Univ. of Illinois at Urbana-Champaign; Champaign, IL, USA; balamut2@illinois.edu

This version was compiled on November 8, 2019

***R* has always provided an application programming interface (API) for extensions. Based on the *C* language, it uses a number of macros and other low-level constructs to exchange data structures between the *R* process and any dynamically-loaded component modules authors added to it. With the introduction of the *Rcpp* package, and its later refinements, this process has become considerably easier yet also more robust. By now, *Rcpp* has become the most popular extension mechanism for *R*. This article introduces *Rcpp*, and illustrates with several examples how the *Rcpp Attributes* mechanism in particular eases the transition of objects between *R* and *C++* code.**

applications and case studies | statistical computing | computationally intensive methods | simulation

Introduction

The *R* language and environment (R Core Team, 2018a) has established itself as both an increasingly dominant facility for data analysis, and the *lingua franca* for statistical computing in both research and application settings.

Since the beginning, and as we argue below, "by design", the *R* system has always provided an application programming interface (API) suitable for extending *R* with code written in *C* or *Fortran*. Being implemented chiefly in *R* and *C* (with a generous sprinkling of *Fortran* for well-established numerical subroutines), *R* has always been extensible via a *C* interface. Both the actual implementation and the *C* interface use a number of macros and other low-level constructs to exchange data structures between the *R* process and any dynamically-loaded component modules authors added to it.

A *C* interface will generally also be accessible to other languages. Particularly noteworthy here is the *C++* language, developed originally as a 'better *C*', which is by its design very interoperable with *C*. And with the introduction of the *Rcpp* package (Eddelbuettel and François, 2011; Eddelbuettel, 2013; Eddelbuettel *et al.*, 2019a), and its later refinements, this process of extending *R* has become considerably easier yet also more robust. To date, *Rcpp* has become the most popular extension system for *R*. This article introduces *Rcpp*, and illustrates with several examples how the *Rcpp Attributes* mechanism (Allaire *et al.*, 2019) in particular eases the transition of objects between *R* and *C++* code.

Background. Chambers (2008, p. 3) provides a very thorough discussion of desirable traits for a system designed to *program with data*, and the *R* system in particular. Two key themes motivate the introductory discussion. First, the *Mission* is to aid exploration in order to provide the best platform to analyse data: "to boldly go where no one has gone before." Second, the *Prime Directive* is that the software systems we build must be *trustworthy*: "the many computational steps between original data source and displayed result must all be trustful." The remainder of the book then discusses *R*, leading to two final chapters on interfaces.

Chambers (2016, p. 4) builds and expands on this theme. Two core facets of what "makes" *R* are carried over from the previous book. The first states what *R* is composed of: *Everything that exists in R is an object*. The second states how these objects are created or altered: *Everything that happens in R is a function call*. A third statement is now added: *Interfaces to other software are part of R*.

This last addition is profound. If and when suitable and performant software for a task exists, it is in fact desirable to have a (preferably also perfomant) interface to this software from *R*. Chambers (2016) discusses several possible approaches for simpler interfaces and illustrates them with reference implementations to both *Python* and *Julia*. However, the most performant interface for *R* is provided at the subroutine level, and rather than discussing the older *C* interface for *R*, Chambers (2016) prefers to discuss *Rcpp*. This article follows the same school of thought and aims to introduce *Rcpp* to analysts and data scientists, aiming to enable them to use—and create— further *interfaces* for *R* which aid the *mission* while staying true to the *prime directive*. Adding interfaces in such a way is in fact a natural progression from the earliest designs for its predecessor *S* which was after all designed to provide a more useable 'interface' to underlying routines in *Fortran*.

The rest of the paper is structured as follows. We start by discussing possible first steps, chiefly to validate correct installations. This is followed by an introduction to simple *C++* functions, comparison to the *C* API, a discussion of packaging with *Rcpp* and a linear algebra example. The appendix contains some empirical illustrations of the adoption of *Rcpp*.

First Steps with *Rcpp*

Rcpp is a CRAN package and can be installed by using `install.packages('Rcpp')` just like any other *R* package. On some operating systems this will download *pre-compiled* binary packages; on others an installation from source will be attempted. But *Rcpp* is a little different from many standard *R* packages in one important aspect: it helps the user to *write C(++) programs more easily*. The key aspect to note here is *C++* programs: to operate, *Rcpp* needs not only *R* but also an additional *toolchain* of a compiler, linker and more in order to be able to create *binary* object code extending *R*.

We note that this requirement is no different from what is needed with base *R* when compilation of extensions is attempted. How to achieve this using only base *R* is described in some detail in the *Writing R Extensions* manual (R Core Team, 2018b) that is included with *R*. As for the toolchain requirements, on Linux and macOS, all required components are likely to be present. The macOS can offer additional challenges as toolchain elements can be obtained in different ways. Some of these are addressed in the *Rcpp FAQ* (Eddelbuettel and François, 2019a) in sections 2.10 and 2.16. On Windows, users will have to install the Rtools kit provided by R Core available at https://cran.r-project.org/bin/windows/Rtools/. Details of these installation steps are beyond the scope of this paper. However, many external resources exist that provide detailed installation guides for *R* toolschains in Windows and macOS.

As a first step, and chiefly to establish that the toolchain is set up correctly, consider a minimal use case such as the following:

```
library("Rcpp")
evalCpp("2 + 2")
#  [1] 4
```

Here the *Rcpp* package is loaded first via the `library()` function. Next, we deploy one of its simplest functions, `evalCpp()`, which is described in the *Rcpp Attributes* vignette (Allaire *et al.*, 2019). It takes the first (and often only) argument—a character object—and evaluates it as a minimal *C++* expression. The value assignment and return are implicit, as is the addition of a trailing semicolon and more. In fact, `evalCpp()` surrounds the expression with the required 'glue' to make it a minimal source file which can be compiled, linked and loaded. The exact details behind this process are available in-depth when the `verbose` option of the function is set. If everything is set up correctly, the newly-created *R* function will be returned.

While such a simple expression is not interesting in itself, it serves a useful purpose here to unequivocally establish whether *Rcpp* is correctly set up. Having accomplished that, we can proceed to the next step of creating simple functions.

A first *C++* function using *Rcpp*

As a first example, consider the determination of whether a number is odd or even. The default practice is to use modular arithmetic to check if a remainder exists under x mod 2. Within *R*, this can be implemented as follows:

```
isOddR <- function(num = 10L) {
   result <- (num %% 2L == 1L)
   return(result)
}
isOddR(42L)
#  [1] FALSE
```

The operator `%%` implements the mod operation in *R*. For the default (integer) argument of ten used in the example, 10 mod 2 results in zero, which is then mapped to `FALSE` in the context of a logical expression.

Translating this implementation into *C++*, several small details have to be considered. First and foremost, as *C++* is a *statically-typed language*, there needs to be additional (compile-time) information provided for each of the variables. Specifically, a *type*, *i.e.* the kind of storage used by a variable must be explicitly defined. Typed languages generally offer benefits in terms of both correctness (as it is harder to accidentally assign to an ill-matched type) and performance (as the compiler can optimize code based on the storage and cpu characteristics). Here we have an `int` argument, but return a logical, or `bool` for short. Two more smaller differences are that each statement within the body must be concluded with a semicolon, and that `return` does not require parentheses around its argument. A graphical breakdown of all aspects of a corresponding *C++* function is given in Figure 1.

When using *Rcpp*, such *C++* functions can be directly embedded and compiled in an *R* script file through the use of the `cppFunction()` provided by *Rcpp Attributes* (Allaire *et al.*, 2019). The first parameter of the function accepts string input that represents the *C++* code. Upon calling the `cppFunction()`, and similarly to the earlier example involving `evalCpp()`, the *C++* code is both *compiled* and *linked*, and then *imported* into *R* under the name of the function supplied (*e.g.* here `isOddCpp()`).

```
library("Rcpp")
cppFunction("
bool isOddCpp(int num = 10) {
   bool result = (num % 2 == 1);
   return result;
}")
isOddCpp(42L)
#  [1] FALSE
```

Extending *R* via its *C* API

Let us first consider the case of 'standard *R*', *i.e.* the API as defined in the core *R* documentation. Extending *R* with routines written using the *C* language requires the use of internal macros and functions documented in Chapter 5 of *Writing R Extensions* (R Core Team, 2018b).

```
#include <R.h>
#include <Rinternals.h>

SEXP convolve2(SEXP a, SEXP b) {
    int na, nb, nab;
    double *xa, *xb, *xab;
    SEXP ab;

    a = PROTECT(coerceVector(a, REALSXP));
    b = PROTECT(coerceVector(b, REALSXP));
    na = length(a); nb = length(b);
    nab = na + nb - 1;
    ab = PROTECT(allocVector(REALSXP, nab));
    xa = REAL(a); xb = REAL(b); xab = REAL(ab);
    for (int i = 0; i < nab; i++)
        xab[i] = 0.0;
    for (int i = 0; i < na; i++)
        for (int j = 0; j < nb; j++)
            xab[i + j] += xa[i] * xb[j];
    UNPROTECT(3);
    return ab;
}
```

This function computes a *convolution* of two vectors supplied on input, a and b, which is defined to be $ab_{k+1} = \sum_{i+j==k} a_i \cdot b_j$. Before computing the convolution (which is really just the three lines involving two nested for loops with indices i and j), a total of ten lines of mere housekeeping are required. Vectors a and b are coerced to `double`, and a results vector `ab` is allocated. This expression involves three calls to the `PROTECT` macro for which a *precisely* matching `UNPROTECT(3)` is required as part of the interfacing of internal memory allocation. The vectors are accessed through pointer equivalents `xa`, `xb` and `xab`; and the latter has to be explicitly zeroed prior to the convolution calculation involving incremental summary at index $i + j$.

Extending *R* via the *C++* API of *Rcpp*

Using the idioms of *Rcpp*, the above example can be written in a much more compact fashion—leading to code that is simpler to read and maintain.

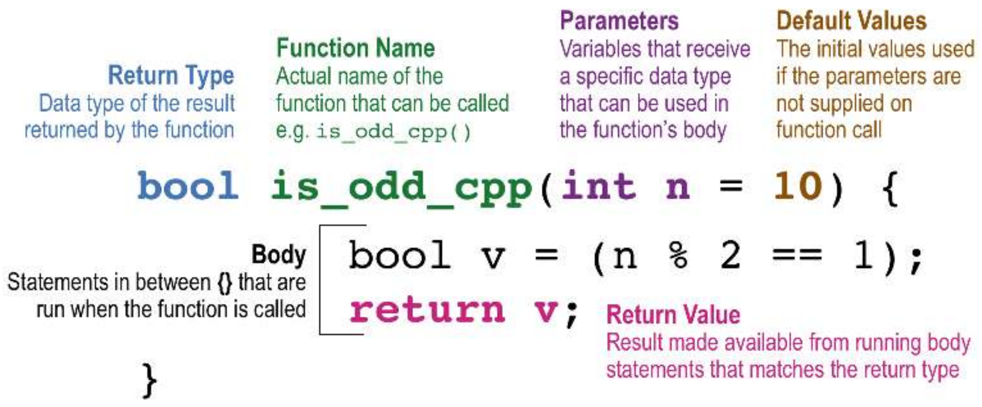

Fig. 1. Graphical annotation of the `is_odd_cpp` function.

```
#include "Rcpp.h"
using namespace Rcpp;

// [[Rcpp::export]]
NumericVector
convolve_cpp(const NumericVector& a,
             const NumericVector& b) {

    // Declare loop counters, and vector sizes
    int i, j,
        na = a.size(), nb = b.size(),
        nab = na + nb - 1;

    // Create vector filled with 0
    NumericVector ab(nab);

    // Crux of the algorithm
    for(i = 0; i < na; i++) {
        for(j = 0; j < nb; j++) {
            ab[i + j] += a[i] * b[j];
        }
    }

    // Return result
    return ab;
}
```

To deploy such code from within an *R* script or session, first save it into a new file—which could be called **convolve.cpp**—in either the working directory, a temporary directoy or a project directory. Then from within the *R* session, use `Rcpp::sourceCpp("convolve.cpp")` (possibly using a path as well as the filename). This not only compiles, links and loads the code within the external file but also adds the necessary "glue" to make the *Rcpp* function available in the *R* environment. Once the code is compiled and linked, call the newly-created `convolve_cpp()` function with the appropriate parameters as done in previous examples.

What is notable about the *Rcpp* version is that it has no `PROTECT` or `UNPROTECT` which not only frees the programmer from a tedious (and error-prone) step but more importantly also shows that memory management can be handled automatically. The result vector is already initialized at zero as well, reducing the entire function to just the three lines for the two nested loops, plus some variable declarations and the `return` statement. The resulting code is shorter, easier to read, comprehend and maintain. Furthermore, the *Rcpp* code is more similar to traditional *R* code, which reduces the barrier of entry.

Data Driven Performance Decisions with *Rcpp*

When beginning to implement an idea, more so an algorithm, there are many ways one is able to correctly implement it. Prior to the routine being used in production, two questions must be asked:

1. Does the implementation produce the *correct* results?
2. What implementation of the routine is the *best*?

The first question is subject to a binary pass-fail unit test verification while the latter question is where the details of an implementation are scrutinized to extract maximal efficiency from the routine. The quality of the *best* routine follows first and foremost from its correctness. To that end, *R* offers many different unit testing frameworks such as **RUnit** by Burger *et al.* (2015), which is used to construct *Rcpp*'s 1385+ unit tests, and **testthat** by Wickham (2011). Only when correctness is achieved is it wise to begin the procedure of optimizing the efficiency of the routine and, in turn, selecting the best routine.

Optimization of an algorithm involves performing a quantitative analysis of the routine's properties. There are two main approaches to analyzing the behavior of a routine: theoretical analysis[1] or an empirical examination using profiling tools.[2] Typically, the latter option is more prominently used as the routine's theoretical properties are derived prior to an implementation being started. Often the main concern regarding an implementation in *R* relates to the speed of the algorithm as it impacts how quickly analyses can be done and reports can be provided to decision makers. Coincidentally, the speed of code is one of the key governing use cases of *Rcpp*. Profiling *R* code will reveal shortcomings related to loops, *e.g.* `for`, `while`, and `repeat`; conditional statements, *e.g.* `if-else`

[1] Theoretical analysis is often directed to describing the limiting behavior of a function through asymptotic notation, commonly referred to as Big O and denoted as $\mathcal{O}(\cdot)$.

[2] Within base *R*, profiling can be activated by `utils::Rprof()` for individual command timing information, `utils::Rprofmem()` for memory information, and `System.time({})` for a quick overall execution timing. Additional profiling *R* packages such as **profvis** by Chang and Luraschi (2017), **Rperform** by Tandon and Hocking (2015), and benchmarking packages have extended the ability to analyze performance.

`if-else` and `switch`; and recursive functions, *i.e.* a function written in terms of itself such that the problem is broken down on each call in a reduced state until an answer can be obtained. In contrast, the overhead for such operations is significantly less in *C++*. Thus, critical components of a given routine should be written in *Rcpp* to capture maximal efficiency.

Returning to the second question, to decide which implementation works the best, one needs to employ a benchmark to obtain *quantifiable results*. Benchmarks are an ideal way to quantify how well a method performs because they have the ability to show the amount of time the code has been running and where bottlenecks exist within functions. This does not imply that benchmarks are completely infallible as user error can influence the end results. For example, if a user decides to benchmark code in one *R* session and in another session performs a heavy computation, then the benchmark will be biased (if "wall clock" is measured).

There are different levels of magnification that a benchmark can provide. For a more macro analysis, one should benchmark data using `benchmark(test = func(), test2 = func2())`, a function from the **rbenchmark** *R* package by Kusnierczyk (2012). This form of benchmarking will be used when the computation is more intensive. The motivating example `isOdd()` (which is only able to accept a single `integer`) warrants a much more microscopic timing comparison. In cases such as this, the objective is to obtain precise results in the amount of nanoseconds elapsed. Using the `microbenchmark` function from the **microbenchmark** *R* package by Mersmann (2015) is more helpful to obtain timing information. To perform the benchmark:

```
library("microbenchmark")
results <- microbenchmark(isOddR   = isOddR(12L),
                          isOddCpp = isOddCpp(12L))
print(summary(results)[, c(1:7)],digits=1)
```

By looking at the summary of 100 evaluations, we note that the *Rcpp* function performed better than the equivalent in *R* by achieving a lower run time on average. The lower run time in this part is not necessarily critical as the difference is nanoseconds on a trivial computation. However, each section of code does contribute to a faster overall runtime.

Random Numbers within *Rcpp*: An Example of *Rcpp Sugar*

Rcpp connects *R* with *C++*. Only the former is vectorized: *C++* is not. *Rcpp Sugar*, however, provides a convenient way to work with high-performing *C++* functions in a similar way to how *R* offers vectorized operations. The *Rcpp Sugar* vignette (Eddelbuettel and François, 2019b) details these, as well as many more functions directly accessible to *Rcpp* in a way that should feel familiar to *R* users. Some examples of *Rcpp Sugar* functions include special math functions like gamma and beta, statistical distributions and random number generation.

We will illustrate a case of random number generation. Consider drawing one or more $N(0,1)$-distributed random variables. The very simplest case can just use `evalCpp()`:

```
evalCpp("R::rnorm(0, 1)")
#  [1] 0.073716
```

By setting a seed, we can make this reproducible:

```
set.seed(123)
evalCpp("R::rnorm(0, 1)")
#  [1] -0.56048
```

One important aspect of the behind-the-scenes code generation for the single expression (as well as all code created via *Rcpp Attributes*) is the automatic preservation of the state of the random nunber generators in *R*. This means that from a given seed, we will receive *identical* draws of random numbers whether we access them from *R* or via *C++* code accessing the same generators (via the *Rcpp* interfaces). To illustrate, the same number is drawn via *R* code after resetting the seed:

```
set.seed(123)
# Implicit mean of 0, sd of 1
rnorm(1)
#  [1] -0.56048
```

We can make the *Rcpp Sugar* function `rnorm()` accessible from *R* in the same way to return a vector of values:

```
set.seed(123)
evalCpp("Rcpp::rnorm(3)")
#  [1] -0.56048 -0.23018  1.55871
```

Note that we use the `Rcpp::` namespace explicitly here to contrast the vectorised `Rcpp::rnorm()` with the scalar `R::rnorm()` also provided as a convenience wrapper for the *C* API of *R*.

And as expected, this too replicates from *R* as the very same generators are used in both cases along with consistent handling of generator state permitting to alternate:

```
set.seed(123)
rnorm(3)
#  [1] -0.56048 -0.23018  1.55871
```

Translating Code from *R* into *Rcpp*: Bootstrap Example

Statistical inference relied primarily upon asymptotic theory until Efron (1979) proposed the bootstrap. Bootstrapping is known to be computationally intensive due to the need to use loops. Thus, it is an ideal candidate to use as an example. Before starting to write *C++* code using *Rcpp* , prototype the code in *R*.

```
# Function declaration
bootstrap_r <- function(ds, B = 1000) {

  # Preallocate storage for statistics
  boot_stat <- matrix(NA, nrow = B, ncol = 2)

  # Number of observations
  n <- length(ds)

  # Perform bootstrap
  for(i in seq_len(B)) {
     # Sample initial data
     gen_data <- ds[ sample(n, n, replace=TRUE) ]
     # Calculate sample data mean and SD
     boot_stat[i,] <- c(mean(gen_data),
                        sd(gen_data))
  }

```

```
  # Return bootstrap result
  return(boot_stat)
}
```

Before continuing, check that the initial prototype *R* code works. To do so, write a short *R* script. Note the use of `set.seed()` to ensure reproducible draws.

```
# Set seed to generate data
set.seed(512)
# Generate data
initdata <- rnorm(1000, mean = 21, sd = 10)
# Set a new _different_ seed for bootstrapping
set.seed(883)
# Perform bootstrap
result_r <- bootstrap_r(initdata)
```

Figure 2 shows that the bootstrap procedure worked well!

With reassurances that the method to be implemented within *Rcpp* works appropriately in *R*, proceed to translating the code into *Rcpp*. As indicated previously, there are many convergences between *Rcpp* syntax and base *R* via *Rcpp* Sugar.

```
#include <Rcpp.h>

// Function declaration with export tag
// [[Rcpp::export]]
Rcpp::NumericMatrix
bootstrap_cpp(Rcpp::NumericVector ds,
              int B = 1000) {

  // Preallocate storage for statistics
  Rcpp::NumericMatrix boot_stat(B, 2);

  // Number of observations
  int n = ds.size();

  // Perform bootstrap
  for(int i = 0; i < B; i++) {
    // Sample initial data
    Rcpp::NumericVector gen_data =
        ds[ floor(Rcpp::runif(n, 0, n)) ];
    // Calculate sample mean and std dev
    boot_stat(i, 0) = mean(gen_data);
    boot_stat(i, 1) = sd(gen_data);
  }

  // Return bootstrap results
  return boot_stat;
}
```

In the *Rcpp* version of the bootstrap function, there are a few additional changes that occurred during the translation. In particular, the use of `Rcpp::runif(n, 0, n)` enclosed by `floor()`, which rounds down to the nearest integer, in place of `sample(n, n, replace = TRUE)` to sample row ids. This is an equivalent substitution since equal weight is being placed upon all row ids and replacement is allowed.[3] Note that the upper bound of the interval, `n`, will never be reached. While this may seem flawed, it is important to note that vectors and matrices in *C++* use a zero-based indexing system, meaning that they begin at 0 instead of 1 and go up to $n-1$ instead of n, which is unlike *R*'s system. Thus, an out of bounds error would be triggered if `n` was used as that point does *not* exist within the data structure. The application of this logic can be seen in the span the `for` loop takes in *C++* when compared to *R*. Another syntactical change is the use of `()` in place of `[]` while accessing the matrix. This change is due to the governance of *C++* and its comma operator making it impossible to place multiple indices inside the square brackets.

To validate that the translation was successful, first run the *C++* function under the *same* data and seed as was given for the *R* function.

```
# Use the same seed use in R and C++
set.seed(883)
# Perform bootstrap with C++ function
result_cpp <- bootstrap_cpp(initdata)
```

Next, check the output between the functions using *R* 's `all.equal()` function that allows for an ε-neighborhood around a number.

```
# Compare output
all.equal(result_r, result_cpp)
#  [1] "Mean relative difference: 0.019931"
```

Lastly, make sure to benchmark the newly translated *Rcpp* function against the *R* implementation. As stated earlier, data is paramount to making a decision related to which function to use in an analysis or package.

```
library(rbenchmark)

benchmark(r = bootstrap_r(initdata),
          cpp = bootstrap_cpp(initdata))[, 1:4]
#    test replications elapsed relative
#  2  cpp          100   1.252    1.000
#  1    r          100   4.085    3.263
```

Using *Rcpp* as an Interface to External Libraries: Exploring Linear Algebra Extensions

Many of the previously illustrated *Rcpp* examples were directed primarily to show the gains in computational efficiency that are possible by implementing code directly in *C++*; however, this is only one potential application of *Rcpp*. Perhaps one of the most understated features of *Rcpp* is its ability to enable Chambers (2016)'s third statement of *Interfaces to other software are part of R*. In particular, *Rcpp* is designed to facilitate interfacing libraries written in *C++* or *C* to *R*. Hence, if there is a specific feature within a *C++* or *C* library, then one can create a bridge to it using *Rcpp* to enable it from within *R*.

An example is the use of *C++* matrix algebra libraries like **Armadillo** (Sanderson, 2010) or **Eigen** (Guennebaud *et al.*, 2012). By outsourcing complex linear algebra operations to matrix libraries, the need to directly call functions within **Linear Algebra PACKage (LAPACK)** (Anderson *et al.*, 1999) is negated. Moreover, the *Rcpp* design allows for seamless transfer between object types by using automatic converters governed by `wrap()`, *C++* to *R* , and `as<T>()`, *R* to *C++* with the `T` indicating the type of object being cast into. These two helper functions provide a non-invasive way to work with an external object. Thus, a further benefit to using

[3] For more flexibility in sampling see Christian Gunning's Sample extension for **RcppArmadillo** and Rcpp Gallery: Using the **RcppArmadillo**-based Implementation of R's sample() or consider using the `Rcpp::sample()` sugar function added in 0.12.9 by Nathan Russell.

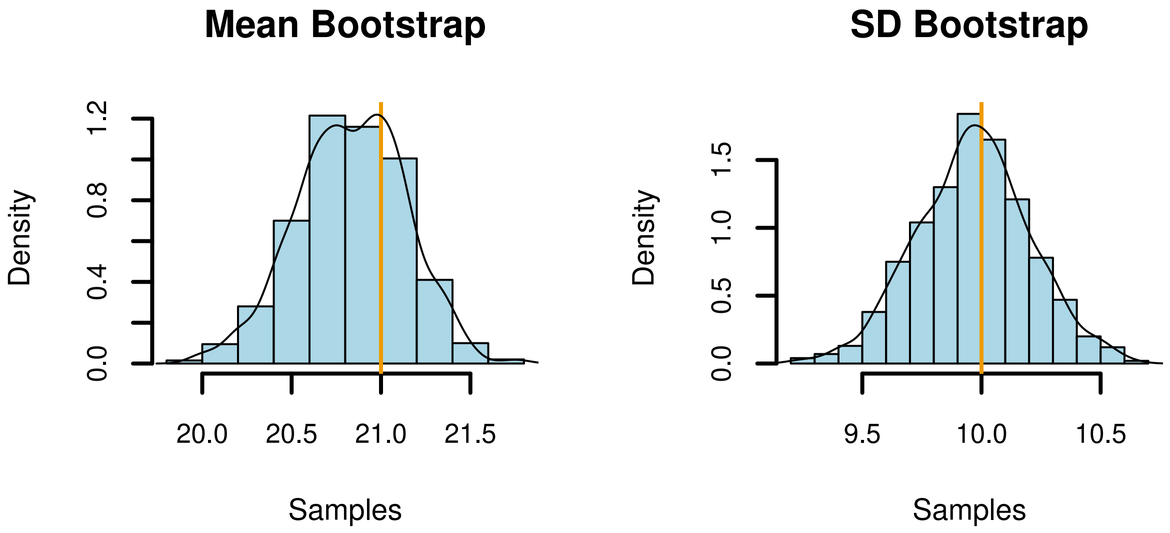

Fig. 2. Results of the bootstrapping procedure for sample mean and variance.

external *C++* libraries is the ability to have a portable code base that can be implemented within a standalone *C++* program or within another computational language.

Compute RNG draws from a multivariate Normal. A common application in statistical computing is simulating from a multivariate normal distribution. The algorithm relies on a linear transformation of the standard Normal distribution. Letting $\boldsymbol{Y}_{m\times 1} = \boldsymbol{A}_{m\times n}\boldsymbol{Z}_{n\times 1} + \boldsymbol{b}_{m\times 1}$, where $\boldsymbol{A}$ is a $m \times n$ matrix, $\boldsymbol{b} \in \mathbb{R}^m$, $\boldsymbol{Z} \sim N(\boldsymbol{0}_n, \boldsymbol{I}_n)$, and $\boldsymbol{I}_n$ is the identity matrix, then $\boldsymbol{Y} \sim N_m\left(\boldsymbol{\mu} = \boldsymbol{b}, \boldsymbol{\Sigma} = \boldsymbol{A}\boldsymbol{A}^T\right)$. To obtain the matrix $\boldsymbol{A}$ from $\boldsymbol{\Sigma}$, either a Cholesky or Eigen decomposition is required. As noted in Venables and Ripley (2002), the Eigen decomposition is more stable in addition to being more computationally demanding compared to the Cholesky decomposition. For simplicity and speed, we have opted to implement the sampling procedure using a Cholesky decomposition. Regardless, there is a need to involve one of the above matrix libraries to make the sampling viable in *C++*.

Here, we demonstrate how to take advantage of the *Armadillo* linear algebra template classes (Sanderson and Curtin, 2016) via the **RcppArmadillo** package (Eddelbuettel and Sanderson, 2014; Eddelbuettel *et al.*, 2019b). Prior to running this example, the **RcppArmadillo** package must be installed using `install.packages('RcppArmadillo')`.[4] One important caveat when using additional packages within the *Rcpp* ecosystem is the correct header file may not be `Rcpp.h`. In a majority of cases, the additional package ships a dedicated header (as *e.g.* `RcppArmadillo.h` here) which not only declares data structures from both systems, but may also add complementary integration and conversion routines. It typically needs to be listed in an `include` statement along with a `depends()` attribute to tell *R* where to find the additional header files:

```
// Use the RcppArmadillo package
// Requires different header file from Rcpp.h
#include <RcppArmadillo.h>
// [[Rcpp::depends(RcppArmadillo)]]
```

With this in mind, sampling from a multivariate normal distribution can be obtained in a straightforward manner. Using only *Armadillo* data types and values:

```
#include <RcppArmadillo.h>
// [[Rcpp::depends(RcppArmadillo)]]

// Sample N x P observations from a Standard
// Multivariate Normal given N observations, a
// vector  of P means, and a P x P cov matrix
// [[Rcpp::export]]
arma::mat rmvnorm(int n,
                  const arma::vec& mu,
                  const arma::mat& Sigma) {
    unsigned int p = Sigma.n_cols;

    // First draw N x P values from a N(0,1)
    Rcpp::NumericVector draw = Rcpp::rnorm(n*p);

    // Instantiate an Armadillo matrix with the
    // drawn values using advanced constructor
    // to reuse allocated memory
    arma::mat Z = arma::mat(draw.begin(), n, p,
                            false, true);

    // Simpler, less performant alternative
    // arma::mat Z = Rcpp::as<arma::mat>(draw);

    // Generate a sample from the Transformed
    // Multivariate Normal
```

[4] macOS users may encounter '-lgfortran' and '-lquadmath' errors on compilations with this package if the development environment is not appropriately set up. Section 2.16 of the Rcpp FAQ provides details regarding the necessary 'gfortran' binaries.

```
    arma::mat Y = arma::repmat(mu, 1, n).t() +
        Z * arma::chol(Sigma);

    return Y;
}
```

As a result of using a random number generation (RNG), there is an additional requirement to ensure reproducible results: the necessity to explicitly set a seed (as shown above). Because of the (programmatic) interface provided by *R* to its own RNGs, this setting of the seed has to occur at the *R* level via the `set.seed()` function as no (public) interface is provided by the *R* header files.

Faster linear model fits. As a second example, consider the problem of estimating a common linear model repeatedly. One use case might be the simulation of size and power of standard tests. Many users of *R* would default to using `lm()`, however, the overhead associated with this function greatly impacts speed with which an estimate can be obtained. Another approach would be to take the base *R* function `lm.fit()`, which is called by `lm()`, to compute estimated $\hat{\beta}$ in just about the fastest time possible. However, this approach is also not viable as it does not report the estimated standard errors. As a result, we cannot use any default *R* functions in the context of simulating finite sample population effects on inference.

One alternative is provided by the `fastLm()` function in **RcppArmadillo** (Eddelbuettel *et al.*, 2019b).

```
#include <RcppArmadillo.h>
// [[Rcpp::depends(RcppArmadillo)]]

// Compute coefficients and their standard error
// during multiple linear regression given a
// design matrix X containing N observations with
// P regressors and a vector y containing of
// N responses
// [[Rcpp::export]]
Rcpp::List fastLm(const arma::mat& X,
                  const arma::colvec& y) {
    // Dimension information
    int n = X.n_rows, p = X.n_cols;
    // Fit model y ~ X
    arma::colvec coef = arma::solve(X, y);
    // Compute the residuals
    arma::colvec res  = y - X*coef;
    // Estimated variance of the random error
    double s2 =
        std::inner_product(res.begin(), res.end(),
                           res.begin(), 0.0)
                           / (n - p);

    // Standard error matrix of coefficients
    arma::colvec std_err = arma::sqrt(s2 *
        arma::diagvec(arma::pinv(X.t()*X)));

    // Create named list with the above quantities
    return Rcpp::List::create(
        Rcpp::Named("coefficients") = coef,
        Rcpp::Named("stderr")       = std_err,
        Rcpp::Named("df.residual")  = n - p    );
}
```

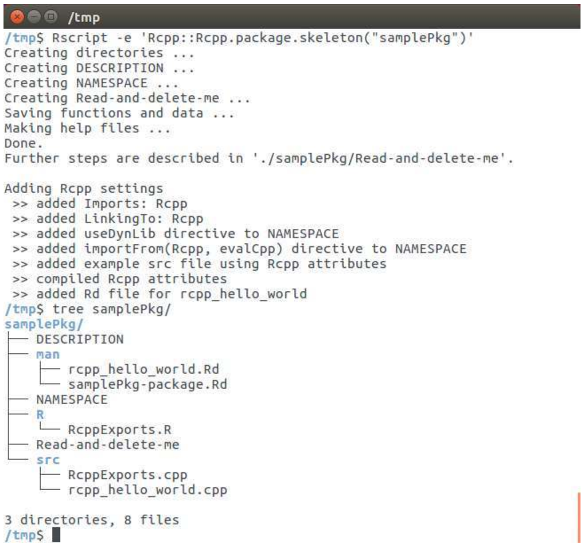

Fig. 3. Graphical annotation of the `is_odd_cpp` function.

The interface is very simple: a matrix $X_{n\times p}$ of regressors, and a dependent variable $y_{n\times 1}$ as a vector. We invoke the standard Armadillo function `solve()` to fit the model `y ~ X`.[5] We then compute residuals, and extract the (appropriately scaled) diagonal of the covariance matrix, also taking its square root, in order to return both estimates $\hat{\beta}$ and $\hat{\sigma}$.

Rcpp in Packages

Once a project containing compiled code has matured to the point of sharing it with collaborators[6] or using it within a parallel computing environments, the ideal way forward is to embed the code within an *R* package. Not only does an *R* package provide a way to automatically compile source code, but also enables the use of the *R* help system to document how the written functions should be used. As a further benefit, the package format enables the use of unit tests to ensure that the functions are producing the correct output. Lastly, having a package provides the option of uploading to a repository such as CRAN for wider dissemination.

To facilitate package building, *Rcpp* provides a function `Rcpp.package.skeleton()` that is modeled after the base *R* function `package.skeleton()`. This function automates the creation of a skeleton package appropriate for distributing *Rcpp*:

```
library("Rcpp")
Rcpp.package.skeleton("samplePkg")
```

This shows how distinct directories `man`, `R`, `src` are created for, respectively, the help pages, files with *R* code and files with *C++* code. Generally speaking, all compiled code, be it from *C*, *C++* or *Fortran* sources, should be placed within the `src/` directory.

Alternatively, one can achieve similar results to using `Rcpp.package.skeleton()` by using a feature of the RStudio IDE.

[5] We should note that this will use the standard **LAPACK** functionality via Armadillo whereas R uses an internal refinement of **LINPACK** (Dongarra *et al.*, 1979) via pivoting, rendering the operation numerically more stable. That is an important robustness aspect—though common datasets on current hardware almost never lead to actual differences. That said, if in doubt, stick with the R implementation. What is shown here is mostly for exposition of the principles.

[6] It is sometimes said that every project has two collaborators: self, and future self. Packaging code is *best practices* even for code not intended for public uploading.

Specifically, while creating a new package project there is an option to select the type of package by engaging a dropdown menu to select "Package w/ Rcpp" in RStudio versions prior to v1.1.0. In RStudio versions later than v1.1.0, support for package templates has been added allowing users to directly create *Rcpp*-based packages that use Eigen or Armadillo.

Lastly, one more option exists for users who are familiar with the **devtools** *R* package. To create the *R* package skeleton use `devtools::create("samplePkg")`. From here, part of the structure required by *Rcpp* can be added by using `devtools::use_rcpp()`. The remaining aspects needed by *Rcpp* must be manually copied from the roxygen tags written to console and pasted into one of the package's *R* files to successfully incorporate the dynamic library and link to *Rcpp*'s headers.

All of these methods take care of a number of small settings one would have to enable manually otherwise. These include an 'Imports:' and 'LinkingTo:' declaration in file DESCRIPTION, as well as 'useDynLib' and 'importFrom' in NAMESPACE. For *Rcpp Attributes* use, the `compileAttributes()` function has to be called. Similarly, to take advantage of its documentation-creation feature, the `roxygenize()` function from **roxygen2** has to be called.[7] Additional details on using *Rcpp* within a package scope are detailed in Eddelbuettel and François (2019c).

Conclusion

R has always provided mechanisms to extend it. The bare-bones *C* API is already used to great effect by a large number of packages. By taking advantage of a number of *C++* features, *Rcpp* has been able to make extending *R* easier, offering a combination of both speed *and* ease of use that has been finding increasingly widespread utilization by researchers and data scientists. We are thrilled about this adoption, and look forward to seeing more exciting extensions to *R* being built.

Acknowledgments. We thank Bob Rudis and Lionel Henry for excellent comments and suggestion on an earlier draft of this manuscript. Furthermore, we appreciate the improved *C++* annotated function graphic provided by Bob Rudis. This version is a pre-print of Eddelbuettel and Balamuta (2017, 2018).

References

Allaire JJ, Eddelbuettel D, François R (2019). *Rcpp Attributes*. Vignette included in R package Rcpp, URL http://CRAN.R-Project.org/package=Rcpp.

Anderson E, Bai Z, Bischof C, Blackford S, Demmel J, Dongarra J, Du Croz J, Greenbaum A, Hammarling S, McKenney A, Sorensen D (1999). *LAPACK Users' Guide*. Third edition. Society for Industrial and Applied Mathematics, Philadelphia, PA. ISBN 0-89871-447-8 (paperback).

Burger M, Juenemann K, Koenig T (2015). *RUnit: R Unit Test Framework*. R package version 0.4.31, URL https://CRAN.R-project.org/package=RUnit.

Chambers JM (2008). *Software for Data Analysis: Programming with R*. Statistics and Computing. Springer-Verlag, Heidelberg. ISBN 978-0-387-75935-7.

Chambers JM (2016). *Extending R*. The R Series. Chapman and Hall/CRC, London. ISBN 9781498775717.

Chang W, Luraschi J (2017). *profvis: Interactive Visualizations for Profiling R Code*. R package version 0.3.3, URL https://CRAN.R-project.org/package=profvis.

Dongarra JJ, Moler CB, Bunch JR, Stewart GW (1979). *LINPACK users' guide*. SIAM.

Eddelbuettel D (2013). *Seamless R and C++ Integration with Rcpp*. Use R! Springer, New York. ISBN 978-1-4614-6867-7.

Eddelbuettel D, Balamuta JJ (2017). "Extending R with C++: A Brief Introduction to Rcpp." *PeerJ Preprints*, **5**. doi:10.7287/peerj.preprints.3188v1/.

Eddelbuettel D, Balamuta JJ (2018). "Extending R with C++: A Brief Introduction to Rcpp." *The American Statistician*, **72**(1). doi:10.1080/00031305.2017.1375990.

Eddelbuettel D, François R (2011). "Rcpp: Seamless R and C++ Integration." *Journal of Statistical Software*, **40**(8), 1–18. URL http://www.jstatsoft.org/v40/i08/.

Eddelbuettel D, François R (2019a). *Frequently Asked Questions About Rcpp*. Vignette included in R package Rcpp, URL http://CRAN.R-Project.org/package=Rcpp.

Eddelbuettel D, François R (2019b). *Rcpp syntactic sugar*. Vignette included in R package Rcpp, URL http://CRAN.R-Project.org/package=Rcpp.

Eddelbuettel D, François R (2019c). *Writing a package that uses Rcpp*. Vignette included in R package Rcpp, URL http://CRAN.R-Project.org/package=Rcpp.

Eddelbuettel D, François R, Allaire J, Ushey K, Kou Q, Russel N, Chambers J, Bates D (2019a). *Rcpp: Seamless R and C++ Integration*. R package version 1.0.3, URL http://CRAN.R-Project.org/package=Rcpp.

Eddelbuettel D, François R, Bates D, Ni B (2019b). *RcppArmadillo: Rcpp integration for Armadillo templated linear algebra library*. R package version 0.9.800.1.0, URL http://CRAN.R-Project.org/package=RcppArmadillo.

Eddelbuettel D, Horner J (2017). *littler: R at the Command-Line via r*. R package version 0.3.2, URL http://CRAN.R-Project.org/package=littler.

Eddelbuettel D, Sanderson C (2014). "RcppArmadillo: Accelerating R with High-Performance C++ Linear Algebra." *Computational Statistics and Data Analysis*, **71**, 1054–1063. doi:10.1016/j.csda.2013.02.005. URL http://dx.doi.org/10.1016/j.csda.2013.02.005.

Efron B (1979). "Bootstrap Methods: Another Look at the Jackknife." *The Annals of Statistics*, **7**(1), 1–26. URL http://www.jstor.org/stable/2958830.

Guennebaud G, Jacob B, *et al.* (2012). "Eigen v3." URL http://eigen.tuxfamily.org.

Kusnierczyk W (2012). ***rbenchmark**: Benchmarking routine for R*. R package version 1.0.0, URL http://CRAN.R-Project.org/package=rbenchmark.

Mersmann O (2015). *microbenchmark: Accurate Timing Functions*. R package version 1.4-2.1, URL https://CRAN.R-project.org/package=microbenchmark.

R Core Team (2018a). *R: A Language and Environment for Statistical Computing*. R Foundation for Statistical Computing, Vienna, Austria. URL https://www.R-project.org/.

R Core Team (2018b). *Writing R extensions*. R Foundation for Statistical Computing, Vienna, Austria. URL http://CRAN.R-Project.org/doc/manuals/R-exts.html.

Sanderson C (2010). "Armadillo: An open source C++ Algebra Library for Fast Prototyping and Computationally Intensive Experiments." *Technical report*, NICTA. URL http://arma.sf.net.

Sanderson C, Curtin R (2016). "Armadillo: A Template-Based C++ Library for Linear Algebra." *JOSS*, **1**(2). doi:10.21105/joss.00026. URL http://dx.doi.org/10.21105/joss.00026.

Tandon A, Hocking TD (2015). *Rperform: Rperform - Performance testing for R packages*. R package version 0.0.0.9000.

Venables WN, Ripley BD (2002). *Modern Applied Statistics with S*. Fourth edition. Springer, New York. ISBN 0-387-95457-0, URL http://www.stats.ox.ac.uk/pub/MASS4.

Wickham H (2011). "testthat: Get Started with Testing." *The R Journal*, **3**, 5–10.

[7] The **littler** package (Eddelbuettel and Horner, 2017) has a helper script 'roxy.r' for this.
