# Supplementary material for: A biomathematical model of human erythropoiesis and iron metabolism
Source: Sci Rep. 2020 May 25;10:8602. doi: 10.1038/s41598-020-65313-5 (PMC7248076; doi:10.1038/s41598-020-65313-5)
Supplement: Supplementary file 5 — A biomathematical model of human erythropoiesis and iron metabolism: Simulation Model. [file 41598_2020_65313_MOESM5_ESM.zip › ErythroShort/Rcpp/doc/Rcpp-modules.pdf]

Exposing C++ functions and classes with Rcpp modules

Dirk Eddelbuettel[a] **and Romain François**[b]

[a]http://dirk.eddelbuettel.com; [b]https://romain.rbind.io/

This version was compiled on November 8, 2019

This note discusses *Rcpp modules*. *Rcpp modules* allow programmers to expose C++ functions and classes to R with relative ease. *Rcpp modules* are inspired from the Boost.Python C++ library (Abrahams and Grosse-Kunstleve, 2003) which provides similar features for Python.

Rcpp | modules | R | C++

1. Motivation

Exposing C++ functionality to R is greatly facilitated by the **Rcpp** package and its underlying C++ library (Eddelbuettel *et al.*, 2019; Eddelbuettel and François, 2011). **Rcpp** smoothes many of the rough edges in R and C++ integration by replacing the traditional R Application Programming Interface (API) described in '*Writing R Extensions*' (R Core Team, 2018) with a consistent set of C++ classes. The '*Rcpp-jss-2011*' vignette (Eddelbuettel *et al.*, 2019; Eddelbuettel and François, 2011) describes the API and provides an introduction to using **Rcpp**.

These **Rcpp** facilities offer a lot of assistance to the programmer wishing to interface R and C++. At the same time, these facilities are limited as they operate on a function-by-function basis. The programmer has to implement a `.Call` compatible function (to conform to the R API) using classes of the **Rcpp** API as described in the next section.

1.1. Exposing functions using Rcpp.

Exposing existing C++ functions to R through **Rcpp** usually involves several steps. One approach is to write an additional wrapper function that is responsible for converting input objects to the appropriate types, calling the actual worker function and converting the results back to a suitable type that can be returned to R (`SEXP`). Consider the `norm` function below:

```
double norm( double x, double y ) {
    return sqrt( x*x + y*y );
}
```

This simple function does not meet the requirements set by the `.Call` convention, so it cannot be called directly by R. Exposing the function involves writing a simple wrapper function that does match the `.Call` requirements. **Rcpp** makes this easy.

```
using namespace Rcpp;
RcppExport SEXP norm_wrapper(SEXP x_, SEXP y_) {
    // step 0: convert input to C++ types
    double x = as<double>(x_), y = as<double>(y_);

    // step 1: call the underlying C++ function
    double res = norm(x, y);

    // step 2: return the result as a SEXP
    return wrap(res);
}
```

Here we use the (templated) **Rcpp** converter `as()` which can transform from a `SEXP` to a number of different C++ and **Rcpp** types. The **Rcpp** function `wrap()` offers the opposite functionality and converts many known types to a `SEXP`.

This process is simple enough, and is used by a number of CRAN packages. However, it requires direct involvement from the programmer, which quickly becomes tiresome when many functions are involved. *Rcpp modules* provides a much more elegant and unintrusive way to expose C++ functions such as the `norm` function shown above to R.

We should note that **Rcpp** now has *Rcpp attributes* which extends certain aspect of *Rcpp modules* and makes binding to simple functions such as this one even easier. With *Rcpp attributes* we can just write

```
#include <Rcpp.h>

// [[Rcpp::export]]
double norm(double x, double y) {
    return sqrt(x*x + y*y);
}
```

See the corresponding vignette (Allaire *et al.*, 2019) for details, but read on for *Rcpp modules* which provide features not covered by *Rcpp attributes*, particularly when it comes to binding entire C++ classes and more.

1.2. Exposing classes using Rcpp.

Exposing C++ classes or structs is even more of a challenge because it requires writing glue code for each member function that is to be exposed.

Consider the simple `Uniform` class below:

```
class Uniform {
public:
    Uniform(double min_, double max_) :
       min(min_), max(max_) {}

    NumericVector draw(int n) {
        RNGScope scope;
        return runif(n, min, max);
    }

private:
    double min, max;
};
```

To use this class from R, we at least need to expose the constructor and the `draw` method. External pointers (R Core Team, 2018) are the perfect vessel for this, and using the `Rcpp:::XPtr` template from **Rcpp** we can expose the class with these two functions:

```
using namespace Rcpp;

/// create external pointer to a Uniform object
RcppExport SEXP Uniform__new(SEXP min_,
                             SEXP max_) {
    // convert inputs to appropriate C++ types
    double min = as<double>(min_),
           max = as<double>(max_);

    // create pointer to an Uniform object and
    // wrap it as an external pointer
    Rcpp::XPtr<Uniform>
    ptr( new Uniform( min, max ), true );

    // return the external pointer to the R side
    return ptr;
}

/// invoke the draw method
RcppExport SEXP Uniform__draw(SEXP xp, SEXP n_) {
    // grab the object as a XPtr (smart pointer)
    // to Uniform
    Rcpp::XPtr<Uniform> ptr(xp);

    // convert the parameter to int
    int n = as<int>(n_);

    // invoke the function
    NumericVector res = ptr->draw( n );

    // return the result to R
    return res;
}
```

As it is generally a bad idea to expose external pointers 'as is', they usually get wrapped as a slot of an S4 class.

Using `cxxfunction()` from the **inline** package, we can build this example on the fly. Suppose the previous example code assigned to a text variable `unifModCode`, we could then do

```
f1 <- cxxfunction( , "", includes = unifModCode,
                  plugin = "Rcpp" )
getDynLib(f1)  ## will display info about 'f1'
```

The following listing shows some *manual* wrapping to access the code, we will see later how this can be automated:

```
setClass("Uniform",
         representation( pointer = "externalptr"))

# helper
Uniform_method <- function(name) {
    paste("Uniform", name, sep = "__")
}

# syntactic sugar to allow object$method( ... )
setMethod("$", "Uniform", function(x, name) {
    function(...)
        .Call(Uniform_method(name) ,
              x@pointer, ...)
} )
```

```
# syntactic sugar to allow new( "Uniform", ... )
setMethod("initialize", "Uniform",
          function(.Object, ...) {
    .Object@pointer <-
        .Call(Uniform_method("new"), ...)
    .Object
} )

u <- new("Uniform", 0, 10)
u$draw( 10L )
```

Rcpp considerably simplifies the code that would be involved for using external pointers with the traditional R API. Yet this still involves a lot of mechanical code that quickly becomes hard to maintain and error prone. *Rcpp modules* offer an elegant way to expose the `Uniform` class in a way that makes both the internal C++ code and the R code easier.

2. Rcpp modules

The design of Rcpp modules has been influenced by Python modules which are generated by the `Boost.Python` library (Abrahams and Grosse-Kunstleve, 2003). Rcpp modules provide a convenient and easy-to-use way to expose C++ functions and classes to R, grouped together in a single entity.

A Rcpp module is created in C++ source code using the `RCPP_MODULE` macro, which then provides declarative code of what the module exposes to R.

This section provides an extensive description of how Rcpp modules are defined in standalone C++ code and loaded into R. Note however that defining and using Rcpp modules as part of other R packages simplifies the way modules are actually loaded, as detailed in Section 3 below.

2.1. Exposing C++ functions using Rcpp modules. Consider the `norm` function from the previous section. We can expose it to R:

```
using namespace Rcpp;

double norm(double x, double y) {
    return sqrt(x*x + y*y);
}

RCPP_MODULE(mod) {
    function("norm", &norm);
}
```

The code creates an Rcpp module called `mod` that exposes the `norm` function. **Rcpp** automatically deduces the conversions that are needed for input and output. This alleviates the need for a wrapper function using either **Rcpp** or the R API.

On the R side, the module is retrieved by using the `Module` function from **Rcpp**

```
inc <- '
using namespace Rcpp;

double norm( double x, double y ) {
    return sqrt(x*x + y*y);
}

RCPP_MODULE(mod) {
    function("norm", &norm);
```

```
}
'

fx <- cxxfunction(signature(),
                  plugin="Rcpp", include=inc)
mod <- Module("mod", getDynLib(fx))
```

Note that this example assumed that the previous code segment defining the module was returned by the `cxxfunction()` (from the **inline** package) as callable R function `fx` from which we can extract the relevant pointer using `getDynLib()` (again from **inline**).

Throughout the rest of the examples in this document, we always assume that the C++ code defining a module is used to create an object `fx` via a similar call to `cxxfunction`. As an alternative, one can also use `sourceCpp` as described in Section 2.3.

A module can contain any number of calls to `function` to register many internal functions to R. For example, these 6 functions:

```
std::string hello() {
    return "hello";
}

int bar( int x) {
    return x*2;
}

double foo( int x, double y) {
    return x * y;
}

void bla( ) {
    Rprintf("hello\\n");
}

void bla1( int x) {
    Rprintf("hello (x = %d)\\n", x);
}

void bla2( int x, double y) {
    Rprintf("hello (x = %d, y = %5.2f)\\n", x, y);
}
```

can be exposed with the following minimal code:

```
RCPP_MODULE(yada) {
    using namespace Rcpp;

    function("hello" , &hello);
    function("bar"   , &bar  );
    function("foo"   , &foo  );
    function("bla"   , &bla  );
    function("bla1"  , &bla1 );
    function("bla2"  , &bla2 );
}
```

which can then be used from R:

```
yada <- Module("yada", getDynLib(fx))
yada$bar(2L)
yada$foo(2L, 10.0)
yada$hello()
yada$bla()
yada$bla1(2L)
yada$bla2(2L, 5.0)
```

The requirements for a function to be exposed to R via Rcpp modules are:

- The function takes between 0 and 65 parameters.
- The type of each input parameter must be manageable by the `Rcpp::as` template.
- The return type of the function must be either `void` or any type that can be managed by the `Rcpp::wrap` template.
- The function name itself has to be unique in the module. In other words, no two functions with the same name but different signatures are allowed. C++ allows overloading functions. This might be added in future versions of modules.

2.1.1. Documentation for exposed functions using Rcpp modules. In addition to the name of the function and the function pointer, it is possible to pass a short description of the function as the third parameter of `function`.

```
using namespace Rcpp;

double norm(double x, double y) {
    return sqrt(x*x + y*y);
}

RCPP_MODULE(mod) {
    function("norm", &norm,
             "Provides a simple vector norm");
}
```

The description is used when displaying the function to the R prompt:

```
mod <- Module("mod", getDynLib(fx))
show(mod$norm)
```

2.1.2. Formal arguments specification. `function` also gives the possibility to specify the formal arguments of the R function that encapsulates the C++ function, by passing a `Rcpp::List` after the function pointer.

```
using namespace Rcpp;

double norm(double x, double y) {
    return sqrt(x*x + y*y);
}

RCPP_MODULE(mod_formals) {
    function("norm",
             &norm,
             List::create(_["x"] = 0.0,
                          _["y"] = 0.0),
             "Provides a simple vector norm");
}
```

A simple usage example is provided below:

```
mod <- Module("mod_formals", getDynLib(fx))
norm <- mod$norm
norm()
norm(x = 2, y = 3)
```

To set formal arguments without default values, omit the rhs.

```
using namespace Rcpp;

double norm(double x, double y) {
    return sqrt(x*x + y*y);
}

RCPP_MODULE(mod_formals2) {
    function("norm", &norm,
             List::create(_["x"], _["y"] = 0.0),
             "Provides a simple vector norm");
}
```

This can be used as follows:

```
mod <- Module("mod_formals2", getDynLib(fx))
norm <- mod$norm
args(norm)
```

The ellipsis (...) can be used to denote that additional arguments are optional; it does not take a default value.

```
using namespace Rcpp;

double norm(double x, double y) {
    return sqrt(x*x + y*y);
}

RCPP_MODULE(mod_formals3) {
    function("norm", &norm,
             List::create(_["x"], _["..."]),
             "documentation for norm");
}
```

This works similarly from the R side where the ellipsis is also understood:

```
mod <- Module("mod_formals3", getDynLib(fx))
norm <- mod$norm
args(norm)
```

2.2. Exposing C++ classes using Rcpp modules. Rcpp modules also provide a mechanism for exposing C++ classes, based on the reference classes introduced in R 2.12.0.

2.2.1. Initial example. A class is exposed using the `class_` keyword. The `Uniform` class may be exposed to R as follows:

```
using namespace Rcpp;
class Uniform {
public:
    Uniform(double min_, double max_) :
       min(min_), max(max_) {}

    NumericVector draw(int n) const {
        RNGScope scope;
        return runif(n, min, max);
    }

    double min, max;
};

double uniformRange(Uniform* w) {
    return w->max - w->min;
}

RCPP_MODULE(unif_module) {

    class_<Uniform>("Uniform")

    .constructor<double,double>()

    .field("min", &Uniform::min)
    .field("max", &Uniform::max)

    .method("draw", &Uniform::draw)
    .method("range", &uniformRange)
    ;

}
```

```
unif_module <- Module("unif_module",
                      getDynLib(fx))
Uniform <- unif_module$Uniform
u <- new(Uniform, 0, 10)
u$draw(10L)
u$range()
u$max <- 1
u$range()
u$draw(10)
```

`class_` is templated by the C++ class or struct that is to be exposed to R. The parameter of the `class_<Uniform>` constructor is the name we will use on the R side. It usually makes sense to use the same name as the class name. While this is not enforced, it might be useful when exposing a class generated from a template.

Then constructors, fields and methods are exposed.

2.2.2. Exposing constructors using Rcpp modules. Public constructors that take from 0 and 6 parameters can be exposed to the R level using the `.constructor` template method of `class_`.

Optionally, `.constructor` can take a description as the first argument.

```
 .constructor<double,double>("sets the min and "
     "max value of the distribution")
```

Also, the second argument can be a function pointer (called validator) matching the following type:

```
typedef bool (*ValidConstructor)(SEXP*,int);
```

The validator can be used to implement dispatch to the appropriate constructor, when multiple constructors taking the same number of arguments are exposed. The default validator always accepts the constructor as valid if it is passed the appropriate number of arguments. For example, with the call above, the default validator accepts any call from R with two `double` arguments (or arguments that can be cast to `double`).

TODO: include validator example here

2.2.3. Exposing fields and properties. `class_` has three ways to expose fields and properties, as illustrated in the example below:

```
using namespace Rcpp;
class Foo {
public:
    Foo(double x_, double y_, double z_):
        x(x_), y(y_), z(z_) {}

    double x;
    double y;

    double get_z() { return z; }
    void set_z(double z_) { z = z_; }

private:
    double z;
};

RCPP_MODULE(mod_foo) {
    class_<Foo>( "Foo" )

    .constructor<double,double,double>()

    .field("x", &Foo::x)
    .field_readonly("y", &Foo::y)

    .property("z", &Foo::get_z, &Foo::set_z)
    ;
}
```

The `.field` method exposes a public field with read/write access from R. It accepts an extra parameter to give a short description of the field:

```
  .field("x", &Foo::x, "documentation for x")
```

The `.field_readonly` exposes a public field with read-only access from R. It also accepts the description of the field.

```
  .field_readonly("y", &Foo::y,
                  "documentation for y")
```

The `.property` method allows indirect access to fields through a getter and a setter. The setter is optional, and the property is considered read-only if the setter is not supplied. A description of the property is also allowed:

```
  // with getter and setter
  .property("z", &Foo::get_z,
            &Foo::set_z, "Documentation for z")

  // with only getter
  .property("z",
            &Foo::get_z, "Documentation for z")
```

The type of the field (**T**) is deduced from the return type of the getter, and if a setter is given its unique parameter should be of the same type.

Getters can be member functions taking no parameter and returning a **T** (for example `get_z` above), or a free function taking a pointer to the exposed class and returning a **T**, for example:

```
double z_get(Foo* foo) { return foo->get_z(); }
```

Setters can be either a member function taking a **T** and returning `void`, such as `set_z` above, or a free function taking a pointer to the target class and a **T**:

```
void z_set(Foo* foo, double z) { foo->set_z(z); }
```

Using properties gives more flexibility in case field access has to be tracked or has impact on other fields. For example, this class keeps track of how many times the `x` field is read and written.

```
class Bar {
public:

    Bar(double x_) : x(x_), nread(0), nwrite(0) {}

    double get_x() {
        nread++;
        return x;
    }

    void set_x(double x_) {
        nwrite++;
        x = x_;
    }

    IntegerVector stats() const {
        return
        IntegerVector::create(_["read"] = nread,
                              _["write"] = nwrite);
    }

private:
    double x;
    int nread, nwrite;
};

RCPP_MODULE(mod_bar) {
    class_<Bar>( "Bar" )

    .constructor<double>()

    .property( "x", &Bar::get_x, &Bar::set_x )
    .method( "stats", &Bar::stats )
    ;
}
```

Here is a simple usage example:

```
mod_bar <- Module("mod_bar", getDynLib(fx))
Bar <- mod_bar$Bar
b <- new(Bar, 10)
b$x + b$x
b$stats()
b$x <- 10
b$stats()
```

2.2.4. Exposing methods using Rcpp modules. `class_` has several overloaded and templated `.method` functions allowing the programmer to expose a method associated with the class.

A legitimate method to be exposed by `.method` can be:

- A public member function of the class, either `const` or non-`const`, that returns `void` or any type that can be handled by

Rcpp::wrap, and that takes between 0 and 65 parameters whose types can be handled by Rcpp::as.
- A free function that takes a pointer to the target class as its first parameter, followed by 0 or more (up to 65) parameters that can be handled by Rcpp::as and returning a type that can be handled by Rcpp::wrap or void.

2.2.5. Documenting methods. .method can also include a short documentation of the method, after the method (or free function) pointer.

```
.method("stats", &Bar::stats,
        "vector indicating the number of "
        "times x has been read and written")
```

TODO: mention overloading, need good example.

2.2.6. Const and non-const member functions. .method is able to expose both const and non-const member functions of a class. There are however situations where a class defines two versions of the same method, differing only in their signature by the const-ness. It is for example the case of the member functions back of the std::vector template from the STL.

```
reference back ( );
const_reference back ( ) const;
```

To resolve the ambiguity, it is possible to use .const_method or .nonconst_method instead of .method in order to restrict the candidate methods.

2.2.7. Special methods. **Rcpp** considers the methods [[and [[<- special, and promotes them to indexing methods on the R side.

2.2.8. Object finalizers. The .finalizer member function of class_ can be used to register a finalizer. A finalizer is a free function that takes a pointer to the target class and return void. The finalizer is called before the destructor and so operates on a valid object of the target class.

It can be used to perform operations, releasing resources, etc …

The finalizer is called automatically when the R object that encapsulates the C++ object is garbage collected.

2.2.9. Object factories. The .factory member function of class_ can be used to register a factory that can be used as alternative to a constructor. A factory can be a static member function or a free function that returns a pointer to the target class. Typical use-cases include creating objects in a hierarchy:

```
#include <Rcpp.h>
using namespace Rcpp;

// abstract class
class Base {
    public:
        virtual ~Base() {}
        virtual std::string name() const = 0;
};

// first derived class
class Derived1: public Base {
    public:
        Derived1() : Base() {}
        virtual std::string name() const {
            return "Derived1";
        }
};

// second derived class
class Derived2: public Base {
    public:
        Derived2() : Base() {}
        virtual std::string name() const {
            return "Derived2";
        }
};

Base *newBase( const std::string &name ) {
    if (name == "d1"){
        return new Derived1;
    } else if (name == "d2"){
        return new Derived2;
    } else {
        return 0;
    }
}

RCPP_MODULE(mod) {
    Rcpp::class_< Base >("Base")
        .factory<const std::string&>(newBase)
        .method("name", &Base::name);
}
```

The newBase method returns a pointer to a Base object. Since that class is an abstract class, the objects are actually instances of Derived1 or Derived2. The same behavior is now available in R:

```
mod <- Module("mod", getDynLib(fx))
Base <- mod$Base
dv1 <- new(Base, "d1")
dv1$name() # returns "Derived1"
dv2 <- new(Base, "d2")
dv2$name() # returns "Derived2"
```

2.2.10. S4 dispatch. When a C++ class is exposed by the class_ template, a new S4 class is registered as well. The name of the S4 class is obfuscated in order to avoid name clashes (i.e. two modules exposing the same class). This allows implementation of R-level (S4) dispatch.

For example, consider the C++ class World exposed in module yada:

```
class World {
public:
    World() : msg("hello") {}
    void set(std::string msg) { this->msg = msg; }
    std::string greet() { return msg; }

private:
    std::string msg;
};

RCPP_MODULE(yada){
    using namespace Rcpp;
```

```
    class_<World>("World")

    // expose the default constructor
    .constructor()

    .method("greet", &World::greet)
    .method("set", &World::set)
    ;

}
```

The show method for World objects is then implemented as:

```
yada <- Module("yada", getDynLib(fx))
setMethod("show", yada$World , function(object) {
    msg <- paste("World object with message : ",
                 object$greet())
    writeLines(msg)
} )
yada$World$new() # implictly calls show
```

TODO: mention R inheritance (John ?)

2.2.11. Extending `Rcpp::as` and `Rcpp::wrap`. Sometimes it is necessary to extend `Rcpp::as` or `Rcpp::wrap` for classes that are also exposed using Rcpp modules. Instead of using the general methods described in the *Rcpp Extending* vignette, one can use the `RCPP_EXPOSED_AS` or `RCPP_EXPOSED_WRAP` macros. Alternatively the `RCPP_EXPOSED_CLASS` macro defines both `Rcpp::as` and `Rcpp::wrap` specializations. Do not use these macros together with the generic extension mechanisms. Note that opposed to the generic methods, these macros can be used *after* `Rcpp.h` has been loaded. Here an example of a pair of Rcpp modules exposed classes where one of them has a method taking an instance of the other class as argument. In this case it is sufficient to use `RCPP_EXPOSED_AS` to enable the transparent conversion from R to C++:

```
#include <Rcpp.h>

class Foo {
public:
    Foo() = default;
};

class Bar {
public:
    Bar() = default;
    void handleFoo(Foo foo) {
        Rcpp::Rcout << "Got a Foo!" << std::endl;
    };
};

RCPP_EXPOSED_AS(Foo)

RCPP_MODULE(Foo){
    Rcpp::class_<Foo>("Foo")
    .constructor();
}

RCPP_MODULE(Barl){
    Rcpp::class_<Bar>("Bar")
    .constructor()
    .method("handleFoo", &Bar::handleFoo);
}
```

```
Foo <- Module("Foo", getDynLib(fx))$Foo
Bar <- Module("Barl", getDynLib(fx))$Bar
foo <- new(Foo)
bar <- new(Bar)
bar$handleFoo(foo)
#> Got a Foo!
```

2.2.12. Full example. The following example illustrates how to use Rcpp modules to expose the class `std::vector<double>` from the STL.

```
typedef std::vector<double> vec;
void vec_assign(vec* obj,
                Rcpp::NumericVector data) {
    obj->assign(data.begin(), data.end());
}
void vec_insert(vec* obj, int position,
                Rcpp::NumericVector data) {
    vec::iterator it = obj->begin() + position;
    obj->insert(it, data.begin(), data.end());
}
Rcpp::NumericVector vec_asR( vec* obj ) {
   return Rcpp::wrap( *obj );
}
void vec_set(vec* obj, int i, double value) {
   obj->at( i ) = value;
}
// Fix for C++11, where we cannot directly expose
// member functions vec::resize and vec::push_back
void vec_resize (vec* obj, int n) {
    obj->resize(n);
}
void vec_push_back (vec* obj, double value) {
    obj->push_back(value);
}

RCPP_MODULE(mod_vec) {
    using namespace Rcpp;

    // we expose class std::vector<double>
    // as "vec" on the R side
    class_<vec>("vec")

    // exposing constructors
    .constructor()
    .constructor<int>()

    // exposing member functions
    .method("size", &vec::size)
    .method("max_size", &vec::max_size)
    .method("capacity", &vec::capacity)
    .method("empty", &vec::empty)
    .method("reserve", &vec::reserve)
    .method("pop_back", &vec::pop_back)
    .method("clear", &vec::clear)

    // exposing const member functions
```

```
    .const_method("back", &vec::back)
    .const_method("front", &vec::front)
    .const_method("at", &vec::at )

    // exposing free functions taking a
    // std::vector<double>* as their first
    // argument
    .method("assign", &vec_assign)
    .method("insert", &vec_insert)
    .method("resize", &vec_resize)
    .method("push_back", &vec_push_back)
    .method("as.vector", &vec_asR)

    // special methods for indexing
    .const_method("[[", &vec::at)
    .method("[[<-", &vec_set)
    ;
}
```

```
mod_vec <- Module("mod_vec", getDynLib(fx))
vec <- mod_vec$vec
v <- new(vec)
v$reserve(50L)
v$assign(1:10)
v$push_back(10)
v$size()
v$resize(30L)
v$capacity()
v[[ 0L ]]
v$as.vector()
```

2.3. Loading modules via sourceCpp. As an alternative to the explicit creation of a `Module` object using the **inline** package via `cxxfunction` and `getDynLib`, it is possible to use the `sourceCpp` function, accepting C++ source code as either a `.cpp` file or a character string and described in the *Rcpp attributes* vignette (Allaire *et al.*, 2019).

The main differences with this approach are:

- The `Rcpp.h` header file must be explicitly included.
- The content of the module (C++ functions and classes) is implicitly exposed and made available to R as individual objects, as opposed to being accessed from a `Module` object with the `$` extractor.

Note that this is similar to exposing modules in R packages using `loadModule`, described in Section 3.2.1 below.

As an example, consider a file called `yada.cpp` containing the following C++ code:

```
#include <Rcpp.h>
std::string hello() {
    return "hello";
}
void bla() {
    Rprintf("hello\\n");
}
void bla2( int x, double y) {
    Rprintf("hello (x = %d, y = %5.2f)\\n", x, y);
}

class World {
public:
    World() : msg("hello") {}
    void set(std::string msg) { this->msg = msg; }
    std::string greet() { return msg; }

private:
    std::string msg;
};

RCPP_MODULE(yada){
    using namespace Rcpp;

    function("hello" , &hello);
    function("bla"   , &bla);
    function("bla2"  , &bla2);

    class_<World>("World")
    .constructor()
    .method("greet", &World::greet)
    .method("set",   &World::set)
    ;
}
```

```
sourceCpp('yada.cpp')
```

C++ functions `hello`, `bla`, `bla2` and class `World` will be readily available in R:

```
hello()
bla()
bla2(42, 0.42)
w <- new(World)
w$greet()
w$set("hohoho")
w$greet()
```

3. Using modules in other packages

3.1. Namespace import. When using *Rcpp modules* in a packages, the client package needs to import **Rcpp**'s namespace. This is achieved by adding the following line to the `NAMESPACE` file.

```
import(Rcpp)
```

In some case we have found that explicitly naming a symbol can be preferable:

```
import(Rcpp, evalCpp)
```

3.2. Load the module in the namespace.

3.2.1. Load the module content via loadModule. Starting with release 0.9.11, the preferred way for loading a module directly into a package namespace is by calling the `loadModule()` function, which takes the module name as an argument and exposes the content of the module (C++ functions and classes) as individual objects in the namespace. It can be placed in any `.R` file in the package. This is useful as it allows to load the module from the same file as some auxiliary R functions using the module.

Consider a package **testmod** defining a module `yada` in the source file `src/yada.cpp`, with the same content as defined above in Section 2.3 above

Then, `loadModule` is called in the package's R code to expose all C++ functions and classes as objects `hello`, `bla`, `bla2`, `World` into the package namespace:

```
loadModule("yada", TRUE)
```

Provided the objects are also exported (see Section 3.3 below), this makes them readily available in R:

```
library(testmod)
hello()
bla()
bla2(42, 0.42)
w <- new(World)
w$greet()
w$set("hohoho")
w$greet()
```

The `loadModule` function has an argument `what` to control which objects are exposed in the package namespace. The special value `TRUE` means that all objects are exposed.

3.2.2. Deprecated legacy method using loadRcppModules.

Prior to release 0.9.11, where `loadModule` was introduced, loading all functions and classes from a module into a package namespace was achieved using the `loadRcppModules` function within the `.onLoad` body.

```
.onLoad <- function(libname, pkgname) {
    loadRcppModules()
}
```

This will look in the package's `DESCRIPTION` file for the `RcppModules` field, load each declared module and populate their contents into the package's namespace. For example, a package defining modules `yada`, `stdVector`, `NumEx` would have this declaration:

```
RcppModules: yada, stdVector, NumEx
```

The `loadRcppModules` function has a single argument `direct` with a default value of `TRUE`. With this default value, all content from the module is exposed directly in the package namespace. If set to `FALSE`, all content is exposed as components of the module.

Note: This approach is **deprecated** as of Rcpp 0.12.5, and now triggers a warning message. Eventually this function will be withdrawn.

3.2.3. Just expose the module.

Alternatively to exposing a module's content via `loadModule`, it is possible to just expose the module object to the users of the package, and let them extract the functions and classes as needed. This uses lazy loading so that the module is only loaded the first time the user attempts to extract a function or a class with the dollar extractor.

```
yada <- Module( "yada" )

.onLoad <- function(libname, pkgname) {
    # placeholder
}
```

Provided `yada` is properly exported, the functions and classes are accessed as e.g. `yada$hello`, `yada$World`.

3.3. Namespace exports.

The content of modules or the modules as a whole, exposed as objects in the package namespace, must be exported to be visible to users of the package. As for any other object, this is achieved by the appropriate `export()` or `exportPattern()` statements in the `NAMESPACE` file. For instance, the functions and classes in the `yada` module considered above can be exported as:

```
export(hello, bla, bla2, World)
```

3.4. Support for modules in skeleton generator.

Creating a new package using *Rcpp modules* is easiest via the call to `Rcpp.package.skeleton()` with argument `module=TRUE`.

```
Rcpp.package.skeleton("testmod", module = TRUE)
```

This will install code providing three example modules, exposed using `LoadModule`.

3.5. Module documentation.

Rcpp defines a `prompt` method for the `Module` class, allowing generation of a skeleton of an Rd file containing some information about the module.

```
yada <- Module("yada")
prompt(yada, "yada-module.Rd")
```

We strongly recommend using a package when working with Modules. But in case a manually compiled shared library has to loaded, the return argument of the `getDynLib()` function can be supplied as the `PACKAGE` argument to the `Module()` function as well.

4. Future extensions

`Boost.Python` has many more features that we would like to port to *Rcpp modules*: class inheritance, default arguments, enum types, ...

5. Known shortcomings

There are some things *Rcpp modules* is not good at:

- serialization and deserialization of objects: modules are implemented via an external pointer using a memory location, which is non-constant and varies between session. Objects have to be re-created, which is different from the (de-)serialization that R offers. So these objects cannot be saved from session to session.
- multiple inheritance: currently, only simple class structures are representable via *Rcpp modules*.

6. Summary

This note introduced *Rcpp modules* and illustrated how to expose C++ function and classes more easily to R. We hope that R and C++ programmers find *Rcpp modules* useful.

References

Abrahams D, Grosse-Kunstleve RW (2003). *Building Hybrid Systems with Boost.Python*. Boost Consulting. URL http://www.boostpro.com/writing/bpl.pdf.

Allaire JJ, Eddelbuettel D, François R (2019). *Rcpp Attributes*. Vignette included in R package Rcpp, URL http://CRAN.R-Project.org/package=Rcpp.

Eddelbuettel D, François R (2011). "Rcpp: Seamless R and C++ Integration." *Journal of Statistical Software*, **40**(8), 1–18. URL http://www.jstatsoft.org/v40/i08/.

Eddelbuettel D, François R, Allaire J, Ushey K, Kou Q, Russel N, Chambers J, Bates D (2019). *Rcpp: Seamless R and C++ Integration*. R package version 1.0.3, URL http://CRAN.R-Project.org/package=Rcpp.

R Core Team (2018). *Writing R extensions*. R Foundation for Statistical Computing, Vienna, Austria. URL http://CRAN.R-Project.org/doc/manuals/R-exts.html.
