# Supplementary material for: A biomathematical model of human erythropoiesis and iron metabolism
Source: Sci Rep. 2020 May 25;10:8602. doi: 10.1038/s41598-020-65313-5 (PMC7248076; doi:10.1038/s41598-020-65313-5)
Supplement: Supplementary file 5 — A biomathematical model of human erythropoiesis and iron metabolism: Simulation Model. [file 41598_2020_65313_MOESM5_ESM.zip › ErythroShort/Rcpp/doc/Rcpp-quickref.pdf]

Rcpp Quick Reference Guide

Dirk Eddelbuettel[a] **and Romain François**[b]

[a]http://dirk.eddelbuettel.com; [b]https://romain.rbind.io/

This version was compiled on November 8, 2019

This document provides short code snippets that are helpful for using the Rcpp (Eddelbuettel *et al.*, 2019; Eddelbuettel and François, 2011; Eddelbuettel, 2013).

Rcpp | quickref | R | C++

Important Notes

```
// If you experience compiler errors, please check
// that you have an appropriate version of g++.
// See `Rcpp-FAQ' for more information.

// Many of the examples here imply the following:
#include <Rcpp.h>
using namespace Rcpp;
// The cppFunction will automatically add this.

// Or, prefix Rcpp objects with the Rcpp namespace
// as e.g. in:
Rcpp::NumericVector xx(10);
```

Create simple vectors

```
SEXP x; std::vector<double> y(10);

// from SEXP
NumericVector xx(x);

// of a given size (filled with 0)
NumericVector xx(10);
// ... with a default for all values
NumericVector xx(10, 2.0);

// range constructor
NumericVector xx(y.begin(), y.end());

// using create
NumericVector xx =
    NumericVector::create(1.0, 2.0, 3.0, 4.0);
NumericVector yy =
    NumericVector::create(Named("foo") = 1.0,
                          _["bar"]     = 2.0);
                          // _ short for Named
```

Extract and set single elements

```
// extract single values
double x0 = xx[0];
double x1 = xx(1);

double y0 = yy["foo"];
double y1 = yy["bar"];

// set single values
xx[0] = 2.1;
xx(1) = 4.2;

yy["foo"] = 3.0;

// grow the vector
yy["foobar"] = 10.0;
```

Using matrices

```
// Initializing from SEXP,
// dimensions handled automatically
SEXP x;
NumericMatrix xx(x);

// Matrix of 4 rows & 5 columns (filled with 0)
NumericMatrix xx(4, 5);

// Fill with value
int xsize = xx.nrow() * xx.ncol();
for (int i = 0; i < xsize; i++) {
    xx[i] = 7;
}
// Same as above, using STL fill
std::fill(xx.begin(), xx.end(), 8);

// Assign this value to single element
// (1st row, 2nd col)
xx(0,1) = 4;

// Reference the second column
// Changes propagate to xx (same applies for Row)
NumericMatrix::Column zzcol = xx( _, 1);
zzcol = zzcol * 2;

// Copy the second column into new object
NumericVector zz1 = xx( _, 1);
// Copy submatrix (top left 3x3) into new object
NumericMatrix zz2 = xx( Range(0,2), Range(0,2));
```

Inline C++ Compile in R

```
## Note - this is R code.
## cppFunction in Rcpp allows rapid testing.
require(Rcpp)

cppFunction("
NumericVector exfun(NumericVector x, int i){
    x = x*i;
    return x;
}")
```

```r
exfun(1:5, 3)

## Use evalCpp to evaluate C++ expressions
evalCpp("std::numeric_limits<double>::max()")
```

Interface with R

First step in R.

```r
# In R, create a package shell. For details,
# see the "Writing R Extensions" manual and
# the "Rcpp-package" vignette.

Rcpp.package.skeleton("myPackage")

# Add R code to pkg R/ directory. Call C++
# function. Do type-checking in R.

myfunR <- function(Rx, Ry) {
    ret = .Call("myCfun", Rx, Ry,
                package="myPackage")
    return(ret)
}
```

Additional C++.

```cpp
// Add C++ code to pkg src/ directory.
using namespace Rcpp;
// Define function as extern with RcppExport
RcppExport SEXP myCfun( SEXP x, SEXP y) {
    // If R/C++ types match, use pointer to x.
    // Pointer is faster, but changes to xx
    // propagate to R ( xx -> x == Rx).
    NumericVector xx(x);

    // clone is slower and uses extra memory.
    // Safe. No side effects.
    NumericVector yy(clone(y));

    xx[0] = yy[0] = -1.5;
    int zz = xx[0];

    // use wrap() to return non-SEXP objects, e.g:
    // return(wrap(zz));
    // Build and return a list
    List ret;
    ret["x"] = xx;
    ret["y"] = yy;
    return(ret);
}
```

On the command-line.

```
# From shell, above package directory
R CMD build myPackage
R CMD check myPackage_1.0.tar.gz  ## Optional
R CMD INSTALL myPackage_1.0.tar.gz
```

Back in R.

```r
require(myPackage)

aa <- 1.5
bb <- 1.5
cc <- myfunR(aa, bb)
aa == bb
# FALSE, C++ modifies aa

aa <- 1:2
bb <- 1:2
cc <-  myfunR(aa, bb)
identical(aa, bb)
# TRUE, R/C++ types don't match
# so a copy was made
```

STL interface

```cpp
// sum a vector from beginning to end
double s = std::accumulate(x.begin(),
                           x.end(), 0.0);
// prod of elements from beginning to end
int p = std::accumulate(vec.begin(),
                        vec.end(), 1,
                        std::multiplies<int>());
// inner_product to compute sum of squares
double s2 = std::inner_product(res.begin(),
                               res.end(),
                               res.begin(), 0.0);
```

Rcpp Attributes

In C++.

```cpp
// Add code below into C++ file Rcpp_example.cpp

#include <Rcpp.h>
using namespace Rcpp;

// Place the 'Rcpp::export' tag
// right above function declaration.

// [[Rcpp::export]]
double muRcpp(NumericVector x){
    int n = x.size(); // Size of vector
    double sum = 0;   // Sum value

    // For loop, note cpp index shift to 0
    for(int i = 0; i < n; i++){
        // Shorthand for sum = sum + x[i]
        sum += x[i];
    }

    return sum/n;  // Obtain and return the Mean
}

// Place dependent functions above call or
// declare the function definition with:
double muRcpp(NumericVector x);

// [[Rcpp::export]]
```

```
double varRcpp(NumericVector x, bool bias = true){
    // Calculate the mean using C++ function
    double mean = muRcpp(x);
    double sum = 0;
    int n = x.size();

    for(int i = 0; i < n; i++){
        sum += pow(x[i] - mean, 2.0); // Square
    }

    return sum/(n-bias); // Return variance
}
```

In R:.

```
Rcpp::sourceCpp("path/to/file/Rcpp_example.cpp")
x <- 1:5
all.equal(muRcpp(x), mean(x))
all.equal(var(x),varRcpp(x))
```

Rcpp Extensions

```
// Enable C++11
// [[Rcpp::plugins(cpp11)]]

// Enable OpenMP (excludes macOS)
// [[Rcpp::plugins(openmp)]]

// Use the RcppArmadillo package
// Requires different header file from Rcpp.h
#include <RcppArmadillo.h>
// [[Rcpp::depends(RcppArmadillo)]]
```

Rcpp sugar

```
NumericVector x =
    NumericVector::create(-2.0,-1.0,0.0,1.0,2.0);
IntegerVector y =
    IntegerVector::create(-2, -1, 0, 1, 2);

NumericVector xx = abs( x );
IntegerVector yy = abs( y );

bool b = all( x < 3.0 ).is_true() ;
bool b = any( y > 2 ).is_true();

NumericVector xx = ceil( x );
NumericVector xx = ceiling( x );
NumericVector yy = floor( y );
NumericVector yy = floor( y );

NumericVector xx = exp( x );
NumericVector yy = exp( y );

NumericVector xx = head( x, 2 );
IntegerVector yy = head( y, 2 );

IntegerVector xx = seq_len( 10 );
IntegerVector yy = seq_along( y );
```

```
NumericVector xx = rep( x, 3 );
NumericVector xx = rep_len( x, 10 );
NumericVector xx = rep_each( x, 3 );

IntegerVector yy = rev( y );
```

Random Number Generation functions}

```
// Set seed
RNGScope scope;

// For details see Section 6.7.1--Distribution
// functions of the `Writing R Extensions' manual.
// In some cases (e.g. rnorm), dist-specific
// arguments can be omitted; when in doubt,
// specify all dist-specific arguments. The use
// of doublesrather than integers for dist-
// specific arguments is recommended. Unless
// explicitly specified, log=FALSE.

// Equivalent to R calls
NumericVector xx = runif(20);
NumericVector xx1 = rnorm(20);
NumericVector xx1 = rnorm(20, 0);
NumericVector xx1 = rnorm(20, 0, 1);

// Example vector of quantiles
NumericVector quants(5);
for (int i = 0; i < 5; i++) {
    quants[i] = (i-2);
}

// in R, dnorm(-2:2)
NumericVector yy = dnorm(quants) ;
NumericVector yy = dnorm(quants, 0.0, 1.0) ;

// in R, dnorm(-2:2, mean=2, log=TRUE)
NumericVector yy = dnorm(quants, 2.0, true) ;

// Note - cannot specify sd without mean
// in R, dnorm(-2:2, mean=0, sd=2, log=TRUE)
NumericVector yy = dnorm(quants, 0.0, 2.0, true) ;

// To get original R api, use Rf_*
double zz = Rf_rnorm(0, 2);
```

Environment

```
// Special environments
Environment::Rcpp_namespace();
Environment::base_env();
Environment::base_namespace();
Environment::global_env();
Environment::empty_env();

// Obtain an R environment
Environment stats("package:stats");
Environment env( 2 ); // by position
Environment glob = Environment::global_env();
```

```
// Extract function from specific environment
Function rnorm = stats["rnorm"];

// Assign into the environment
glob["x"] = "foo";
glob["y"] = 3;

// Retrieve information from environment
std::string x = glob["x"];
glob.assign( "foo" , 3 );
int foo = glob.get( "foo" );
int foo = glob.find( "foo" );
CharacterVector names = glob.ls(TRUE)
bool b = glob.exists( "foo" );
glob.remove( "foo" );

// Administration
glob.lockBinding("foo");
glob.unlockBinding("foo");
bool b = glob.bindingIsLocked("foo");
bool b = glob.bindingIsActive("foo");

// Retrieve related environments
Environment e = stats.parent();
Environment e = glob.new_child();
```

Calling Functions in R

```
// Do NOT expect to have a performance gain
// when calling R functions from R!

// Retrieve functions from default loaded env.
Function rnorm("rnorm");
rnorm(100, _["mean"] = 10.2, _["sd"] = 3.2 );

// Passing in an R function and obtaining results
// Make sure function conforms with return type!
NumericVector callFunction(NumericVector x,
                           Function f) {
    NumericVector res = f(x);
    return res;
}

/*** R
# The following is R code executed
# by sourceCpp() as a convenience.
x = 1:5
callFunction(x, sum)
*/
```

Modules

```
// Warning -- Module-based objects do not persist
// across quit(save="yes")/reload cycles.  To be
// safe, save results to R objects and remove
// module objects before exiting R.

// To create a module-containing package from R:
// Rcpp.package.skeleton("mypackage", module=TRUE)


class Bar {
  public:
    Bar(double x_) : x(x_), nread(0), nwrite(0) {}

    double get_x( ) {
      nread++;
      return x;
    }

    void set_x( double x_) {
      nwrite++;
      x = x_;
    }

    IntegerVector stats() const {
      return
      IntegerVector::create(_["read"] = nread,
                            _["write"] = nwrite);
    }
  private:
    double x; int nread, nwrite;
};

RCPP_MODULE(mod_bar) {
  class_<Bar>( "Bar" )
  .constructor<double>()
  .property( "x", &Bar::get_x, &Bar::set_x,
    "Docstring for x" )
  .method( "stats", &Bar::stats,
    "Docstring for stats")
;}

/*** R
## The following is R code.
require(mypackage) s
how(Bar)
b <- new(Bar, 10)
b$x <- 10
b_persist <- list(stats=b$stats(), x=b$x)
rm(b)
*/
```

References

Eddelbuettel D (2013). *Seamless R and C++ Integration with Rcpp*. Use R! Springer, New York. ISBN 978-1-4614-6867-7.

Eddelbuettel D, François R (2011). “Rcpp: Seamless R and C++ Integration.” *Journal of Statistical Software*, **40**(8), 1–18. URL http://www.jstatsoft.org/v40/i08/.

Eddelbuettel D, François R, Allaire J, Ushey K, Kou Q, Russel N, Chambers J, Bates D (2019). *Rcpp: Seamless R and C++ Integration*. R package version 1.0.3, URL http://CRAN.R-Project.org/package=Rcpp.
