# Supplementary material for: A biomathematical model of human erythropoiesis and iron metabolism
Source: Sci Rep. 2020 May 25;10:8602. doi: 10.1038/s41598-020-65313-5 (PMC7248076; doi:10.1038/s41598-020-65313-5)
Supplement: Supplementary file 5 — A biomathematical model of human erythropoiesis and iron metabolism: Simulation Model. [file 41598_2020_65313_MOESM5_ESM.zip › ErythroShort/Rcpp/doc/Rcpp-sugar.pdf]

Rcpp syntactic sugar

Dirk Eddelbuettel[a] **and Romain François**[b]

[a]http://dirk.eddelbuettel.com; [b]https://romain.rbind.io/

This version was compiled on November 8, 2019

This note describes *Rcpp sugar* which has been introduced in version 0.8.3 of Rcpp (Eddelbuettel *et al.*, 2019a; Eddelbuettel and François, 2011). *Rcpp sugar* brings a higher-level of abstraction to C++ code written using the Rcpp API. *Rcpp sugar* is based on expression templates (Abrahams and Gurtovoy, 2004; Vandevoorde and Josuttis, 2003) and provides some 'syntactic sugar' facilities directly in Rcpp. This is similar to how RcppArmadillo (Eddelbuettel *et al.*, 2019b) offers linear algebra C++ classes based on Armadillo (Sanderson, 2010).

Rcpp | sugar | R | C++

1. Motivation

Rcpp facilitates development of internal compiled code in an R package by abstracting low-level details of the R API (R Core Team, 2018) into a consistent set of C++ classes.

Code written using **Rcpp** classes is easier to read, write and maintain, without loosing performance. Consider the following code example which provides a function `foo` as a C++ extension to R by using the **Rcpp** API:

```
RcppExport SEXP foo(SEXP x, SEXP y) {
    Rcpp::NumericVector xx(x), yy(y);
    int n = xx.size();
    Rcpp::NumericVector res(n);
    double x_ = 0.0, y_ = 0.0;
    for (int i=0; i<n; i++) {
        x_ = xx[i];
        y_ = yy[i];
        if (x_ < y_) {
            res[i] = x_ * x_;
        } else {
            res[i] = -(y_ * y_);
        }
    }
    return res;
}
```

The goal of the function `foo` code is simple. Given two `numeric` vectors, we create a third one. This is typical low-level C++ code that that could be written much more consicely in R thanks to vectorisation as shown in the next example.

```
foo <- function(x, y) {
    ifelse(x < y, x * x, -(y * y))
}
```

Put succinctly, the motivation of *Rcpp sugar* is to bring a subset of the high-level R syntax in C++. Hence, with *Rcpp sugar* , the C++ version of `foo` now becomes:

```
Rcpp::NumericVector foo(Rcpp::NumericVector x,
                        Rcpp::NumericVector y) {
    return ifelse(x < y, x * x, -(y * y));
}
```

Apart from being strongly-typed and the need for explicit `return` statement, the code is now identical between highly-vectorised R and C++.

Rcpp sugar is written using expression templates and lazy evaluation techniques (Abrahams and Gurtovoy, 2004; Vandevoorde and Josuttis, 2003). This not only allows a much nicer high-level syntax, but also makes it rather efficient (as we detail in section 4 below).

2. Operators

Rcpp sugar takes advantage of C++ operator overloading. The next few sections discuss several examples.

2.1. Binary arithmetic operators. *Rcpp sugar* defines the usual binary arithmetic operators : `+`, `-`, `*`, `/`.

```
// two numeric vectors of the same size
NumericVector x;
NumericVector y;

// expressions involving two vectors
NumericVector res = x + y;
NumericVector res = x - y;
NumericVector res = x * y;
NumericVector res = x / y;

// one vector, one single value
NumericVector res = x + 2.0;
NumericVector res = 2.0 - x;
NumericVector res = y * 2.0;
NumericVector res = 2.0 / y;

// two expressions
NumericVector res = x * y + y / 2.0;
NumericVector res = x * (y - 2.0);
NumericVector res = x / (y * y);
```

The left hand side (lhs) and the right hand side (rhs) of each binary arithmetic expression must be of the same type (for example they should be both `numeric` expressions).

The lhs and the rhs can either have the same size or one of them could be a primitive value of the appropriate type, for example adding a `NumericVector` and a `double`.

2.2. Binary logical operators. Binary logical operators create a `logical` sugar expression from either two sugar expressions of the same type or one sugar expression and a primitive value of the associated type.

```
// two integer vectors of the same size
NumericVector x;
NumericVector y;

// expressions involving two vectors
```

```
LogicalVector res = x < y;
LogicalVector res = x > y;
LogicalVector res = x <= y;
LogicalVector res = x >= y;
LogicalVector res = x == y;
LogicalVector res = x != y;

// one vector, one single value
LogicalVector res = x < 2;
LogicalVector res = 2 > x;
LogicalVector res = y <= 2;
LogicalVector res = 2 != y;

// two expressions
LogicalVector res = (x + y) <  (x*x);
LogicalVector res = (x + y) >= (x*x);
LogicalVector res = (x + y) == (x*x);
```

2.3. Unary operators. The unary `operator-` can be used to negate a (numeric) sugar expression. whereas the unary `operator!` negates a logical sugar expression:

```
// a numeric vector
NumericVector x;

// negate x
NumericVector res = -x;

// use it as part of a numerical expression
NumericVector res = -x * (x + 2.0);

// two integer vectors of the same size
NumericVector y;
NumericVector z;

// negate the logical expression "y < z"
LogicalVector res = !(y < z);
```

3. Functions

Rcpp sugar defines functions that closely match the behavior of R functions of the same name.

3.1. Functions producing a single logical result. Given a logical sugar expression, the `all` function identifies if all the elements are `TRUE`. Similarly, the `any` function identifies if any the element is `TRUE` when given a logical sugar expression.

```
IntegerVector x = seq_len(1000);
all(x*x < 3);
any(x*x < 3);
```

Either call to `all` and `any` creates an object of a class that has member functions `is_true`, `is_false`, `is_na` and a conversion to `SEXP` operator.

One important thing to highlight is that `all` is lazy. Unlike R, there is no need to fully evaluate the expression. In the example above, the result of `all` is fully resolved after evaluating only the two first indices of the expression `x * x < 3`. `any` is lazy too, so it will only need to resolve the first element of the example above.

3.1.1. Conversion to bool. One important thing to note concerns the conversion to the `bool` type. In order to respect the concept of missing values (`NA`) in R, expressions generated by `any` or `all` can not be converted to `bool`. Instead one must use `is_true`, `is_false` or `is_na`:

```
// wrong: will generate a compile error
bool res = any(x < y);

// ok
bool res = is_true(any( x < y ));
bool res = is_false(any( x < y ));
bool res = is_na(any( x < y ));
```

3.2. Functions producing sugar expressions.

3.2.1. is_na. Given a sugar expression of any type, `is_na` (just like the other functions in this section) produces a logical sugar expression of the same length. Each element of the result expression evaluates to `TRUE` if the corresponding input is a missing value, or `FALSE` otherwise.

```
IntegerVector x =
    IntegerVector::create(0, 1, NA_INTEGER, 3);

is_na(x)
all(is_na( x ))
any(!is_na( x ))
```

3.2.2. seq_along. Given a sugar expression of any type, `seq_along` creates an integer sugar expression whose values go from 1 to the size of the input.

```
IntegerVector x =
    IntegerVector::create( 0, 1, NA_INTEGER, 3 );

IntegerVector y = seq_along(x);
IntegerVector z = seq_along(x * x * x * x * x * x);
```

This is the most lazy function, as it only needs to call the `size` member function of the input expression. The input expression need not to be resolved. The two examples above gives the same result with the same efficiency at runtime. The compile time will be affected by the complexity of the second expression, since the abstract syntax tree is built at compile time.

3.2.3. seq_len. `seq_len` creates an integer sugar expression whose i-th element expands to `i`. `seq_len` is particularly useful in conjunction with `sapply` and `lapply`.

```
// 1, 2, ..., 10
IntegerVector x = seq_len(10);

List y = lapply(seq_len(10), seq_len);
```

3.2.4. pmin and pmax. Given two sugar expressions of the same type and size, or one expression and one primitive value of the appropriate type, `pmin` (`pmax`) generates a sugar expression of the same type whose i-th element expands to the lowest (highest) value between the i-th element of the first expression and the i-thelement of the second expression.

```
IntegerVector x = seq_len(10);

pmin(x, x*x);
pmin(x*x, 2);

pmin(x, x*x);
pmin(x*x, 2);
```

3.2.5. ifelse. Given a logical sugar expression and either :

- two compatible sugar expression (same type, same size)
- one sugar expression and one compatible primitive

`ifelse` expands to a sugar expression whose i-th element is the i-th element of the first expression if the i-th element of the condition expands to `TRUE` or the i-th of the second expression if the i-th element of the condition expands to `FALSE`, or the appropriate missing value otherwise.

```
IntegerVector x;
IntegerVector y;

ifelse(x < y, x, (x+y)*y)
ifelse(x > y, x, 2)
```

3.2.6. sapply. `sapply` applies a C++ function to each element of the given expression to create a new expression. The type of the resulting expression is deduced by the compiler from the result type of the function.

The function can be a free C++ function such as the overload generated by the template function below:

```
template <typename T>
T square(const T& x){
    return x * x;
}
sapply(seq_len(10), square<int>);
```

Alternatively, the function can be a functor whose type has a nested type called `result_type`

```
template <typename T>
struct square : std::unary_function<T, T> {
    T operator()(const T& x){
        return x * x;
    }
}
sapply(seq_len(10), square<int>());
```

3.2.7. lapply. `lapply` is similar to `sapply` except that the result is allways an list expression (an expression of type `VECSXP`).

3.2.8. sign. Given a numeric or integer expression, `sign` expands to an expression whose values are one of 1, 0, -1 or `NA`, depending on the sign of the input expression.

```
IntegerVector xx;

sign(xx)
sign(xx * xx)
```

3.2.9. diff. The i-th element of the result of `diff` is the difference between the $(i+1)$th and the i-th element of the input expression. Supported types are integer and numeric.

```
IntegerVector xx;

diff(xx)
```

3.3. Mathematical functions. For the following set of functions, generally speaking, the i-th element of the result of the given function (say, `abs`) is the result of applying that function to this i-th element of the input expression. Supported types are integer and numeric.

```
IntegerVector x;

abs(x)
exp(x)
floor(x)
ceil(x)
pow(x, z)     // x to the power of z
```

3.4. The d/q/p/r statistical functions. The framework provided by *Rcpp sugar* also permits easy and efficient access the density, distribution function, quantile and random number generation functions function by R in the `Rmath` library.

Currently, most of these functions are vectorised for the first element which denote size. Consequently, these calls works in C++ just as they would in R:

```
x1 = dnorm(y1, 0, 1); // density of y1 at m=0, sd=1
x2 = qnorm(y2, 0, 1); // quantiles of y2
x3 = pnorm(y3, 0, 1); // distribution of y3
x4 = rnorm(n, 0, 1);  // 'n' RNG draws of N(0, 1)
```

Similar d/q/p/r functions are provided for the most common distributions: beta, binom, cauchy, chisq, exp, f, gamma, geom, hyper, lnorm, logis, nbeta, nbinom, nbinom_mu, nchisq, nf, norm, nt, pois, t, unif, and weibull.

Note that the parameterization used in these sugar functions may differ between the top-level functions exposed in an R session. For example, the internal `rexp` is parameterized by `scale`, whereas the R-level `stats::rexp` is parameterized by `rate`. Consult Distribution Functions for more details on the parameterization used for these sugar functions.

One point to note is that the programmer using these functions needs to initialize the state of the random number generator as detailed in Section 6.3 of the 'Writing R Extensions' manual (R Core Team, 2018). A nice C++ solution for this is to use a *scoped* class that sets the random number generatator on entry to a block and resets it on exit. We offer the `RNGScope` class which allows code such as

```
RcppExport SEXP getRGamma() {
    RNGScope scope;
    NumericVector x = rgamma(10, 1, 1);
    return x;
}
```

As there is some computational overhead involved in using `RNGScope`, we are not wrapping it around each inner function. Rather, the user of these functions (*i.e.* you) should place an `RNGScope` at the appropriate level of your code.

4. Performance

TBD

5. Implementation

This section details some of the techniques used in the implementation of *Rcpp sugar* . Note that the user need not to be familiar with the implementation details in order to use *Rcpp sugar* , so this section can be skipped upon a first read of the paper.

Writing *Rcpp sugar* functions is fairly repetitive and follows a well-structured pattern. So once the basic concepts are mastered (which may take time given the inherent complexities in template programming), it should be possible to extend the set of function further following the established pattern.

5.1. The curiously recurring template pattern. Expression templates such as those used by *Rcpp sugar* use a technique called the *Curiously Recurring Template Pattern* (CRTP). The general form of CRTP is:

```
// The Curiously Recurring Template Pattern (CRTP)
template <typename T>
struct base {
    // ...
};
struct derived : base<derived> {
    // ...
};
```

The `base` class is templated by the class that derives from it : `derived`. This shifts the relationship between a base class and a derived class as it allows the base class to access methods of the derived class.

5.2. The VectorBase class. The CRTP is used as the basis for *Rcpp sugar* with the `VectorBase` class template. All sugar expression derive from one class generated by the `VectorBase` template. The current definition of `VectorBase` is given here:

```
template <int RTYPE, bool na, typename VECTOR>
class VectorBase {
public:
    struct r_type :
        traits::integral_constant<int,RTYPE>{};
    struct can_have_na :
        traits::integral_constant<bool,na>{};

    typedef typename
        traits::storage_type<RTYPE>::type
        stored_type;

    VECTOR& get_ref(){
        return static_cast<VECTOR&>(*this);
    }

    inline stored_type operator[](int i) const {
        return static_cast<const VECTOR*>(
                           this)->operator[](i);
    }

    inline int size() const {
        return static_cast<const VECTOR*>(
                           this)->size();
    }

    /* definition ommited here */
    class iterator;

    inline iterator begin() const {
        return iterator(*this, 0);
    }
    inline iterator end() const {
        return iterator(*this, size());
    }
}
```

The `VectorBase` template has three parameters:

- `RTYPE`: This controls the type of expression (INTSXP, REALSXP, ...)
- `na`: This embeds in the derived type information about whether instances may contain missing values. **Rcpp** vector types (`IntegerVector`, ...) derive from `VectorBase` with this parameter set to `true` because there is no way to know at compile-time if the vector will contain missing values at run-time. However, this parameter is set to `false` for types that are generated by sugar expressions as these are guaranteed to produce expressions that are without missing values. An example is the `is_na` function. This parameter is used in several places as part of the compile time dispatch to limit the occurence of redundant operations.
- `VECTOR`: This parameter is the key of *Rcpp sugar* . This is the manifestation of CRTP. The indexing operator and the `size` method of `VectorBase` use a static cast of `this` to the `VECTOR` type to forward calls to the actual method of the derived class.

5.3. Example: sapply. As an example, the current implementation of `sapply`, supported by the template class `Rcpp::sugar::Sapply` is given below:

```
template <int RTYPE, bool NA,
          typename T, typename Function>
class Sapply : public VectorBase<
    Rcpp::traits::r_sexptype_traits< typename
        ::Rcpp::traits::result_of<Function>::type
    >::rtype,
    true,
    Sapply<RTYPE, NA, T, Function>
> {
public:
    typedef typename
      ::Rcpp::traits::result_of<Function>::type;

    const static int RESULT_R_TYPE =
        Rcpp::traits::r_sexptype_traits<
            result_type>::rtype;

    typedef Rcpp::VectorBase<RTYPE,NA,T> VEC;

    typedef typename
        Rcpp::traits::r_vector_element_converter<
        RESULT_R_TYPE>::type
        converter_type;
```

```
    typedef typename Rcpp::traits::storage_type<
        RESULT_R_TYPE>::type STORAGE;

    Sapply(const VEC& vec_, Function fun_) :
        vec(vec_), fun(fun_){}

    inline STORAGE operator[]( int i ) const {
        return converter_type::get(fun(vec[i]));
    }

    inline int size() const {
        return vec.size();
    }

private:
    const VEC& vec;
    Function fun;
};

// sugar

template <int RTYPE, bool _NA_,
          typename T, typename Function >
inline sugar::Sapply<RTYPE, _NA_, T, Function>
sapply(const Rcpp::VectorBase<RTYPE,_NA_,T>& t,
        Function fun) {

    return
      sugar::Sapply<RTYPE,_NA_,T,Function>(t, fun);
}
```

5.3.1. The sapply function. sapply is a template function that takes two arguments. The first argument is a sugar expression, which we recognize because of the relationship with the VectorBase class template. The second argument is the function to apply.

The sapply function itself does not do anything, it is just used to trigger compiler detection of the template parameters that will be used in the sugar::Sapply template.

5.3.2. Detection of return type of the function. In order to decide which kind of expression is built, the Sapply template class queries the template argument via the Rcpp::traits::result_of template.

```
typedef typename
    ::Rcpp::traits::result_of<Function>::type
    result_type;
```

The result_of type trait is implemented as such:

```
template <typename T>
struct result_of{
    typedef typename T::result_type type;
};

template <typename RESULT_TYPE,
          typename INPUT_TYPE>
struct result_of<RESULT_TYPE (*)(INPUT_TYPE)> {
    typedef RESULT_TYPE type;
};
```

The generic definition of result_of targets functors with a nested result_type type.

The second definition is a partial specialization targetting function pointers.

5.3.3. Indentification of expression type. Based on the result type of the function, the r_sexptype_traits trait is used to identify the expression type.

```
const static int RESULT_R_TYPE =
    Rcpp::traits::r_sexptype_traits<
        result_type>::rtype;
```

5.3.4. Converter. The r_vector_element_converter class is used to convert an object of the function's result type to the actual storage type suitable for the sugar expression.

```
typedef typename
    Rcpp::traits::r_vector_element_converter<
        RESULT_R_TYPE>::type
    converter_type;
```

5.3.5. Storage type. The storage_type trait is used to get access to the storage type associated with a sugar expression type. For example, the storage type of a REALSXP expression is double.

```
typedef typename
    Rcpp::traits::storage_type<RESULT_R_TYPE>::type
    STORAGE;
```

5.3.6. Input expression base type. The input expression—the expression over which sapply runs—is also typedef'ed for convenience:

```
typedef Rcpp::VectorBase<RTYPE, NA, T> VEC;
```

5.3.7. Output expression base type. In order to be part of the *Rcpp sugar* system, the type generated by the Sapply class template must inherit from VectorBase.

```
template <int RTYPE, bool NA,
          typename T, typename Function>
class Sapply : public VectorBase<
    Rcpp::traits::r_sexptype_traits<
        typename
        ::Rcpp::traits::result_of<Function>::type
    >::rtype,
    true,
    Sapply<RTYPE,NA,T,Function>
>
```

The expression built by Sapply depends on the result type of the function, may contain missing values, and the third argument is the manifestation of the *CRTP*.

5.3.8. Constructor. The constructor of the Sapply class template is straightforward, it simply consists of holding the reference to the input expression and the function.

```
Sapply(const VEC& vec_, Function fun_):
    vec(vec_), fun(fun_){}

private:
    const VEC& vec;
    Function fun;
```

5.3.9. Implementation. The indexing operator and the `size` member function is what the `VectorBase` expects. The size of the result expression is the same as the size of the input expression and the ith element of the result is simply retrieved by applying the function and the converter. Both these methods are inline to maximize performance:

```
inline STORAGE operator[](int i) const {
    return converter_type::get(fun(vec[i]));
}
inline int size() const {
    return vec.size();
}
```

6. Summary

TBD

References

Abrahams D, Gurtovoy A (2004). *C++ Template Metaprogramming: Concepts, Tools and Techniques from Boost and Beyond*. Addison-Wesley, Boston.

Eddelbuettel D, François R (2011). "Rcpp: Seamless R and C++ Integration." *Journal of Statistical Software*, **40**(8), 1–18. URL http://www.jstatsoft.org/v40/i08/.

Eddelbuettel D, François R, Allaire J, Ushey K, Kou Q, Russel N, Chambers J, Bates D (2019a). *Rcpp: Seamless R and C++ Integration*. R package version 1.0.3, URL http://CRAN.R-Project.org/package=Rcpp.

Eddelbuettel D, François R, Bates D, Ni B (2019b). *RcppArmadillo: Rcpp integration for Armadillo templated linear algebra library*. R package version 0.9.800.1.0, URL http://CRAN.R-Project.org/package=RcppArmadillo.

R Core Team (2018). *Writing R extensions*. R Foundation for Statistical Computing, Vienna, Austria. URL http://CRAN.R-Project.org/doc/manuals/R-exts.html.

Sanderson C (2010). "Armadillo: An open source C++ Algebra Library for Fast Prototyping and Computationally Intensive Experiments." *Technical report*, NICTA. URL http://arma.sf.net.

Vandevoorde D, Josuttis NM (2003). *C++ Templates: The Complete Guide*. Addison-Wesley, Boston.
