# Supplementary material for: A biomathematical model of human erythropoiesis and iron metabolism
Source: Sci Rep. 2020 May 25;10:8602. doi: 10.1038/s41598-020-65313-5 (PMC7248076; doi:10.1038/s41598-020-65313-5)
Supplement: Supplementary file 5 — A biomathematical model of human erythropoiesis and iron metabolism: Simulation Model. [file 41598_2020_65313_MOESM5_ESM.zip › ErythroShort/TitlePage.docx]

A biomathematical model of human erythropoiesis and iron metabolism: Simulation Model

Sibylle Schirm^1^ and Markus Scholz^1;^*_

1 Institute for Medical Informatics, Statistics and Epidemiology, University of Leipzig, Leipzig, Germany

* [markus.scholz@imise.uni-leipzig.de](mailto:markus.scholz@imise.uni-leipzig.de)

# Starting the Model Simulation

Open “RunErythroShort.R” , e.g. in RGui Rx64 3.4.2

1. Enter the project path line, for example projectpath<-"C:/MyProjects/ErythroShort"
2. Select one of the data sets for simulation by removing the comment "#" from the corresponding line. The project contains the following data from publications:

\# path_data_definitions <- paste(projectpath,"/Souillard/Paper",sep = "")

\# path_data_definitions <- paste(projectpath,"/CHOP/Paper",sep = "")

\# path_data_definitions <- paste(projectpath,"/Kiss_c_Fe/Paper",sep = "")

\# path_data_definitions <- paste(projectpath,"/Rutherford600/Paper",sep = "")

\# path_data_definitions <- paste(projectpath,"/Rutherford300/Paper",sep = "")

\# path_data_definitions <- paste(projectpath,"/Rutherford400/Paper",sep = "")

3. Run lines 18-34.
4. Produce a graphic output with line 36.
5. You can produce a numerical results structure “ResultsStruct” with line 38:

ResultsStruct<-SimulErythroModel(x=main_struct1$phiopt,ind_biol=1,ind_indiv=1,main_struct=main_struct1)

This structure has several fields. The relevant ones are the following:

a. Scompl	- complete matrix of variables for each time step.
b. Tcompl - all time steps
c. fitted adjustments for the corresponding data points for each data class
1. values
2. time
